# Translating Formative Research into Intervention Content: Experiences with Face Washing for Trachoma Control in Rural Ethiopia

**DOI:** 10.3390/bs15030355

**Published:** 2025-03-13

**Authors:** Claire Collin, Adam Biran, Alexandra Czerniewska, Demitu Legesse, Meseret Guye, Asanti Ahmed Mume, Edao Sinba Etu, Aida Abashawl, Wondu Alemayehu, Sarity Dodson, Oumer Shafi Abdurahman, Esmael Habtamu, Dereje Adugna, Matthew J. Burton, Anna Last, Katie Greenland

**Affiliations:** 1 Department for Disease Control, London School of Hygiene & Tropical Medicine (LSHTM), London WC1E 7HT, UK; ccollin.contact@gmail.com (C.C.); alexandra.czerniewska@gmail.com (A.C.); 2 Population Health Sciences Institute, Newcastle University, Newcastle upon Tyne NE2 4AX, UK; adam.biran@newcastle.ac.uk; 3 The Fred Hollows Foundation Ethiopia, Addis Ababa 23360/1000, Ethiopia; dhika@hollows.org (D.L.); bekakoo80@gmail.com (M.G.); asantiahmed7@gmail.com (A.A.M.); oshafi@hollows.org (O.S.A.); 4 Berhan Public Health & Eye Care Consultancy, Addis Ababa P.O. Box 6307, Ethiopia; edao.sinba@mwu.edu.et (E.S.E.); aabashawl@gmail.com (A.A.); walemayehu@berhan-health.org (W.A.); 5 The Fred Hollows Foundation, Melbourne, VIC 3000, Australia; sdodson@hollows.org; 6 International Centre for Eye Health, Clinical Research Department, London School of Hygiene & Tropical Medicine (LSHTM), London WC1E 7HT, UK; esmael.ali@lshtm.ac.uk (E.H.); matthew.burton@lshtm.ac.uk (M.J.B.); anna.last@lshtm.ac.uk (A.L.); 7 Oromia Regional Health Bureau, Oromia, Ethiopia; sifdere@gmail.com; 8 National Institute for Health Research Biomedical Research Centre for Ophthalmology at Moorfields, Eye Hospital NHS Foundation Trust and UCL Institute of Ophthalmology, London EC1V 2PD, UK

**Keywords:** behaviour change, intervention, trachoma, face washing

## Abstract

Face washing for trachoma, like most public health improvements, necessitates behaviour change, yet traditional educational interventions frequently fail to achieve this goal. Behavioural science frameworks offer guidance to develop alternative types of interventions, helping to translate formative research and insights about the target population and behavioural determinants into more effective strategies. This paper outlines the outputs and decision-making underlying the five-stage process we followed to translate formative research findings into intervention activities and materials: (1) synthesising formative research findings into a creative brief to guide intervention development; (2) selecting behaviour change techniques (BCTs) to address key behavioural targets; (3) selecting an overarching intervention concept; (4) developing intervention content; and (5) finalising the intervention’s Theory of Change. This paper presents our experiences and reflections on the intervention design process, using a practical example of a face washing intervention for trachoma control. The intervention was designed for delivery in the Stronger SAFE trial in rural Oromia, Ethiopia (ISCRTN 40760473).

## 1. Introduction

Behaviour change interventions in the Water, Sanitation, and Hygiene (WASH) sector have progressed from educational and community development approaches to incorporate growing insights from behavioural science. A feature of this is the application of structured methods to translate behavioural theory into intervention components (Aunger & Curtis, 2016; Dreibelbis et al., 2013; Michie et al., 2011; Mosler, 2012). A key step in this process is the conduction of formative research, which involves the collection of data to identify the intervention’s target audience and understand their needs, behaviours, and the context in which they operate (Gittelsohn et al., 2006; Glasgow & Linnan, 2008). The main purpose of formative research is to understand the variety of personal, cognitive, social, cultural, and structural factors that influence individual behaviours in a given context and the processes that can be mobilised to develop effective interventions that bring about change in these factors at different levels (e.g., individual, interpersonal, organisational, environmental).

A growing body of WASH literature documents formative research studies rooted in behavioural science, implementation studies through process evaluations, as well as quantitative outcome evaluations following the method of scientific enquiry, often in the form of a randomised controlled trial (Biswas et al., 2021; D’Mello-Guyett et al., 2020; Greenland et al., 2017; Kuhl et al., 2021; Ogutu et al., 2022; Panulo et al., 2022; Sule et al., 2022). However, less attention has been given to the intervention design process, which translates formative research into practical materials and activities for implementation and evaluation through process and outcome evaluation.

Intervention design is a creative process which encompasses both content and delivery strategies. While increasingly informed by formative research and behavioural science frameworks, it is also shaped by prior experience, pragmatic decision-making, researchers’ experiential knowledge, and contextual adaptations, constraints, and opportunities (Tidwell et al., 2019). The limited systematic reporting of this design process hinders experience sharing and learning among behaviour change researchers and practitioners.

This paper addresses the gap between formative research and intervention implementation and evaluation by documenting and reflecting on the creative process of designing a face washing intervention for trachoma control.

Despite widespread implementation of the WHO-endorsed SAFE strategy—Surgery to treat blinding trachoma, Antibiotic mass drug administration, Facial cleanliness, and Environmental improvement to reduce the pool of infection and slow transmission—(World Health Organization, 2020; Taylor et al., 2014), trachoma remains a major public health challenge in Ethiopia, which accounts for a disproportionate 59% of the global at-risk population (World Health Organization, 2024). Facial cleanliness interventions for trachoma prevention have primarily relied on educational approaches (Delea et al., 2018; Freeman et al., 2022); trials of such interventions have not demonstrated a significant impact on trachoma outcomes to date (Abdou et al., 2010; Aragie et al., 2022; Ejere et al., 2015). In this article, we present the creative design process used to translate formative research findings into a face washing intervention for trachoma control in rural Oromia, Ethiopia. We detail five distinct stages of the intervention’s design: Stage 1 involved developing a creative brief by synthesising formative research findings; Stage 2 entailed selecting behaviour change techniques (BCTs) to address behavioural targets; Stage 3 focused on choosing an overarching intervention concept; Stage 4 comprised developing intervention content, including designing, testing, and refining activities and materials; and Stage 5 involved finalising the intervention’s Theory of Change (ToC) (De Silva et al., 2014; Weiss, 1995). For each stage, we describe the outcome and the decision-making process. The Methods section details the decision-making within each stage, while the Results section presents the tangible outputs produced by each creative stage.

## 2. Materials and Methods

Intervention design was guided by Behaviour Centred Design (BCD) (Aunger & Curtis, 2016; Aunger et al., 2017) and Risk, Attitude, Norm, Ability, and Self-regulation (RANAS) models (Mosler, 2012), which had also guided our formative research. These models have been used to develop successful behaviour change interventions in the WASH sector (Biran et al., 2014; Friedrich et al., 2020; Inauen & Mosler, 2014), and offer complementary, structured frameworks for developing engaging, contextually relevant interventions to address key determinants of behaviour. The following sections detail the methods used and decisions made during each stage of the intervention design process.

### 2.1. Stage 1—Developing the Creative Brief: Synthesising Formative Research

The design of this face washing intervention was conducted within specific intervention parameters established by the Stronger SAFE cluster-randomised trial (Stronger SAFE, ISCRTN 40760473), which evaluated a comprehensive package of trachoma control interventions. These parameters mandated face-to-face community-based implementation through local health volunteers and community leaders; a structured two-year delivery timeline aligned with the timeframe for the trial; delivery at the *garee* (sub-village administrative unit) level; employment of multi-modal delivery strategies; integration within existing public health infrastructure; adaptation for low literacy populations with limited mass media access; incorporation of mechanisms for sustained behaviour maintenance; and universal household inclusion within intervention communities.

To inform the intervention design, a series of preliminary and formative research studies were conducted, exploring local determinants of face washing and contextual factors likely to influence intervention implementation (Czerniewska et al., 2020; Greenland et al., 2019, 2022; Last et al., 2020; Shafi Abdurahman et al., 2023).

The ‘creative brief’, the output of the synthesis of data from these studies, provided the foundation for the intervention design process (Aunger et al., 2017). Recognising the overlap between formative research and intervention design, this stage details the synthesis process to provide context for the subsequent intervention development.

The creative brief was developed through a collaborative process involving qualitative synthesis of research findings supplemented by contributions from researchers and key stakeholders with expertise in face washing interventions. We identified key themes and patterns related to face washing behaviour and its determinants and organised these findings according to the five behavioural factors of the RANAS framework (risk, attitude, norm, ability, and self-regulation), i.e. the ’perceptions, thoughts, feelings, and beliefs that influence the practice of a behaviour’ within a specific context (Mosler, 2012). Through this process, we distilled the research findings into actionable insights for the intervention design.

The creative brief served as a roadmap for the intervention design, ensuring that a locally relevant intervention package was developed and all creative efforts were aligned with the intervention’s goals and the pre-defined intervention parameters. It detailed the formative research findings organised by behavioural factor and defined the target behaviour specifying who, when, where, and how face washing should be performed.

### 2.2. Stage 2—Selecting Behaviour Change Techniques (BCTs)

Stage 2 involved selecting strategies to bring about change in behavioural determinants identified in the brief, known as Behaviour Change Techniques (BCTs). Previous work has listed and categorised these techniques based on reviews of the behaviour change literature and expert opinion, mapping them to specific behaviour change problems they might address (Michie et al., 2013). 

To select BCTs, we reformulated the potential determinants of behaviour outlined in the creative brief into specific targets that the intervention would address. We then utilised the RANAS catalogue of BCTs to select techniques for the intervention (Mosler, 2016). (When multiple BCTs were associated with a given factor, several were selected to enhance the likelihood of influencing that given factor. Techniques perceived as coercive, stigmatising (e.g., comparing or shaming neighbours’ behaviours), or reinforcing negative power dynamics were not selected.

### 2.3. Stage 3—Choosing an Overarching Intervention Concept

Following the BCD approach, we then chose an ‘umbrella’ concept to unify intervention activities under a compelling narrative linked to a motive that resonated with the community (Aunger et al., 2017; Czerniewska et al., 2023). The purpose was to enhance audience adherence to the intervention package, foster audience engagement, and improve the memorability of key intervention messages.

Four potential motives—“pair-bond love”, “nurture”, “status”, and “dignity”—were developed into short narratives and tested in eight focus group discussions (FGDs) with community members (four male groups, four female groups, six to eight participants per group) and five interviews with female primary caregivers. Participants listened to five- to ten-minute *Afan Oromo* audio recordings of the narratives, individually ranked them by memorability, likeability, and character relatability, and provided qualitative feedback on their rankings. They were also asked to describe daily activities associated with the different motives.

FGD and interview audio recordings were transcribed and analysed alongside field notes, focussing on the investigated attributes and perceived links to personal hygiene behaviours.

### 2.4. Stage 4—Developing Intervention Content

Intervention activities and materials were designed to integrate selected BCTs within a coherent package aligned with the overarching concept. This was achieved through an iterative process of ideation, prototyping, and refinement, involving stakeholder engagement and community feedback. A local creative agency, Spotlight, was hired to design the visuals and infographics used in intervention activities.

A five-day stakeholder workshop in Shashemene, Ethiopia, generated initial ideas for intervention activities and materials, with participation from federal and local government representatives, local health workers and community members, representatives from other NGOs working in the local areas, our field team, and creatives from a consortium of local and regional agencies. Working in small groups, these stakeholders shortlisted six promising ideas and developed prototypes.

Intervention content was further refined into activities and events through testing in 18 FGDs with children and female caregivers to assess understandability, acceptability, and relatability. Each activity was developed to target the specific intervention targets identified in Stage 2 and integrated one or multiple BCTs. Whenever possible, the “dignity” motive was used to frame activity content and the target behaviour was reinforced at least once during each event. A multi-event format was designed to maximise intensive contact points with the target population, while adhering to the intervention parameters. Local health volunteers, who were respected in the community, were involved in intervention delivery to legitimise it and support implementation. This approach leveraged existing outreach structures, promoting intervention buy-in, especially among community leaders.

The full intervention package was piloted three times under real-world conditions to assess coherence, flow, and pacing. After each round, participant feedback was reviewed, observations were shared, and the intervention package was refined for subsequent piloting.

### 2.5. Stage 5—Finalising the Intervention’s Theory of Change

A draft Theory of Change (ToC) was developed at the start of the design process outlining the outcomes, intermediate outcomes hypothesised to influence them, and the selected BCTs to drive measurable changes in these intermediate outcomes and ultimately lead to change in the target behaviour (De Silva et al., 2014; Weiss, 1995). This draft ToC was iteratively refined throughout intervention design to ensure intervention content was supported by hypothesised behaviour change pathways. The ToC was finalised after all intervention activities had been developed, piloted, and validated for inclusion in the intervention package. This finalisation incorporated inputs from multiple stakeholders with different expertise in behaviour change interventions, including local and international academics and WASH practitioners.

## 3. Results

### 3.1. Stage 1—Developing the Creative Brief: Synthesising Formative Research

Table 1 presents an overview of the insights from the formative research and preliminary studies, illustrating how findings were mapped to the RANAS behavioural factors. Given that face washing was typically limited to the early morning, rarely involved soap, and was unsupervised for young children (who are key in trachoma transmission), the target behaviour was defined as *faces (and hands) of the whole family (particularly pre-school children) to be thoroughly washed with soap three times a day: morning after waking, before lunch, and before the evening meal.* This definition prioritised pre-school children, increased washing frequency, and emphasised soap use and caregiver supervision for effective discharge removal.

Table 1 also highlights key insights from the formative research, including resource limitations such as soap and water, which pointed to the need for accessible and creative solutions operating within these constraints. Drawing on these actionable insights, the creative brief proposed that the face washing intervention delivered in this context should emphasise the benefits of soap while encouraging efficient water usage; establish a daily face washing with soap routine for children; promote the construction of accessible, high-quality wash stations with soap; support caregiver supervision to improve removal of discharge, which is known to transmit trachoma; and shift perceptions of discharge as a trachoma risk, using emotional motivators to drive behaviour change.

### 3.2. Stage 2—Selecting Behaviour Change Techniques (BCTs)

We identified 20 intervention targets within the five categories of the RANAS approach that represented the specific perceptions, thoughts, feelings, and beliefs that the intervention would need to modify to achieve the desired change in face washing behaviour. ‘Risk’ intervention targets included understanding the importance of face washing for trachoma control and strengthening the perception that ocular and nasal discharge are both disgusting and dangerous to health. Key ‘Attitudinal’ targets included perceptions that soap is important for face washing, wash stations make it easier to wash faces, and the amount of water and soap required for face washing is manageable, and the motivation to prioritise these resources for face washing. Key ‘Normative’ targets included the belief that face washing with soap must be performed several times a day to achieve truly clean faces throughout the year, and that such behaviours were expected by the community. Key ‘Ability’ intervention targets included the ability to construct and maintain a wash station stand, the ability to wash the faces of pre-school children, self-efficacy in acting as a role model, and taking responsibility for young children’s hygiene. Finally, key ‘Self-regulation’ targets were the integration of the target behaviour into daily routine, the consistent provision of water and soap for face washing, and the capacity to trouble-shoot seasonal factors to continue to prioritise these resources.

Table 2 presents these intervention targets along with 29 BCTs from the RANAS catalogue that were mapped to these targets.

### 3.3. Stage 3—Choosing an Overarching Concept

The “pair-bond love” narrative (linking face washing to marital relationships) resonated well with the community. However, we identified potential risks of reinforcing negative gender stereotypes or poor treatment of women using this narrative. The “status” narrative (highlighting social standing with a negative framing around public shaming) was not acceptable in the community. The “nurture” (focused on family well-being) was less engaging, as participants struggled to identify a clear emotional driver.

The “dignity” narrative demonstrated strong performance, with participants viewing dignity as fundamental to gaining respect and social standing, and establishing a credible link to personal hygiene of both adults and children, as illustrated by the following quote:

“*Children come from the family, so if the family is dignified the children will be dignified*”.

(Female caregiver, individual household interview on the concept of dignity, January 2020)

Both the “pair-bond love” and “dignity” narratives scored highly across comprehension, acceptability, reliability, and perceived connection to face washing (Table 3). However, to mitigate potential negative side effects associated with the “pair-bond love” theme, dignity was ultimately chosen as the intervention concept.

**Figure 1:**
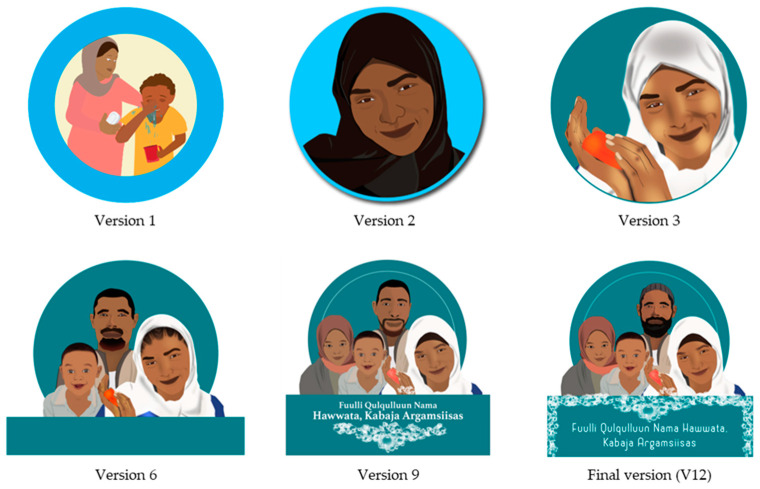
Evolution of the *Faces of Dignity* intervention logo. “*Fuulli Qulqulluun Nama Hawwata, Kabaja Argamsiisas*” translates as “*A clean face is attractive, it is also dignifying*”.

The intervention was named the *Faces of Dignity* intervention, with the tagline “*A clean face is attractive, it is also dignifying*”, exemplified by the image of a dignified family, which was refined through multiple rounds of community feedback (Figure 1). This image visually represented the intervention’s central narrative and served as its logo, being easily recognisable, memorable, and aspirational for the audience, reminding them about the target behaviour promoted by the intervention. The logo was displayed on the intervention banner and materials.

### 3.4. Stage 4—Developing Intervention Content

#### 3.4.1. Intervention Activities

**Figure 2:**
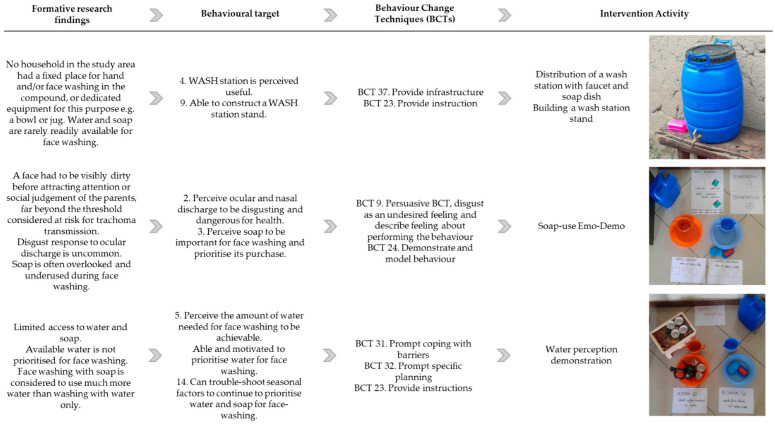
Three examples illustrating the theory-informed design of intervention activities for the *Faces of Dignity* intervention. BCT: Behaviour Change Technique.

Between April 2019 and February 2021, 33 intervention activities were designed based on ideas proposed in the stakeholders’ meeting and refined with community feedback. Figure 2 illustrates the development process of three of these activities, showing how key formative research findings were categorised by RANAS behavioural factors (Stage 1), reformulated into behavioural targets and linked to relevant BCTs (Stage 2), and translated into intervention activities.

#### 3.4.2. Intervention Package

The 33 intervention activities were grouped into nine events. The full intervention package of activities and materials is presented in Table 4. The first five events—the *Community Event*, two *Family Fora*, and two *Household Visits*—formed the intensive behaviour change package, which was followed by four *Seasonal Reinforcement Events* spread out over the two years that the trial was planned to last. Detailed intervention manuals outlining the content and materials required for each event were developed to aid training and ensure intervention fidelity and standardisation throughout implementation (see Supplementary Materials).

The *Community Event*, designed to engage all residents of the three *garees* (administrative units of approximately 90 households), involved two actors, local influential leaders, and health volunteers. A 20-min drama with a life-sized child puppet was used to present different face washing scenarios and outcomes, introducing parents as hygiene role models and promoting the target behaviour as a way of maintaining family dignity and social acceptance. The event culminated in a collective pledge led by influential community leaders, following which wash stations with a faucet, soap, and soap dishes were distributed to all households to make face washing easier and cue behaviour.

One trained activator and one health volunteer delivered the two *Family Fora*, one week apart to groups of five neighbouring households. The first *Family Forum* aimed to build knowledge, skills, and motivation for face washing, and inspire households to commit to constructing a wash station stand to facilitate face washing and habit formation. The second *Family Forum* focused on addressing practical challenges and motivation related to wash station (stand) construction and usage.

*Household Visits* intended to provide more tailored feedback and support to overcome face washing barriers and maintain wash stations, with rewards provided to signal completion of the intensive programme. Two health volunteers and the activator conducted the *Household Visits*, one and two weeks after the second *Family Forum*. Using health volunteers who were already known and respected in the community helped to legitimise the intervention. It also leveraged the existing structure for community outreach.

Following this intensive phase, four *Seasonal Reinforcement Events* targeting groups of 10 households were led by health volunteers. These events aimed to address specific seasonal challenges, provide tips on wash station maintenance, and encourage continuation of new face washing practises, highlighting the key roles played by adults in the family in modelling behaviour and continuously provisioning water and soap at the stations.

### 3.5. Stage 5—Finalising the Intervention’s Theory of Change

**Figure 3:**
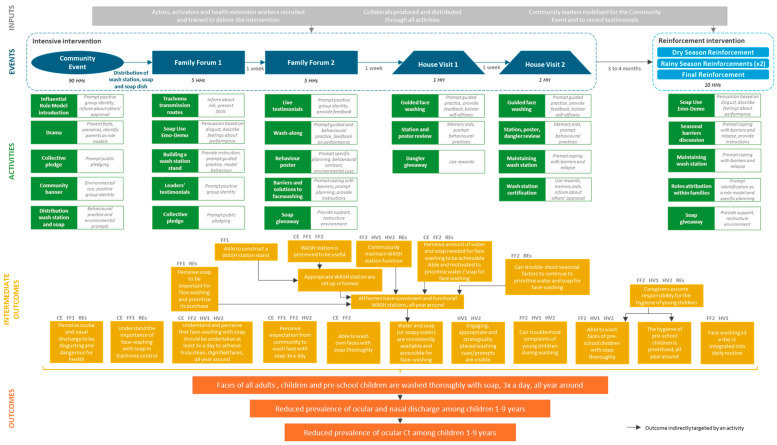
Theory of Change for the *Faces of Dignity* intervention. The event that includes activities targeting each intermediate outcome is indicated. CE = Community Event; FF1 = Family Forum 1; FF2 = Family Forum 2; HV1 = House Visit 1; HV2 = House Visit 2; REs = Reinforcement Events.

The intervention’s final Theory of Change is presented in Figure 3. The schematic illustrates how intervention activities and associated BCTs are employed to modify specific behavioural targets (intermediate outcomes) which are hypothesised to increase face washing with soap (outcome). This increased face washing, in turn, is expected to reduce oculo-nasal discharge on children’s faces, thereby contributing to a decrease in trachoma transmission within the community.

## 4. Discussion

This paper describes five key stages in the development of behaviour change interventions, using the example of a face washing intervention to improve the trachoma control strategy in a trachoma-endemic setting, in rural Oromia, Ethiopia.

Intervention design was informed by extensive formative research and grounded in two behaviour (change) theories, the BCD and RANAS approaches, both recognised approaches used to develop WASH behaviour change interventions in low-income countries (De Buck et al., 2017). These intervention design guidelines and theoretical frameworks enabled us to structure the creative process and organise our formative research findings to address a range of behavioural factors likely to drive face washing behaviour in our setting (Michie, 2008), a consideration often overlooked in face washing interventions for trachoma control (Delea et al., 2018).

Although structured into different steps, our creative design process shares similarities with other documented hygiene intervention development frameworks, notably the process outlined by Arriola et al. for designing interventions to improve nutrition and WASH behaviours (Jacob Arriola et al., 2020). A key distinction in our approach is that we treated formative research as a prerequisite rather than an integrated component of the creative process. We initiated our creative process by synthesising research findings into a comprehensive creative brief, which specified intervention parameters, the target behaviour, and behavioural determinants that could be modified by an intervention, elements that Jacob Arriola et al. addressed as a separate second step. The timing of Theory of Change (ToC) development also differed. While Jacob Arriola et al. articulated the ToC early in their process, our ToC model was drafted at the outset and iteratively refined throughout intervention design. While we employed different behaviour change frameworks, both creative processes followed a similar sequence: identifying behavioural targets, selecting behaviour change techniques (BCTs), developing intervention activities, and refining intervention packages using various data collection methods. However, our approach makes two notable contributions to the literature: first, by documenting the selection of an overarching intervention concept aligned with the BCD model; and second, by detailing how intervention activities were derived from formative research, linked to specific behavioural determinants, mapped to relevant BCTs, and translated into concrete activities. This systematic translation process, which provides a clear trail from formative research to implementation, represents an advancement on existing descriptions of intervention design processes.

These variations in intervention design structuring, however, highlight an important reality: despite the availability of frameworks detailing steps needed to be undertaken to translate theory into practical interventions (French et al., 2012; Michie, 2008; Susan Michie et al., 2011; O’Cathain et al., 2019), the design process remains complex and often requires making decisions that depart from established frameworks. Intervention design thus entails a series of decisions, informed by a combination of methodological guidance, empirical evidence, prior knowledge, and professional experience. The principal challenge is the precise documentation of decision-making to ensure transparency, thereby enabling the broader scientific and practitioner community to comprehend and replicate the design process. This documentation is essential for advancing both the theoretical understanding and practical development of behavioural interventions.

A key challenge in documenting intervention design lies in capturing its inherently iterative nature (O’Cathain et al., 2019). Unlike the straightforward linear process that the description in stages might suggest, intervention design is dynamic and continually evolves as new insights and challenges arise. While this iterative approach allows for flexibility and adaptability, it often requires prolonged periods of field testing, ongoing intervention refinement, and, at times, lengthy decision-making processes. The development of the *Faces of Dignity* intervention took 22 months (during the COVID-19 pandemic). The pandemic did not alter the content of our intervention, but the extended timelines of intervention development might raise concerns about the applicability and timeliness of pre-existing evidence underpinning the design process in a rapidly changing context.

Another important reflection concerns the co-design process. Co-production ensures interventions are culturally relevant, sustainable, and owned by the communities they serve. It creates trust, builds capacity, and increases the likelihood of long-term impact (Vargas et al., 2022). Co-production is relatively uncommon in low-income countries (Singh et al., 2023). The COVID-19 pandemic and local travel restrictions linked to political unrest hindered our ability to engage as closely with communities as we desired. These factors restricted full community involvement in intervention development and feasibility testing. Ideally, a more advanced co-production process would have involved local communities more actively in the selection of intervention priorities from the outset.

One of the key strengths of this work was the development of a robust Theory of Change (De Silva et al., 2014). The Theory of Change provided a clear and comprehensive vision of the intervention activities structured around specific BCTs and the hypothesised causal pathways through which the intervention is expected to influence face washing behaviour. Furthermore, the Theory of Change facilitated the identification of key indicators that were assessed in the later process and outcome evaluations.

Despite identifying water availability as a major barrier to face washing in our formative research, budgetary and logistical constraints prevented direct intervention in water access. Instead, *Family Fora* activities sought to shift perceptions of water scarcity by demonstrating that face washing with soap requires less water than commonly assumed and providing strategies for conserving water for this purpose. This limitation highlights how interventions are shaped by initial project parameters and contextual constraints, reinforcing that intervention development necessarily involves decisions extending beyond strictly defined scientific methodologies.

To conclude, this paper represents one of few attempts to document the transition from formative research to intervention implementation and evaluation by detailing the intervention design process. It outlines experiences that may benefit other researchers and practitioners attempting to synthesise formative research findings, choose behavioural targets and behaviour change techniques, develop overarching concepts to unify interventions, and develop intervention’s activities and materials, alongside refining the intervention’s Theory of Change. Finally, this paper also highlights the importance of recognising the potential limitations of intervention design processes, which, despite being labelled as theory-based and scientific, still involve significant subjective decision-making, further influenced by contextual and project-specific constraints. We provide a practical example of how a rigorously designed intervention can be developed using explicit behaviour change theory, informed by empirical formative research findings through a transparent decision-making process.

The resulting intensive intervention was delivered over four to six weeks in 34 rural communities between January and April 2022. Reinforcement Events were delivered around the start of the dry and rainy seasons in September 2022, January 2023, September 2023, and January 2024. Results of the process and outcome evaluation are forthcoming.

## Figures and Tables

**Table 1:** Categorisation of formative research findings on face washing behaviour by RANAS behavioural factors.

RANAS Behavioural Factor	Formative Research Findings on Face Washing Behaviour and Its Potential Determinants
Risk	Awareness of trachoma: common; awareness of link between hygiene and trachoma prevention: moderate; little fear of blindness from trachoma
Attitude	Disgust response to ocular discharge: uncommon; soap use for body washing: uncommon; reward/recognition for children who wash: none; pleasure experienced from washing: refreshment when dusty; water needed: more water needed if soap is used (1 L vs. 0.5 L per person per wash) Emotional drivers: Social judgement/affiliation motive: cannot publicly judge others, but mothers fear being judged; celebrity/role model/status motive: unimportant; nurture motive: weak, fathers interested in engaging with health activities and issues
Norm	Face washing with soap practises in the morning only; use of soap: not expected; being clean: expected at school but not at home; handwashing before meals: expected, low emphasis on face washing; gossiping or visibly judging neighbours: unacceptable; role models do not use soap; responsibility for hygiene of adults and children: self; responsibility for hygiene of very young children: mothers and occasionally fathers, limited involvement of fathers in childcare; responsibility for water collection: mothers or older children
Ability	Socioeconomic status: poor and very poor; water taps at home: uncommon, water storage in jerry cans; washing stations: uncommon; capability of small children to perform effective washing with minimal water: very limited; children resist washing which makes it difficult for mothers; discomfort/pain associated with soap in eyes, soap use dries the skin
Self-regulation	Responsibility for financial decisions about household purchases: fathers; responsibility for digging holes/building infrastructure: fathers; fathers oversee the activities of mothers; limited access to water and soap; high water scarcity (especially in dry season); soap availability: can be purchased at local market affordably; washing prompts: waking, evening meal; physical washing prompts: uncommon; washing equipment: plastic bowls/buckets/teapots/cups/cans, fabric use for face drying: uncommon

**Table 2:** RANAS Behaviour Change Techniques (BCTs) mapped against intervention targets.

RANAS Behavioural Factors	Intervention Target	RANAS Behaviour Change Techniques
**Risk factors**		
Factual knowledge	1. Understand the importance of face washing with soap in trachoma control.	BCT 1. Present facts BCT 2. Present scenarios
Vulnerability	2. Perceive ocular and nasal discharge to be disgusting and dangerous for health.	BCT 3. Inform about and assess personal risk
**Attitudinal factors**		
Beliefs about costs and benefits	2. Perceive ocular and nasal discharge to be disgusting and dangerous for health. 3. Perceive soap to be important for face washing and prioritise its purchase. 4. WASH station is perceived to be useful. 5. Perceive amount of water and soap needed for face washing to be achievable. Able and motivated to prioritise water/soap for face washing.	BCT 5. Inform about and assess costs and benefits BCT 6. Use subsequent reward BCT 7. Self-incentive BCT 8. Inform about and assess social and environmental consequences BCT 9. Describe feelings about performing and about consequences of the behaviour
**Normative factors**		
Others’ behaviour	6. Perceive expectation from community to wash face with soap 3× a day. 7. Understand and perceive that face washing with soap should be undertaken at least 3× a day to achieve truly clean, dignified faces, all year around.	BCT 11. Inform about others’ behaviour BCT 13. Prompt public pledging
Others’ (dis)approval	6. Perceive expectation from community to wash face with soap 3× a day. 7. Understand and perceive that face washing with soap should be undertaken at least 3× a day to achieve truly clean, dignified faces, all year around.	BCT 15. Inform about others’ approval/disapproval
Personal norms	7. Understand and perceive that face washing with soap should be undertaken at least 3× a day to achieve truly clean, dignified faces, all year around. 8. Caregiver assume responsibility for the hygiene of young children.	BCT 17. Provide a positive group identity and use in-group terms BCT 18. Prompt identification as role model
**Ability factors**		
Action knowledge	9. Able to construct a WASH station stand.	BCT 23. Provide instruction
Confidence in ability	10. Able to wash faces of pre-school children with soap thoroughly. 11. Can troubleshoot complaints of young children during washing. 12. Able to wash own faces with soap thoroughly.	BCT 24. Demonstrate and model behaviour BCT 25. Prompt guided practice BCT 26. Prompt behavioural practice BCT 27. Organise social support BCT 28. Set graded tasks/goals
Confidence in continuation	13. Continuously maintain WASH station function.	BCT 29. Reattribute past successes and failures
Confidence in recovering	14. Can trouble-shoot seasonal factors to continue to prioritise water and soap for face washing.	BCT 31. Prompt coping with relapse
**Self-regulation factors**		
Action planning	15. Face washing 3× a day is integrated into daily routine.	BCT 32. Prompt specific planning
Action control	16. Appropriate WASH station are set up in homes. 17. All homes have convenient and functional WASH stations, all year around. 18. Water and soap (or soapy water) are consistently available and accessible for face washing.	BCT 33. Prompt (self) monitoring of behaviour BCT 34. Provide feedback on performance BCT 35. Highlight discrepancy between set goal and actual behaviour
Barrier planning	14. Can trouble-shoot seasonal factors to continue to prioritise water and soap for face washing. 19. The hygiene of pre-school children is prioritised, all year around.	BCT 36. Prompt coping with barriers BCT 37. Restructure the social and physical environment
Remembering	20. Engaging, appropriate and strategically placed washing cues/prompts are visible.	BCT 38. Use memory aids and environmental prompts
Commitment	15. Face washing 3× a day is integrated into daily routine. 19. The hygiene of pre-school children is prioritised, all year around.	BCT 39. Prompt goal setting BCT 40. Prompt to agree on a behavioural contract

**Table 3:** Comparative evaluation of motivational narratives for face washing behaviour.

Emotional Driver	Comprehension	Acceptability	Relatability	Perceived Link to Face Washing
Pair-bond love	High	High	High	High
Nurture	Low	Moderate	Moderate	Moderate
Status	High	Low	Moderate	High
Dignity	High	High	High	High

**Table 4:** Overview of the *Faces of Dignity* intervention.

Event	Audience	Implementers	Purpose	Content	Timing	Duration
Community Event	90 households from three garees *	2 Actors 2 Health Volunteers 2 Community leaders	Raise awareness and credibility of the *Faces of Dignity* intervention, increase buy-in, and initiate face washing	A 20 min drama presenting *scenarios* of face washing behaviours, highlighting parents as role models and soap use as essential for family dignity. A *collective pledge*, led by community leaders, *prompting public commitment* and *creating normative expectations* to adopt the target behaviour. Distribution of wash stations with a faucet and soap to facilitate *ability* to perform the behaviour.	First event	1 h
Family Forum 1	5 neighbouring households	1 Activator 1 Health Volunteer	Build knowledge, skills, and motivation for face washing. Empower households to construct wash stations to aid habit formation.	Description of trachoma transmission routes to increase knowledge. Soap Use Emo Demo to elicit disgust as a driver for behaviour change. Distribution of an *instruction flyer* to guide households in building an accessible washstand for their wash station. Pre-recorded testimonials from role models *informing about expected behavioural practise*. *Collective pledge* with participants committing to building the station and ensuring three daily washes.	1 week post Community Event	1 h and 30 min
Family Forum 2	5 neighbouring households	1 Activator 1 Health Volunteer	Overcome early barriers related to wash station construction and use. Emphasise the need and build capacity to practise the target behaviour.	Live testimonials for participants to share experiences and provide feedback to develop tailored solutions. Wash-along activity to reinforce self-efficacy. Water prioritisation activities, soapy water demonstration, and distribution of a bar of soap to *address barriers to sustained behaviour change*, such as water and soap availability. Distribution of the “Dignified Day” poster as a behavioural reminder.	1 week post Family Forum 1	1 h and 30 min
Household Visit 1	1 household	1 Health Volunteer	Provide personalised support to practise the target behaviour.	Wash station assessment with tailored feedback. Review of the “Dignified Day” poster. Distribution of a dangler serving as a reminder for face washing.	1 week post Family Forum 2	20 to 30 min
Household Visit 2	1 household	1 Activator	Provide ongoing and personalised support to practise the target behaviour and to maintain the wash station.	Face washing with soap demonstration to reinforce behavioural practise and self-efficacy. A short video on wash station maintenance to prompt coping with barriers and behavioural relapse. Distribution of a certification sticker for the wash station to reward households for their efforts to maintain behavioural practice.	1 week post Household Visit 1	30 to 45 min
First Rainy Season Reinforcement Event	10 neighbouring households	2 Activators 1 Health Volunteer	Overcome seasonal barriers to behaviour practise. Reinforce the intervention’s messages.	Reminder of trachoma transmission routes. Soap use Emo Demo to elicit disgust as a driver. Problem-solving testimonials and discussion, i.e., lack of time and soap. Maintaining wash station functional. Attributing roles in the family (e.g., washing, repairing). Soap giveaway.	Prior to start of the rainy season	45 min to 1 h
Dry Season Reinforcement Event	10 neighbouring households	2 Activators 1 Health Volunteer	Overcome seasonal barriers to behaviour practise. Reinforce the intervention’s messages.	Reminder of trachoma transmission routes. Demonstration of the benefits of washing with soap. Problem-solving testimonials and discussion, i.e., wash station maintenance and lack of water. Water prioritisation activities. Short drama on avoiding procrastination. Attributing roles in the family. Soap giveaway.	Prior to start of the dry season	45 min to 1 h
Second Rainy Season Reinforcement Event	10 neighbouring households	1 Health Volunteer	Check wash station use and maintenance. Reinforce the intervention’s message.	Demonstration of wash station maintenance. Reminder of the importance of soap for face washing and troubleshooting issues with soap availability (i.e., making soapy water). Attributing roles in the family (i.e., a child cannot wash their face as thoroughly as a caregiver can).	Prior to start of the rainy season	30 to 45 min
Final Reinforcement Event	10 neighbouring households	2 Activators 1 Health Volunteer	Overcome seasonal barriers to behaviour practise. Reinforce the intervention’s messages.	Small gender-separated group events: roles and responsibilities regarding hygiene behaviours (role-modelling) and wash station maintenance. Whole-group event: problem-solving through testimonials and discussion (i.e., lack of soap). Short drama on benefits of washing faces with soap. Soap giveaway.	Last event	1 h to 1 h and 30 min

* *Garee*: a sub-village administrative unit. Actors and Activators were personnel specifically hired and trained to deliver the *Faces of Dignity* intervention. Health Volunteers were community-based health providers, locally recruited and trained to deliver the intervention.

## Data Availability

The raw data from Focus Group Discussions and Interviews conducted during intervention development that support the conclusions of this article will be made available by the authors on request.

## Supplementary Materials

The following supporting information can be downloaded at https://www.mdpi.com/article/10.3390/bs15030355/s1, Intervention Manuals.
