# Supplementary material for: Translating Formative Research into Intervention Content: Experiences with Face Washing for Trachoma Control in Rural Ethiopia
Source: Behav Sci (Basel). 2025 Mar 13;15(3):355. doi: 10.3390/bs15030355 (PMC11939790; doi:10.3390/bs15030355)
Supplement: Supplementary file 1 [file behavsci-15-00355-s001.zip › PDF files/0_FULL MANUAL_Faces of Dignity Campaign Manual_Paper.pdf]

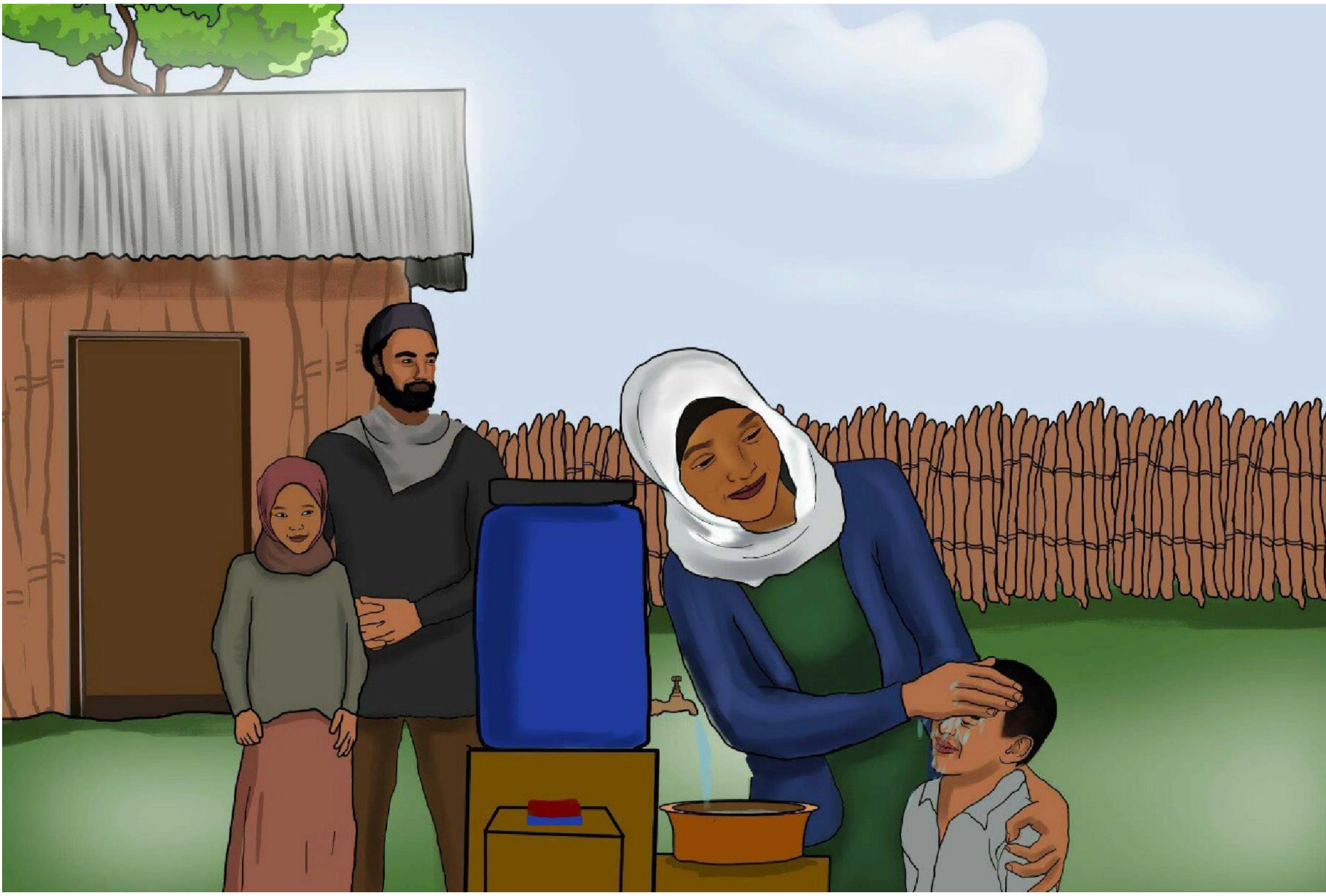

FACES OF DIGNITY CAMPAIGN

INTERVENTION MANUALS

Purpose of the Manuals

This first manual gives an overview of the *Faces of Dignity* intervention delivered during the Wellcome Trust-funded Stronger-SAFE trial to improve trachoma elimination efforts in rural Oromia, Ethiopia. The intervention seeks to improve the frequency and quality of face washing with soap among all household members, but particularly pre-school age children (1 to 6 years old). Male and female heads of households with pre-school age children are the key target population of this intervention. The intervention will be delivered to about 2500 households, 1650 of which will receive an intensive version of the intervention.

This manual provides an overview of the content and materials used in the intervention and is not intended as a guide for intervention delivery. A more detailed account of the conduct of each activity can be found in the Implementation Guide for each event.

Intervention Overview

"Intensive Intervention"

The *Faces of Dignity* Campaign is a multi-level intervention comprising a large group event and a series of small group events and individual household visits.

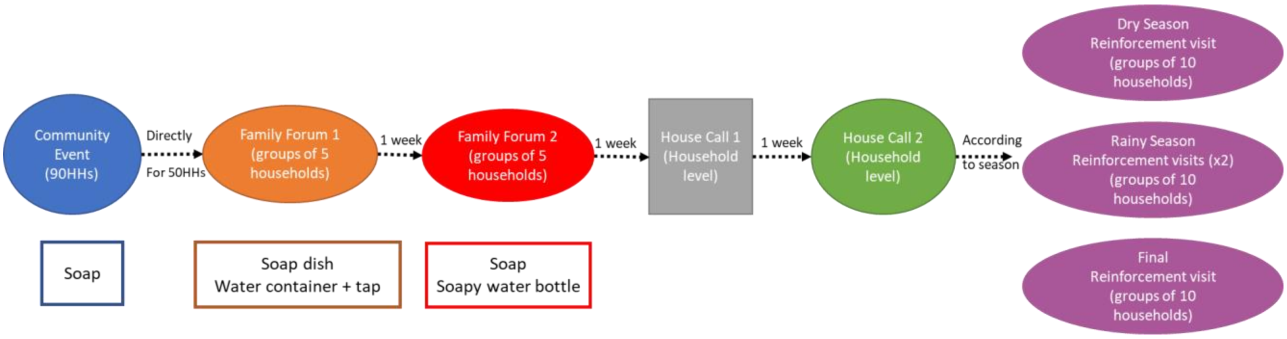

"Basic Intervention"

As the intervention is to be delivered in the context of the Stronger-SAFE trial, and the clusters are quite large, a basic version of the intervention will be delivered to the outer portion of each cluster. This version of the intervention will comprise the Community Event + Provision of a wash station, 2 soaps, a soap dish and a wash station flyer.

If we view a cluster as a fried egg, the structure of the intervention looks like this:

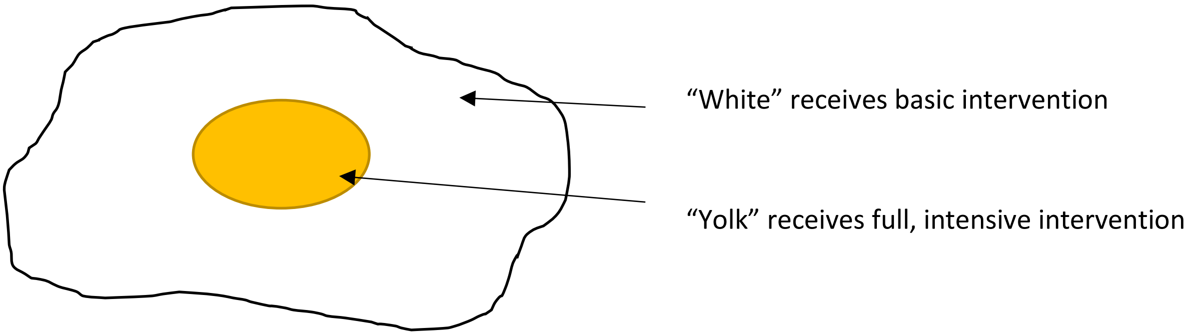

Personnel and Training

The intervention will be delivered in each cluster by a team of facilitators composed of 2 trained Actors, 1 trained Activator, and 2 trained Health Volunteers (HVs). Other individuals required to support intervention activities are described in the content for each specific event. Involving both trained Implementers and local HVs intends to ensure intervention fidelity in the trial context whilst also providing a scalable and replicable model to improve intervention reach and uptake in each cluster.

Table. Overview of the *Faces of Dignity* Campaign in each cluster

Event	Facilitator	Purpose	Target Audience	Timing of event
**Community Event** (large-scale event)	2x trained Actors; Involvement of 2x Influential Leaders; supporting personnel (HVs, Activators, etc.)	Raise awareness and credibility of the campaign among the community; Create buy-in; Begin to build wash-related knowledge and motivation	All household members living in the intervention cluster ('white' + 'yolk'); community leaders	Kick-starts the intervention in each cluster.
**Family Forum 1** (group event with 5xHHs)	1x trained Activator + 1x trained HV	Build wash-related knowledge, skills, and motivation; Empower households to construct wash stations to aid habit formation	All household members living within the 'yolk' of an intervention cluster	In the week following the community event
**Family Forum 2** (group event with 5xHHs)	1x trained Activator + 1x trained HV	Continue to build wash-related skills and motivation; Overcome early barriers related to wash station construction and use; Emphasise need to wash faces with soap three times a day now that everyone has a functioning wash station	All household members living within the 'yolk' of an intervention cluster	1 week after the previous forum (precise timing is dependent on logistics)
**House Call 1** (individual HH visits)	1x trained HV	Reinforce target messages; Provide encouragement to motivate continued action; Identify and overcome specific barriers to ongoing action, including those related to wash station construction and maintenance	All household members living within the 'yolk' of an intervention cluster	1 week after the previous forum (precise timing is dependent on logistics)
**House Call 2** (individual HH visits)	1x trained Activator	Provide further opportunity to reinforce target messages, develop skills and trouble-shoot specific barriers preventing optimal practice of face washing	All household members living within the 'yolk' of an intervention cluster	2 weeks after Family Forum 2 (precise timing is dependent on logistics)
**Reinforcement Events** (group event with 10xHHs)	2x trained Activators (+ 1x HV)	Reinforce initial messages; overcome known seasonal barriers	All adults living within the 'yolk' of an intervention cluster	At start of the rainy season, start of the dry season and final reinforcement

Considerations associated with COVID-19

The content and delivery of the intervention have been revised to ensure all standard precautionary COVID-19 preventative measures are followed and implemented routinely by all individuals involved in the *Faces of Dignity* Campaign. Safety of both the community and *Faces of Dignity* intervention facilitators is paramount. All intervention activities will take place outside.

The main precautionary measures taken include minimisation of unnecessary contact between any individuals (including between implementers), implementation of physical distancing (at least 2 metres between all individuals from different households) in all settings, use of facemasks in public places and appropriate disposal of those masks, handwashing with water and soap for at least 20 seconds or use of at least a 60% alcohol based hand sanitizer, and regular surface cleaning with detergent.

Detailed procedures to be observed by implementation staff (Actors, Activators and HVs) are described in each event manual. Due to the fast-changing nature of the pandemic, the latest national and local guidance on COVID-19 prevention will be followed at all times.

List of all Collaterals for facilitation

Collateral	Corresponding Event
– Puppet of the *Faces of Dignity* Drama	Community Event | FF1 | FF2 | HC2
– Community Banner	Community Event
– *Faces of Dignity* Banner	Community Event
– Flipchart for Family Forum 1 and Family Forum 2	FF1 | FF2
– Trachoma Transmission Routes – Magnetic board	FF1
– Trachoma Transmission Routes – Diagram	FF1 (follow-up visits)
– Face Wipe Emo-Demo – Washing protocol sheets	FF1
– “Dignified Day” Flashcards	FF2 | REs
– Flipchart for Reinforcement Events	REs

List of all Giveaways for participants

NB. Households in the ‘yolk’ only receive the giveaways given Before and After the Community Event, i.e. an invitation, a branded wash station, a soap dish, 2 soaps, and a wash station flyer.

Giveaway	Corresponding Event
– Invitation for Community Event	Before Community Event
– Branded wash station	After Community Event
– Soap dish	After Community Event
– Soaps (6 in total)	After Community Event | FF2 | REs
– Wash station flyer	After Community Event
– Line drawing of the puppet and wax crayons	FF1 | FF2 | REs
– Poster “Do you ensure your family’s Days are Dignified?”	FF1
– Soapy water bottle	FF2 | REs
– Dangler	HC1
– Certification sticker	HC2

Community Event

NB. Refer to Community Event Manual for the full details of the below activities.

**Purpose**	This event is designed to raise awareness and credibility of the *Faces of Dignity* campaign among the community, to create buy in (especially from men) and to begin to build washing-related knowledge and motivation.
**Responsible parties**	2 trained Actors
**Supporting individuals**	2 trained Health Volunteers (HVs); community leaders; identified "Influential Role Models"; other volunteers if needed (to be adjusted during the pilot-testing of the intervention)
**Participants**	All household members living in the intervention cluster ('white' + 'yolk'), community leaders
**Location**	Health post, *garee* office or kebele office compound (selected in advance)
**Duration**	1 hour

Preparation

- Community sensitisation
- Invitation distribution by Health Volunteers
- Set up on the day of the event

Activities

- Activity 1: Megaphone Announcement & Music Playing
- Activity 2: Influential Role Model Introduction
- Activity 3: *Faces of Dignity* Drama
- Activity 4: *Faces of Dignity* Pledge
- Activity 5: *Faces of Dignity* Banner & Record Testimonials
- Following the Community Event: Distribution of Campaign materials

Collaterals used during the Community Event

- Puppet of the *Faces of Dignity* Drama
- Community Banner
- *Faces of Dignity* Banner

Giveaways received by participants

Before the Community Event

- 1 invitation for the Community Event per household

After the Community Event

- 1 branded wash station per household
- 1 soap dish per household
- 2 body soaps per household
- 1 wash station flyer per household

FAMILY FORUM 1

NB. Refer to Family Forum 1 Manual for the full details of the below activities.

**Purpose**	This event is designed to build washing related knowledge, skills, and motivation, and empower households to construct wash stations to aid habit formation.
**Responsible parties**	1 trained Activator + 1 trained Health Volunteer (2 HVs will assist the Activator in each cluster to spread the workload)
**Participants**	All household members living within the 'yolk' of an intervention cluster who attended the Community Event – Split into groups of 5 households.
**Location**	A HH compound (selected in advance when HHs are informed the time and date for the forum)
**Duration**	1h30

Preparation

- Recruitment of households by HVs
- Set up on the day of the event

Activities

- Activity 1: Introduction
- Activity 2: Trachoma Transmission Routes
- Activity 3: Face wipe Emo-Demo
- Activity 4: Wash stations – Constructing a stand and wash station use
- Activity 5: Testimonials
- Activity 6: Dignified Day Pledge
- Activity 7: Conclusion
- <u>Following Family Forum 1:</u> Individual follow-up visits with any non-attending household

Collaterals used during the Family Forum 1

- Puppet of the *Faces of Dignity* Drama
- Flipchart for Family Forum 1 and Family Forum 2
- Trachoma Transmission Routes – Magnetic board
- Trachoma Transmission Routes – Diagram (for follow-up visits only)
- Face Wipe Emo-Demo – Washing protocol sheets

Giveaways received by participants

- 1 line drawing of the puppet per child
- 1 poster "Do you ensure your family's Days are Dignified?" per household

FAMILY FORUM 2

NB. Refer to Family Forum 2 Manual for the full details of the below activities.

**Purpose**	Continue to build skills and motivation to practice face washing. Overcome early barriers related to wash station construction and use. Emphasize the need to wash faces with soap three times a day now that everyone has a functioning wash station.
**Responsible Parties**	1 trained Activator + 1 trained Health Volunteer (2 HVs will assist the Activator in each cluster to spread the work load)
**Participants**	All household members living within the 'yolk' of an intervention cluster who attended Family Forum 1 – Split into groups of 5 households.
**Location**	A HH compound (selected in advance when HHs are informed the time and date for the forum)
**Duration**	1h30

Preparation

- Recruitment of households by HVs
- Set up on the day of the event

Activities

- Activity 1: Introduction
- Activity 2: Live Testimonials
- Activity 3: Wash-Along
- Activity 4: "A Dignified Day"
- Activity 5: Barriers to Facewashing & Solutions
- Activity 6: Soap Giveaway
- Activity 7: Conclusion
- <u>Following Family Forum 2:</u> Individual follow-up visits with any non-attending household

Collaterals used during the Family Forum 2

- Puppet of the *Faces of Dignity* Drama
- Flipchart for Family Forum 1 and Family Forum 2
- "Dignified Day" Flashcards

Giveaways received by participants

- 1 soap per household
- 1 line drawing of the puppet and 1 wax crayon per child
- 1 soapy water bottle per household

HOUSE CALL 1

NB. Refer to House Call 1 Manual for the full details of the below activities and Checklist.

**Purpose**	To provide ongoing support to motivate families to wash faces thoroughly with soap x3 a day.
**Responsible parties**	1 trained Health Volunteer (HV)
**Participants**	All members of a household present at the time of this unannounced visit. Households living within the 'yolk' of an intervention cluster who attended Family Forum 2.
**Location**	Each participant's home
**Duration**	20 to 30 mins

Preparation

- Provide HV with materials required and refresher training for House Call 1

Activities

- Activity 1: Introduction
- Activity 2: Facial cleanliness assessment and Face Washing
- Activity 3: Wash station review
- Activity 4: "A Dignified Day" poster review
- Activity 5: Dangler giveaway
- Activity 6: Conclusion

Collaterals used during the House Call 1

No collateral used.

Giveaways received by participants

- 1 dangler per household

House Call 2

NB. Refer to House Call 2 Manual and House Call 2 Checklist for the full details of the below activities.

**Purpose**	To provide ongoing support to motivate families to wash faces with soap 3x a day throughout the year. Provide support to help families maintain their wash stations.
**Responsible parties**	1 trained Activator
**Participants**	All members of a household present at the time of this unannounced visit. Households living within the 'yolk' of an intervention cluster who received House Call 1.
**Location**	Each participant's home
**Duration**	30 to 45 mins

Preparation

No specific preparation required except preparation of materials

Activities

- Activity 1: Introduction
- Activity 2: Video Demo
- Activity 3: Wash station review
- Activity 4: Wash Station Maintenance
- Activity 5: Poster and Dangler Review
- Activity 6: Wash Station Certification
- Activity 7: Conclusion
- Following House Call 2: Community leaders will be gathered at the location where the Dignity Banner is up to publicly declare the community a Dignified Community. The Dignity Banner will be amended at the occasion. Consecutively, community leaders will be rewarded for their contribution to the intervention.

Collaterals used during the House Call 2

- Puppet of the *Faces of Dignity* Drama

Giveaways received by participants

- 1 certification sticker per household

REINFORCEMENT EVENTS

NB. Refer to Reinforcement Events Manual for the full details of the below activities.

**Purpose**	Reinforce key messages and narratives of the Campaign (face washing with soap x3 a day, with an emphasis on preschool children) and overcome seasonal barriers to face washing behaviour.
**Responsible parties**	2 trained Activators (assisted by Health Volunteers and community leaders when necessary)
**Participants**	All adults living within the 'yolk' of an intervention cluster who received the *Faces of Dignity* Campaign – 10 household groupings
**Location**	A HH compound (selected in advance when HHs are informed the time and date for the forum) or a public space
**Duration**	45 mins to 1h30

Preparation

- Recruitment of households by HVs
- Set up on the day of the event

First Rainy Season Reinforcement Event

Activities

- Activity 1: Introduction & Signposting to the *Faces of Dignity*
- Activity 2: Face wipe Emo-Demo – Short version
- Activity 3: Problem Identification & Testimonials
- Activity 4: Problem-solving – Lack of Time
- Activity 5: Problem-solving – Forgetfulness
- Activity 6: Problem-solving – Wash station use and Maintenance
- Activity 7: Problem-solving – Lack of Water
- Activity 8: Problem-solving – Lack of Soap
- Activity 9: Soap Giveaway
- Activity 10: Conclusion

Collaterals used during the First Rainy Season Reinforcement Event

- Flipchart for Reinforcement Events
- Face Wipe Emo-Demo – Washing protocol sheets

Giveaways received by participants

- 1 soap per household

Rainy Season Reinforcement Event

Activities

- Activity 1: Introduction & Signposting to the *Faces of Dignity*
- Activity 2: Demonstration of the Benefits of Washing with Soap
- Activity 3: Problem Identification & Testimonials
- Activity 4: Problem-solving – Wash station use and Maintenance & Discussion on Perceptions of Roles
- Activity 5: Problem-solving – Lack of Water
- Activity 6: Problem-solving – Lack of Soap
- Activity 7: Problem-solving – Procrastination: Short Drama
- Activity 8: Soap Giveaway
- Activity 9: Conclusion

Collaterals used during the Dry Season Reinforcement Event

- Flipchart for Reinforcement Events

Giveaways received by participants

- 1 soap per household

Second Rainy Season Reinforcement Event

Activities – Individual Household's Event

- Activity 1: Wash station checks

Activities – Group Event

- Activity 1: Wash station Maintenance
- Activity 2: Demonstration of the Benefits of Soap
- Activity 3: Problem-Solving – Lack of Soap
- Activity 4: Wash-Along
- Activity 5: Conclusion

Final Reinforcement Event

Activities – Men's Event

- Activity 1: Introduction & Campaign Reminder
- Activity 2: Role as a Role Model
- Activity 3: Problem-solving – Wash station maintenance
- Activity 4: Problem-solving – Lack of Soap

Activities – Women's Event

- Activity 1: Introduction & Campaign Reminder
- Activity 2: Role as a Role Model
- Activity 3: Soap!

Activities – Whole Family Event

- Activity 1: Roles & Responsibilities
- Activity 2: Success Testimonials
- Activity 3: Get these Children's Faces Washed (with soap)!
- Activity 4: Recap

- Activity 5: Soap giveaway & Soapy water bottles
- Activity 6: Conclusion

Collaterals used during the Final Reinforcement Event

- Flipchart for Reinforcement Events

Giveaways received by participants

- 1 soap per household
- 1 soapy water bottle per household
- 1 line drawing of the puppet and 1 wax crayon per child

EVENT 1 – COMMUNITY EVENT
ACTIVATOR MANUAL

**Purpose**	This event is designed to raise awareness and credibility of the *Faces of Dignity* campaign among the community, to create buy in (especially from men) and to begin to build washing-related knowledge and motivation.
**Responsible parties**	3 trained Actors
**Supporting individuals**	2 trained Health Volunteers (HVs); community leaders; identified "Influential Role Models"; other volunteers if needed (to be adjusted during the pilot-testing of the intervention)
**Participants**	All household members living in the intervention cluster ('white' + 'yolk'), community leaders
**Location**	Health post, garee office or kebele office compound (selected in advance)
**Duration**	1 hour
**Timing**	Mondays, Wednesdays, Sundays (avoiding local market days) – Morning from 9am to 1pm

Preparation

During the week before the event

Meet community leaders at cluster level (zone / garee leaders, clan / religious leaders, HVs / HEWs):

- If possible, hold the meeting in an outdoor location. Ensure you are wearing a facemask during the entire meeting. Wash your hands with soap or alcohol-based sanitizer before and after the meeting. Ensure you are safely disposing your facemasks in a sealable plastic bag.
- Remind them about the Stronger-SAFE trial and their community's allocation to the intervention arm (or communicate this if it has not already been discussed). Remind them which interventions they will receive. **Refer to Appendix A** for guidance on Community Sensitisation.
- Introduce the *Faces of Dignity* campaign and encourage them to support it.
- Brief them on the content of the Community Event.
- Identify a suitable and accessible location, date and time for the Community Event.
- Discuss who should assist with the set up and running of the Community Event.
- Identify prominent location to display the community's *Faces of Dignity* banner.
- Request the presence of all community leaders at the Community Event.
- Finalise selection of leaders to speak at the Community Event in the role of "Influential Role Models" – ensure Influential Role Models can read if anything written has to be read at loud. **Refer to Appendix B**.
- **Give wash stations, soaps, soap dishes and Campaign hi-vis jacket to Health Volunteers. Give wash stations, soaps, and soap dishes to Influential Role Models***. Explain why it is important that they model and start practicing the desired behaviour.
- Assess willingness / availability of Influential Role Models to intervene at the Community Event and voice-record Testimonials at the end of the Community Event. Purpose of Testimonials can be found in **Appendix C**.
- Arrange for storage of Wash Stations (soap dishes, soaps and wash station flyer) for the cluster (~90) in a secure location until they are distributed after the Community Event. **Refer to Appendix D** for guidance on how to organise the wash station and giveaways distribution.

**NB: This may be amended. Likely to give wash stations, soaps, and soap dishes to the Kebele leader, Zone Leader and HEW as well. "Influential Role Models" might also receive campaign caps.*

Day before the event*

- Provide two HVs with the census list and invitations for the Community Event.
- Instruct HVs to go house-to-house to distribute 1 invitation to each family. If the primary caregiver or the household head are not at home, HVs can distribute the invitation to any child over the age of 14 who is a household member. HVs should ask this child to inform his/her parents about the event.
- Brief HVs on the invitation pitch and what to do if they identify anyone who needs support getting to the Community Event. As the Community Event might be the only event where household heads come, HVs should emphasize that both female primary caregiver and male household head are invited to come to the event with their children.

> **"We will be holding a drama tomorrow at [chosen place] at [chosen time] where you will learn how to make sure you and your family have *Faces of Dignity*. You will be informed where and when to get a gift at the end of the drama. Make sure you come on time so you do not miss the music and introduction by a surprise and special guest.**
>
> **Your whole family is invited. We can arrange for assistance if any members of your family will find it difficult to attend the event, for example because they are unable to walk to [chosen place] or sit comfortably."**

**NB: If it is logistically difficult to visit the cluster again before the event, instruction for recruitment should be given during the Sensitisation meeting outlined above. HVs should be requested to deliver invitations the day before the Community Event whenever possible to increase attendance. HVs will be called the day before the event to remind them to distribute invitations if they are given the invitations early.*

Setting

The sketch below illustrates the ideal setting for the community event. Activators are free to amend the set-up according to each health post/garee office/kebele office. Any setting should respect the following criteria as much as possible:

- Participants should be able to SEE and HEAR the full event easily no matter where they are sitting (test the microphones).
- Elderly or disabled participants should be directed to sit on chairs or stools at the back or sides of the stage (wherever they will not obstruct the view of anyone else).
- Participants should be comfortable i.e. they should be sat in the shade / should be able to view the stage without being blinded by the sun.
- Materials for the drama should be organised and located out of sight of the audience as much as possible.
- Participants should sit with their household members and observe a physical distance of at least 2m with other households, where possible.

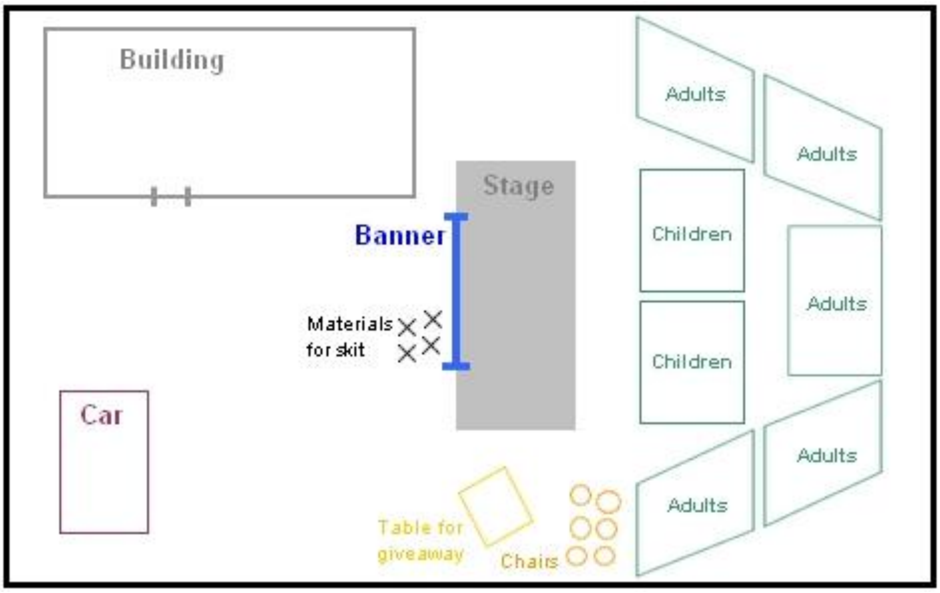

Materials

General

- COVID-19 preventive materials: cleaning solution in sprays (x4), facemasks (all support responsible parties, supporting individuals, and people needed transportation arrangements) sealable plastic bag to dispose masks
- Megaphone
- Logo banner
- Microphones + Speakers + Power bank
- Plastic stools for HEW/HV/Fieldworker/Influential speakers/any participants who cannot sit on the ground (borrowed from Shashamane office or in the cluster)
- A bell (used in the drama)

Drama

- Clothing for actors
  - Actress: dress, scarf, jewels, shoes
  - Actor: scarf, shoes
  - Puppet actress or actor: neutral / black clothing)
- Caltu's puppet
- Plastic stools (x2)
- Coffee tray + jebena + 6 coffee cups
- Jug + collector + soap dish + soap
- Jerry can of 20L filled with water
- Wash station

***Faces of Dignity* Pledge**

- Wash station + soap dish + soap (reuse from the drama)
- Palm cards for actors
- *Faces of Dignity* banner
- Permanent markers for outlining hands on the banner
- Rope/Nail to put up the banner at the end of the event

Testimonials recording

- A phone or device with a microphone

Set up

Day of the community event

- Do you have all materials required for all activities?
- Is equipment working?

Megaphone, microphones and speakers are charged/have full batteries.
Quality of sound has been checked from all angles.

- Have chairs / stools been set out?
- Are the 'Influential Role Models' present? Have they been briefed?
- Have the Health Volunteers been briefed on their role?
- Have additional volunteers been identified to help with the set up and running of the event if needed? *NB: the need for this will be assessed during piloting.*

Activities

Activity 1: Megaphone announcement & Music Playing

Purpose

- To remind the population that the community event is about to commence (i.e. to increase attendance).

What to do

In the community

1. The HVs should announce the event by megaphone on foot or using a motorcycle/car, depending on the terrain. The announcement should tell everyone to make their way to the chosen location and explain that the whole family should attend.
2. Upon arrival at the event location, supporting individuals (HVs, activators, etc.) should advice community members to sit with their household members and respect a physical distance of at least 2m with other households.
3. HVs should liaise with community leaders to ensure any community members needing assistance (e.g. people with disability) are supported to get to the event e.g. transportation is provided to and from the event and they are guided to a comfortable seat. Community members who are transported to the event location should be given facemasks before entering the vehicle.

At the event

1. Actors play the Dignity Song on the event speakers as people gather.
2. Actors ensure Community Leaders / Influential Role Models are present and briefed on what to say during the introduction and after the drama.

Activity 2: Influential Role Model Introduction

Purpose

- To increase the credibility of the intervention and increase willingness of participants to be part of the programme.
- To provide role modelling and create the impression that important others in the community are already washing faces with soap 3x a day.
- To trigger the feeling of ownership (of the programme) among the community leaders.

What to do

1. Stop the music and ring a bell to get the crowd's attention.
2. Invite the three chosen Influential Role Models to take the stage with you (the Actors) and the Campaign Health Volunteers. Get everyone's attention. Do not begin until the crowd is quiet.
3. Ask the leader of the chosen Influential Role Models to welcome the community to the event and to introduce you and the HVs dedicated to working on this Campaign.
4. Ask the following question to the three Influential Role Models:

> Dignity: **"Do you value your dignity and why?"** Let the Influential Role Models answer.
> Children's dignity: **"Do you value your children's dignity and why?"** Let the Influential Role Models answer.
> Community's dignity: **"Do you believe that the dignity of everyone in the community, i.e. the community's dignity is important too? Why?"** Let the Influential Role Models answer.

5. Thank them for caring so much about their community and tell them that you are here today to tell them how they can ensure they all have *Faces of Dignity*. Invite the Leaders to leave the stage so you can begin.

Activity 3: *Faces of Dignity* Drama

Purpose

- Improve understanding that trachoma is spread by flies and on hands and that washing faces with soap can remove discharge and prevent trachoma transmission
- Give an overview of the key face washing messages of the campaign in a humorous and memorable way, including the importance of soap for face washing
- Cause people to revalue face washing and link the behaviour to the *Attract*, *Affiliation* and *Status* motives (the latter two via dignity)
- Create the impression that family members and neighbours expect you to wash your face and the faces (and hands) of your children 3x a day with soap

NB. **Refer to Appendix E** – *Script for the Faces of Dignity Drama.*

Activity 4: *Faces of Dignity* Pledge

Purpose

- Perceive soap as important to use each time faces are washed
- Perceive effectively washing face at least 3x a day as important, all year around
- Accept responsibility for hygiene of young children
- Perceive an expectation from neighbours & husbands to maintain clean faces of self & family
- Perceive an expectation from parents to maintain clean faces

What to do

Influential Role Models' testimonials and face washing demonstration

1. Invite the Influential Role Models to come back on stage and bring forward the wash station, soap dish and soap used during the drama.
2. Ask the Influential Role Models if they would like their family to have *Faces of Dignity* and why.
    a. Each Influential Role Model should quickly tell one's own story having and using a wash station and making sure soap is always available for body washing. **Refer to Appendix C** for guidance on topics which can be covered by the Influential Role Models.
    b. Ask each Influential Role Model to come forward and demonstrate how to wash their hands and faces with soap using the station. They should be mindful of closing the tap while rubbing their faces and hands.
    c. Thanks the Influential Role Models for being *Faces of Dignity* in their community.
3. Ask participants to raise their hands if they too, like Caltu's family and the Influential Role Models, would like to have *Faces of Dignity*.

Faces of Dignity banner

1. Uncurl the *Faces of Dignity* banner and hold it at each end.
   Say that we can do some activities to help us all ensure our families have *Faces of Dignity*. Explain that each family committing to have *Faces of Dignity* will also contribute to enhancing their community's dignity. All faces should be dignified for the community to be dignified.
   Invite the leader of the Influential Role Models to read out the activities on the banner.

I wash my face and hands with soap when I wake up, before lunch and before my evening meal.

I help my pre-school children wash their faces and hands with soap when they wake up, before lunch and before their evening meal.

I help my family maintain their *Faces of Dignity* and contribute to enhancing my community's dignity.

2. Ask participants to raise their hands if they agree that someone doing these activities would have a *Face of Dignity* and contribute to enhancing the dignity of their community
3. Ask the Influential Role Models if they intend to ensure their family does these activities to have *Faces of Dignity* and contribute to enhancing their community's dignity
4. Ask the crowd if they want to do these activities to have *Faces of Dignity*.
5. Ask the crowd if they want to become *Faces of Dignity* to contribute to enhancing their community's dignity.

Collective Pledge

1. Tell the crowd that we are going to pledge to do these activities together as a community.
2. Ask the leader of the Influential Role Models to <u>read out the pledge</u> slowly and ask the audience to <u>stand up</u> with family members and <u>repeat after him/her</u>:

I pledge to maintain my *Face of Dignity*, ensure my Family all have *Faces of Dignity*, and contribute to enhancing my community's dignity.

3. Congratulate all participants on their pledge. Inform them that they will be supported and monitored on their progress to become *Faces of Dignity* in the coming weeks. In a few weeks' time, if all members of the community have *Faces of Dignity*, the Influential Role Models and community leaders will be able to declare their community to be a Dignified Community and amend the banner.
4. Conclude the event by informing the community that:
    a. In each household, <u>**the household head or the female primary caregiver can go to [the chosen place] on the [chosen days]**</u> to pick up their wash station, soap dish and soaps. **Refer to <u>Appendix D</u>** for guidance on how to organise the wash station distribution.
    NB. Exact logistics on how and when the wash stations will be given to the community will be decided on a cluster-to-cluster basis and might be amended during pilot-testing of the intervention.
    b. Say that some ongoing activities and small group meetings will continue in the community, but <u>it will not be possible</u> to visit all households.

Signing the *Faces of Dignity* banner

1. <u>Stretch the banner out</u> on the ground and put out the permanent markers.
2. Invite the Influential Role Models to come forward and "sign" the banner by <u>outlining their hand with a marker</u>.
3. Invite each household to send <u>one</u> child (accompanied by the caregiver if possible) to "sign" the banner. This activity is supervised by one Actor, one Health Volunteer and one Influential Leader.
    a. Actor and HV should wear a facemask during this activity as they will be in close contact with children and caregivers.
    b. Between each child, the Actor or the HV should disinfect the marker using a cleaning spray.

c. After the child has signed the banner, the accompanying adult should be directed to Giveaway ‘stand’ to receive their 2 soaps.

4. Play the Dignity Song on the loud speakers (if it does not interfere with the activities).

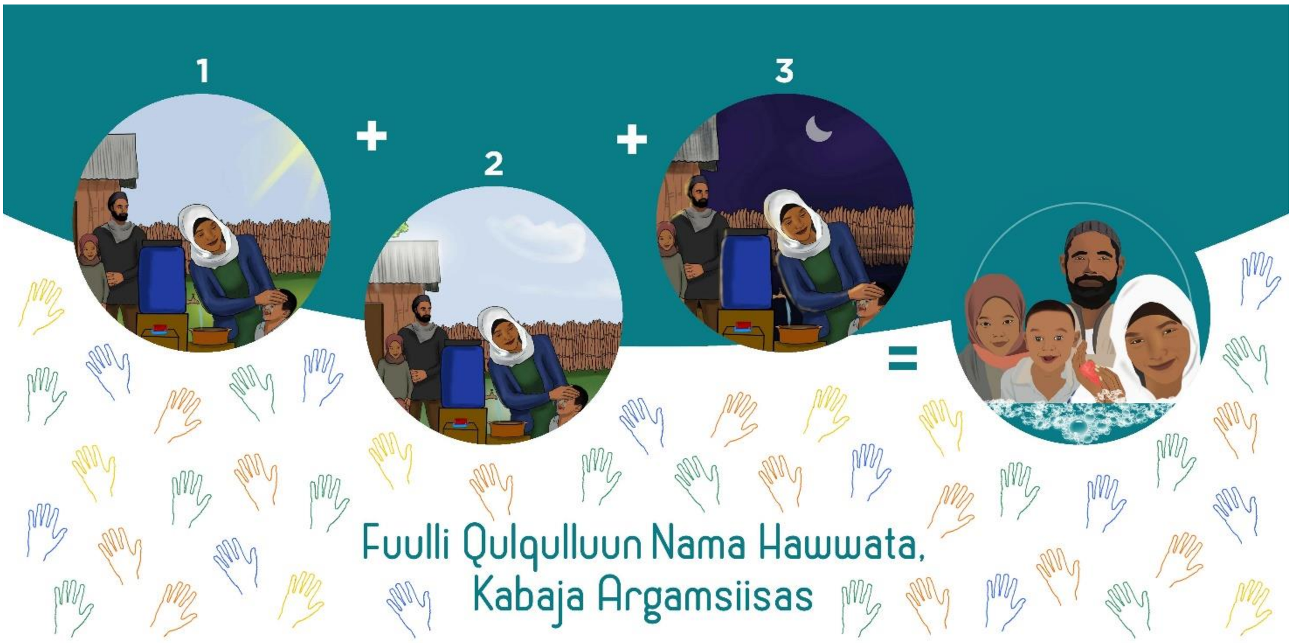

ACTIVITY 5: *FACES OF DIGNITY* BANNER & RECORD TESTIMONIALS

Purpose

- Give the community a permanent reminder of the key messages of the campaign and their pledge
- Create the impression that everyone in the community washes faces with soap x3 a day

What to do

1. Put the *Faces of Dignity* banner up at the designated location chosen by the Community Leaders during sensitisation, with the help of the HVs and any volunteers.
2. Before they leave, voice-record testimonials with Influential Role Models using a smartphone or a voice-recorded. They should basically repeat and elaborate on what they said after the drama. **Refer to Appendix C** for guidance on topics which can be covered by the Influential Role Models. *NB. To be adapted during pilot-testing to see whether it is feasible. Might be done with the Lead Influential Role Model only.*

End of Community Event.

Appendices

Appendix A. "Guidance on Community Sensitisation for the *Faces of Dignity* Campaign"

Purpose

- Remind or inform community leaders about their allocation to an intervention arm.
- Remind or inform community leaders about the interventions their community will receive.
- Answer any question related to the trial or intervention design.

Responsible parties

- Berhan Supervisors are responsible for ensuring the Community Sensitisation is appropriately done in each cluster prior to the delivery of the Campaign. In case Berhan Supervisors cannot attend the meeting, Berhan Actors or Activators should cover the Community Sensitisation content.

Procedures

NB. If possible, hold the meeting in an outdoor location. Ensure you are wearing a facemask during the entire meeting. Wash your hands with soap or alcohol-based sanitizer before and after the meeting. Ensure you are safely disposing your facemasks in a sealable plastic bag.

1. Ask the community leaders if they have already been informed about their allocation to an intervention arm.
    a. If they have already been informed (e.g. at start of MDA), ask them to summarize what interventions they are expecting to receive and correct any misunderstandings. Describe intervention packages the community will receive according to their allocation arm (<u>details provided in the boxes below</u>).
2. Ask the community leaders if they have any questions on their allocation to the intervention arm or on the interventions their community will receive.
3. Introduce the *Faces of Dignity* Campaign:

- Intensive and inter-personal intervention targeting around 90 (to be adapted to each cluster size) households in some garees of the zone.
- Communication is at 3 levels: community (1 meeting with a community drama), small group (2 forums with groups of 5HHs) and household (2 household visits). Only 50 households will receive the small groups and household visits.
- Campaign (i.e. 5 visits) will take place over 1 month and be followed by further "reinforcement" group meetings will take place periodically throughout the year, timed to coincide with key seasonal barriers to face washing.
- The intervention involves provision of the following hardware and props: branded water containers with faucets, branded soap dishes, bars of body soap.

4. Introduce the Community Event:

- Large scale event delivered by 3 trained actors and involving Influential Role Models from the community as well as Health Volunteers.
- **Purpose:** raise awareness and credibility of the Campaign among the community and begin to build wash-related knowledge and motivation.
- About 90 households (to be adapted to each cluster size) will be invited as well as all community leaders (including those present at this meeting)

5. Ask the community leaders if they have any questions about the Campaign.

Allocation arm: "**Standard** A intervention and **enhanced** F&E interventions"

1. Remind community leaders about trachoma, i.e. an eye disease caused by a germ called *Chlamydia* that many people catch in Ethiopia. It can cause people to go blind.
2. Remind them about the Stronger-SAFE trial, i.e. aiming to study the best ways to remove trachoma from your community.
3. Explicitly announce to the community leaders which intervention arm their community has been allocated to, i.e. your community will receive the current antibiotic treatment (single-dose treatment) and some households will receive hygiene messages or new fly control interventions (which households receive these tools will be decided randomly (like tossing a coin)).
4. Quickly remind them about the trial's demands on the participants:
    a. Test for trachoma (eye and face examinations), up to eight times over the next three years
    b. Fly control measures, including fly trap and anti-fly headwear for children
    c. Some households will receive hardware to aid hygiene behaviour (not everyone in the zone)
    d. A WASH intervention, covered in more details after, including a community event, two small-group visits, two household visits and additional reinforcement visits periodically
    e. Voluntary participation to extra studies on fly control or hygiene interventions

Allocation arm: "**Enhanced** A intervention and **enhanced** F&E interventions"

1. Remind community leaders about trachoma, i.e. an eye disease caused by a germ called *Chlamydia* that many people catch in Ethiopia. It can cause people to go blind.
2. Remind community leaders about the Stronger-SAFE trial, i.e. aiming to study the best ways to remove trachoma from your community.
3. Explicitly announce to the community leaders which intervention arm their community has been allocated to, i.e. your community will be offered two doses of antibiotic (given 2 weeks apart) and some households will receive hygiene messages or new fly control interventions (which households receive these tools will be decided randomly (like tossing a coin)).
4. Quickly remind about the trial's demands on the participants:
    a. Test for trachoma (eye and face examinations), up to eight times over the next three years
    b. Fly control measures, including fly trap and anti-fly headwear for children
    c. Some households will receive hardware to aid hygiene behaviour (not everyone in the zone)
    d. A WASH intervention, covered in more details after, including a community event, two small-group visits, two household visits and additional reinforcement visits periodically
    e. Voluntary participation to extra studies on fly control or hygiene interventions

Appendix B. "Identifying Influential Role Models"

Purpose of the Influential Role Models

- Provide role modelling of the target behaviour within their local area.
- Introduce the *Faces of Dignity* Campaign during the Community Event.
- Record testimonials which will form part of the intervention content in each local area.

Specific roles

- The involvement of Influential Role Models in the *Faces of Dignity* Campaign will be introduced to the community formally during the Community Event. The lead Influential Role Model will introduce the programme and Actors and explain why it is important.
- They will be asked to spend few hours providing a voice-recorded testimonial about using the wash station and practicing face washing with soap 3x a day, including how they have overcome any obstacles associated with these behaviours. The Testimonials will be used during Family Forum 1, House Call 2 and the Reinforcement events (if possible).
  NB. This might be revised after pilot-testing in the field according to feasibility.

Selection

- Three Influential Role Models will be selected to voice-record a testimonial for Family Forum 1 (3 testimonials). The "Lead" Influential Role Model will be asked to provide an additional testimonial for House Call 2 to congratulate participants on their efforts. Voice-recording will take place at the end of the Community Event.
- Influential Role Models will be selected based on the following:
  1. Influential and respected community member suggested by HEWs, e.g. clan or religious leader, or by community members themselves in baseline surveys.
  2. Self-reported capacity and interest to undertake the required work (participation in the community event and recording of testimonials).
  3. Positive attitude to face washing and hand hygiene.
  4. Confirmation that they are influential and viewed positively in the community by key community members e.g. teachers and HEWs/HVs.
  5. Phone ownership.

Timing of selection

- Influential Role Models will be identified during early contact with the cluster (ideally during baseline data collection) by the Stronger-SAFE field team. If that is not possible, they will be identified around sensitization for the *Faces of Dignity* Campaign by the Stronger-SAFE team in conjunction with Berhan.

Training

- No specific training is foreseen to engage the Influential Role Models. They will be briefed on their roles and what is expected from them for the Community Event and testimonials at the sensitisation meeting, the day before the Community Event and on the day of the event/testimonials recording.

Motivation

Influential Role Models will receive:
- All intervention materials (wash station container, soap dish, soap, etc.)
- A cap with the campaign logo they should wear during the community event and testimonials recording. *NB. Incentives might be revised according to budget and feasibility.*

Appendix C. "Recording Influential Role Model Testimonials"

Purpose of the Testimonials

- To role model use of a wash station to practice face washing with soap 3x a day and suggestions of how to overcome barriers associated with this practice.
- To increase the legitimacy of the *Faces of Dignity* Campaign.

Testimonials to record

- Four testimonials per intervention cluster should be voice-recorded at the end of the Community Event. Each of the Influential Role Models will record one testimonial for the Family Forum 1. The lead Influential Role Model (designed among the three Role Models) will also record the *"Congratulating participants"* for House Call 2.
    a. Family Forum 1: (1) *"Building a wash station"*, (2) *"Maintaining a wash station"*, (3) *"Routine use of a wash station"*.
    b. House Call 2: (4) *"Congratulating participants"*.

  NB. Full content of each testimonial can be found at the end of this guide as well as in the relevant Family Forum and House Call guides.
  Process for recording testimonials (voice recording or video recording) will be finalised during the pilot-testing of the intervention.
- In the event that it is not possible to record all four testimonials in a cluster, testimonials should be recorded in the following order of priority:
    a. *"Congratulating participants"* for House Call 2.
    b. *"Maintaining a wash station"* for Family Forum 1.
    c. *"Routine use of a wash station"* for Family Forum 1.
    d. *"Building a wash station"* for Family Forum 1.

  NB. If the lead Influential Role Model is not available to record any testimonial, the "Congratulating participants" should be recorded with either of the other two Influential Role Models.

Personnel

- Testimonial recording will be supervised by the Stronger-SAFE field team in conjunction with Berhan's trained Actors/Activators.

Timing

- Recording of the Testimonials will take place at the end of the Community Event.

Materials

- A smartphone or a voice-recorder.

Procedures

1. Remind Influential Role Models about the purpose of the Testimonials and what they will be used for in the intervention (videos shown during Family Forum 1 and/or House Call 2).
2. Confirm their willingness to record the testimonials, based on what they said earlier during the Community Event vent.
3. Brief the Influential Role Model about the content of the testimonial you want to record with her/him (e.g. *"Building a washing station"*). Explain that you will ask them questions related to their washing station to which she/he should answer based on their own experience. Use the "model" testimonials below to probe Influential Role Models.
4. Inform the Influential Role Model that before recording the testimonial, you would like to do a practice round.

5. <u>Do a practice round</u> with the Influential Role Model and provide feedback about the content if needed. <u>Repeat Step 5</u> if necessary.
   NB. The Influential Role Model does not have to cover the exact content provided in the "model" testimonials but should cover relevant points and should share their own experience in line with the topic as naturally as possible. Guide them using the "model" testimonial if they have difficulties deciding what to say.
6. <u>Test</u> the sound by recording a brief conversation and playing it back. Delete the recording once you are satisfied with the quality.
7. Tell the Influential Role Model that you will now record the testimonial which will be played to their community.
8. Before starting the recording, <u>check</u> that: the environment is quiet.
9. <u>Voice-record the testimonial</u> using the smartphone / voice-recorder.
10. <u>Playback the testimonial</u> and check the <u>quality</u>. Check the <u>content</u> of the testimonial is aligned with expectations and that the Influential Role Model is happy with the content.
11. <u>Repeat the recording</u> of the testimonial if the quality and content are not in line with expectations.
12. When you are satisfied with the recorded testimonial, thank the Influential Role Model and end the visit.

Content of the Testimonials – "Model" testimonials

For Family Forum 1

- Testimonial: ***"Building a washing station"*** | Interviewee: Male Influential Role Model
  Question: Why did you decide to build a wash station stand?
  Answer: I built a wash station stand for my wash station so my family and I could wash our faces with soap easily so we have *Faces of Dignity* in the community and.
  Question: How did you decide on the location and the height of your station?
  Answer: I built my wash station stand near to the entrance of our house so the wash station is protected from the sun and easily moveable at night, when I bring the wash station container, the soap dish and the soap inside. I built the stand like this so my older children can use it easily, but my younger children, who are supported by my wife or myself to wash, cannot play with the soap and make it dirty, or with the tap and waste the water.
  Question: Was it hard to build a wash station stand?
  Answer: Not at all! *They should explain what they did. E.g.* It was easy and quick to build the stand with some wood I collected. I made a strong structure and then put the container on top of it. I dedicated a baldy to collect the wastewater, and added a dedicated body soap which I put in the soap dish near the container. It was done in less than 2 hours.

- Testimonial: ***"Maintaining a washing station"*** | Interviewees: Influential Role Model with her husband/his wife
  Question: Have you set some roles for taking care of the washing station in your house?
  Answer (male): Yes, we have. *They should explain what they have done. E.g.* At first it was not easy to get used to the station, moving the wash station container, filling up the water, always having a dedicated soap. That's why my wife and I decided to set some roles so we don't forget anything. I take care of the materials, bring the station in and out in the morning and at night. My wife is responsible for water and soap and making sure the station is always secured during the day. For instance, that the children do not play with the tap.
  Question: Is your wash station always staying outside?
  Answer (male): *They should explain what they have done. E.g.* At first, we thought about leaving the washing station outside at all time, but we had nothing good enough to secure it from wild animals or to make sure we do not lose everything overnight. So, our wash station is brought inside our home at night. Because I am often the one waking up

and going outside first in the morning, I am the one responsible for putting the wash station container and the soap and soap dish on the washing station stand every morning. At night, after the evening wash, I am also the one responsible for bringing the wash station container and the soap dish inside the house when I close the door. I never forget to put the station outside in the morning or inside at night because the wash station stand is visible from the door of our house.
Question: How do you make sure water is always available at the station?
Answer (female): *They should explain what they have done. E.g.* I am usually the one responsible to fetch water every morning. Since we have the station, I have dedicated the water of one of our jerry cans to fill the washing station. Like this, that is really easy. I don't have to worry about having enough water for washing 3x a day. Often, there is even some water left inside the washing station container at the end of the day for the day after.
Question: How do you make sure a dedicated body soap is always available at the station?
Answer (female): *They should explain what they have done. E.g.* Each time I notice that the soap is going to run out soon, I ask my husband for money to buy a dedicated soap for face and body washing. He always gives me money or buys the soap himself. When possible, I buy two soaps at the same time to make sure we always have a spare soap. We thought it would be difficult to keep soap outside because we are not used to do this, but it works and helps us remember to use soap when we wash. That is how we are maintaining our washing station to make sure our family has *Faces of Dignity*.

- Testimonial: ***"Routine use of a washing station"*** | Interviewee: Influential Role Model
  Question: How do you remember to wash your family's faces three times a day?
  Answer: In the morning, we used to wash our faces before having the station, so that is easy. But now we have the station, I never forget about using the soap and supporting my younger children so I can wash their eyes and noses thoroughly. I supervise the older ones too. Before lunch, no one has ever forgotten about face washing with soap, as we are also washing our hands before eating at the wash station. We have associated washing hands and washing faces. That is a good reminder. In the evening, we all wash our faces and hands before dinner, our children even remind us about it. In our family, everyone knows about face washing 3x a day with soap, morning, before lunch and before dinner.
  Question: What do you like about using the washing station?
  Answer: The washing station has made our lives so much easier and we have saved a lot of water. Now we have it, it is so much simpler to maintain our family's *Face of Dignity* by washing our children's faces three times a day with soap. With our wash station and soap always nearby, it is so simple. In my family, we have *Faces of Dignity*, do you?"

For House Call 2

- Testimonial: ***"Congratulating participants"*** | Interviewee: Lead Influential Role Model
- "Congratulations for working so hard to become a dignified family and all having *Faces of Dignity*. I personally want to thank you all for being dignified community members. Your community is proud of you as everyone needs to work together to ensure our community all have *Faces of Dignity*. Thanks to all of you, our community is a Dignified Community! The certification sticker which is given to you now acknowledges your efforts over the last month to wash your faces x3 with soap a day to become *Faces of Dignity*. Like me, stick it on your wash station container to always remember your progress. Congratulations again and keep up the good work!"

Appendix D. “Wash station distribution for all households following the Community Event”

Purpose To distribute the wash station container, soap dish, soaps and wash station flyer to all households (‘yolk’ + ‘white’).

Setting Central location in the cluster (e.g. health post, garee office, etc.) – According to the outcome of the discussion with community leaders during the Campaign sensitisation. This should be the place where all the wash stations are stored. Distribution can take over 2 to 3 days according to HVs’ and community leaders’ availability.

Personnel At least 2 people. One or 2 intervention Health Volunteers (HVs) and/or community leaders supervised by a trained Activator from Berhan.

Timing Just after the Community Event for 2 to 3 days according to personnel’s availability.

Materials

- Wash station and giveaways distribution form (based on census)
- 2x pens
- 1 table + 2x plastic stools
- Wash station containers (1 per HH), soap dishes (1 per HH), soaps (2 per HH), wash station flyers (1 per HH) for all households (‘yolk’ + ‘white’)
- Facemasks for HVs and community leaders, alcohol-based sanitizers, cleaning solution in a spray (x1), sealable plastic bag for mask disposal

Preparation

Day before the wash station distribution or Morning before the distribution

- All information about time and venue for the wash station distribution should be given at the Community Event.

Day of the distribution

- HVs put their facemask on and wash their hands with water and soap or alcohol-based sanitizer before and after the distribution.
- HVs set up a table and two plastic stools in the shade at the chosen place.
- HVs check they have enough wash stations, soap dishes, soaps and wash station flyers for all households (‘yolk’ + ‘white’)
- HVs check they have the “wash station and giveaways distribution form” to be completed.

Distribution

1. HVs sit at the distribution point and wait for households to come and pick up their wash stations and giveaways.
2. Each time a household arrives at the collection point, the HVs find the name of the HH on the “Wash stations distribution form” and give: **1 wash station container, 1 soap dish, 2 soaps and 1 Wash station flyer**.
3. The HVs also inform the household they would like to quickly discuss how to construct a stand for their wash station and how the station should be used.
   NB. If several households arrive at the same time, HVs can provide information regarding the construction of the wash station stand at the same time to a maximum of 3 households.
4. The HVs use the Wash station flyer to describe how to build and use the wash station. The following aspects should be covered:
   a. **Location and Structure:** the station should be outside, close to the home and in the shade. The wash station and soap dish are on a built, sturdy wooden structure that is secured and cannot be knocked over by children or animals.

    b. **Height:** if the household has children, very young children cannot reach the tap and soap without help, but the station should be accessible to all other family members.
    c. **Drainage:** a bowl or stones/sand are placed under the tap to prevent the ground from becoming muddy.
    d. **Water:** someone is responsible for filling the wash station with water so there is always water available for use. The wash station container should not be taken to the water, the tap is fragile. The tap should be closed whilst lathering the hands or rubbing soap on faces to avoid wastage.
    e. **Soap:** a dedicated soap is kept in a soap dish at the wash station. The soap is always clean and available for body washing when it is needed.
    f. **Night:** at night, if there is no fence around the station, it might be preferable to take the wash station container and the soap and the soap dish inside the house.
5. HV asks whether they have any question or concerns about how to set up their wash station.
6. HV encourages them to use the wash station to wash their family's faces with soap 3x a day (morning after waking, before lunch and before the evening meal) to maintain their *Faces of Dignity*.
7. HVs should **complete the "wash station distribution form"** for each HH who comes and receives the intervention materials.
8. At the end of the distribution day, <u>households who did not come to receive their wash stations</u> or <u>did not attend the Community Event</u> are visited by the HVs and inform that they can come to pick their wash station at [the chosen location] the day after. HVs should specify whether that this will be the last opportunity for them to receive their materials.
9. Similar procedures are followed on the second day of distribution.
10. Wash station distribution forms should be returned to the Berhan representative along with any remaining wash stations, so Berhan can complete the distribution of all remaining wash stations.
11. HVs should safely dispose each used facemask in a sealable plastic bag.

Appendix E. "Script for the *Faces of Dignity* Drama"

Update and Changes to the current Drama

It is expected that the Berhan Actors, in conjunction with the supervising team, will create the sections currently highlighted in 'yellow' in the drama, i.e. the **Dignity Stories**, during the intervention training (in a collaborative and iterative process). Specific instructions for these sections will be given separately ahead of the training.
Final version of these sections will need to be agreed upon by all supervision parties before rollout in the communities.

Other changes and tweaking of the current version of the drama during training are expected to happen. Yet, ALL changes made should be run through the supervision team for approval before final agreement.

Synopsis

The drama focuses on a young girl called Caltu (played by an Actor with a puppet) and the interactions between her parents (Hadha Caltu & Abba Caltu) and their neighbour (Hadha Marga) as the parents learn that to be truly attractive and dignified their family needs to wash their faces (and hands) with soap 3x a day and prioritise the cleanliness of Caltu. Hadha Caltu first tells the story of her encounter with Hadha Marga who told her that unclean faces are not attractive or dignified, and that faces and hands need to be washed with soap to maintain their dignity. In the next scene, Hadha Caltu discusses her conversation with Hadha Marga with her husband and they decide to wash Caltu's face with soap and water. They later realise that her face is dirty again and, following Hadha Marga's advice once more, they come to the conclusion that they should wash Caltu's face and hands 3x a day. At the end of the drama, Hadha Caltu and Abba Caltu are recognised as doing their bit to represent their community now they are washing their own and Caltu's face and hands three times a day with soap. Helping ensure their community is dignified makes them feel good!

Key messages

The *Faces of Dignity* campaign is about face (and hand) washing with soap. People are encouraged to <u>wash faces and hands with soap x3 a day</u> (after waking in the morning, before lunch and before the evening meal). Faces should be washed <u>thoroughly, removing all discharge.</u> This means that caregivers should support pre-school children during face washing. If a face is <u>wiped</u> to remove discharge, hands should be used instead of clothing or rags, and hands should be immediately washed with soap after the face is wiped. We are promoting washing faces together as a family to try to encourage habit formation and model the ideal behaviour, but pre-school children are the priority.

The drama aims to motivate people to adopt these behaviours by attaching new motives (dignity i.e. the root motives of *affiliation / status*, and to a lesser extent beauty) to face washing as opposed to promoting face washing with soap as a health behaviour. Face washing, and the use of soap, should come across to the audience as a really positive behaviour that makes them feel "good" inside and out.

NB. Hand washing is promoted alongside face washing, but of course there are many other times when hands should also be washed. We are not focussed on all the other handwashing messages, and just encourage hands to be washed whenever faces are (and after wiping a child's face to remove discharge). This is why the main messaging is about faces, but hands should not be forgotten.

Characters

Hadha Caltu (Caltu's mother): Female caregiver, Abba Caltu's wife – Played by the female activator
   Outfit: a long dress, woman scarf, few cultural jewelleries (including bracelet, ring, necklace?), local plastic ballerina shoes). Outfit could be made from fabric with the logo of the campaign.

Abba Caltu (Caltu's father): Household head, Hadha Caltu's husband – Played by the male activator

Outfit: jeans, a long sleeve shirt (sleeves rolled up on the elbows), formal footwear, man scarf (we could add some tools to go to the fields to materialize this?) Outfit could be made from fabric with the logo of the campaign.

Caltu: Daughter of Hadha Caltu and Abba Caltu - The "dirty" puppet, aged 5 or 6 years old

Material: Baby female puppet

Start of Drama

Scene one: Dignity

Caltu's mother (Hadha Caltu) monologue to the audience. She tells the story of her encounter with her neighbour, Marga's mother (Hadha Marga) who told her unclean faces are not attractive or dignified and that faces and hands need to be washed with soap to maintain their dignity .

Hadha Caltu:

To the audience. Actress carrying the puppet, Caltu

Look at my child, doesn't she look pretty and sweet? She is, isn't she? **Pause**

But do you know what our neighbour, Hadha Marga told me yesterday? "Caltu does not have a Face of Dignity, and therefore none of your family do either." I was shocked: "Pardon, what are you talking about? What are you saying about her face and our family's dignity?" And she told me: "I mean your child's face is dirty so she doesn't have dignity or beauty and you do not have dignity because she reflects the whole family." I disagreed, my Caltu is clean, beautiful and dignified! Please, look at her… She only has a tiny bit of dirt on her face, this little dry discharge by her eyes and nose, but that is normal for a child. What is wrong with that? What's a face got to do with beauty and dignity anyway? Isn't that weird?

Caltu: **To the audience, focussing on the children**

No mummy! It's not like that, even this little bit of dirt makes me feel bad inside. I am too small to look after my dignity. As I am too little and don't know how to help myself, please teach and help me so that I learn to take care of my dignity.

Hadha Caltu:

Hmmm… wait… wait… wait… What is my baby saying? How does a tiny bit of dirt on her face make her feel bad? I don't understand.

Hadha Marga told me: "This tiny dirt, as you called it, is not good for your child and reflects badly on your whole family. Dignity is the starting point of everything. How can your family be dignified and respected in our community if your child is not clean? How can anyone look their most attractive without first addressing their hygiene?"

Of course, I know dignity is important, who of us would disagree with that?! But I told Hadha Marga that I just couldn't see how my family's dignity could be jeopardised by a tiny bit of dirt on Caltu's face.

Hadha Marga told me to close my eyes while she helped me to understand. Come, close your eyes with me for a brief moment. I will share with you what she told me.

Encourage audience to close eyes as you speak.

Dignity is a feeling.

[INSERT HERE SOMETHING THAT TELLS A STORY OF SOMETHING THAT HAS HAPPENED THAT WOULD MAKE THE AUDIENCE – including children (if possible) – FEEL UNDIGNIFIED].

How do you feel knowing that this has happened to you? Do you feel embarrassed, unimportant and ashamed?

Now, imagine the exact opposite story.
[INSERT THE OPPOSITE STORY, WHERE THE PERSON (or child) ENDS UP WITH THEIR DIGNITY ENTACT].
Do you feel the difference?

When we act with dignity, we feel peaceful, satisfied and good inside. This is displayed in our faces and how we walk and talk.
Even if we do not have hundreds of cattle or a huge arable land, when we have our dignity we feel rich inside.
If our faces are clean, they look their most attractive and are dignified. We can be at peace. We value ourselves and know we are important and worth caring about.

Male actor rings a bell quietly off stage after she says this.

So, that's it. It's about clean faces, beauty and dignity.
Hadha Marga made me feel so upset when she said my Caltu did not have a dignified face, but now I understand why Hadha Marga talked so much about dignity. It feels so good!

Hadha Caltu changes her position and looks to be talking to herself, thinking over something that makes her worry. Then she turns towards the audience, after a small break:

To the audience
My people, I was awake the whole night. Here I am without closing my eyes for a single minute. I was worrying about how I maintain my Caltu's dignity, how I ensure our family is seen to be dignified in the community and that we contribute to making our community dignified. What if my other neighbours think we do not care about ourselves or our dignity?
Can you believe that a simple thing like a tiny bit of dirt you can hardly see affects our children's dignity and reflects badly on our family and our community?

I wanted to make it clear in my mind! I went straight to Hadha Marga this morning to ask for advice. She told me not to worry, that there is a way to give our children Faces of Dignity and ensure the whole family lives a dignified life. What do you think she said?

Wait for audience to answer while Caltu acts out face washing
That's right, she said we all need to wash our faces and hands 3x a day, especially those of our pre-school children. She also told me that we should make sure we use soap each time we wash.

Scene 2: Face and hands washing removes discharge

Abba Caltu appears on the other side of the stage, coming back to the house. Caltu's mother (Hadha Caltu) runs towards him to tell him about her discussion with Marga's mother and they take the decision to wash Caltu's face with water and soap. Happy with the result, they sit to drink coffee.

Hadha Caltu:
The mother calls her husband loudly Abba Caltu…., Abba Caltu…?
Actor arrives on stage. Mother and father greet each other
Look! Look! Are you seeing anything which strikes you on Caltu's face

Abba Caltu:

Not really paying attention at first

Sorry? What? What are you talking about, she is perfectly fine, no?

Hadha Caltu:

Really? You do not see anything…?

Would you say she is a beautiful and dignified little girl?

Abba Caltu:

Taking time to think about it and looking at his daughter There is some dirt next to her eyes and her nose… let me wipe this dirt with my shirt.

Hadha Caltu:

Oh no! Hadha Marga told me that we shouldn't use cloth or rags to clean our children's face. She said we should wash ourselves and our children's face and hands three times a day. But if we have to wipe dirt from a child's face we should use our hands and wash them with soap right after.

Abba Caltu:

Oh, so we should avoid wiping her face and always wash our hands afterwards… Fine. Oh, look one fly keeps bothering her as well!

Hadha Caltu:

**Chasing the fly from her daughter's face and shouting out. Jumps dramatically and tries to remove flies from her child's face. Should be funny*.*

I didn't notice the flies landing on my Caltu's face. Disgusting flies, ugh, I hate them! Ugh! It is disgusting to think that the flies that land on faeces are then coming to my child's eyes… I hate that! Bouuuh! I see now why Hadha Marga says Caltu can't be dignified with a dirty face… How can someone be dignified if they have faeces on their face?

She pauses

As if the idea of faeces on her face was not enough, Hadha Marga said that flies can also carry diseases like trachoma, an eye disease that can lead to a lot of eye pain and blindness. The good news is she said that the actions we take as parents and as a family can protect us all from this.

Caltu: **To the audience, focussing on interacting with the children**

I hate it so much when the flies are on me, don't you? And I can't do anything to get rid of them. Oh no! I don't want trachoma either that sounds horrible.

Hadha Caltu:

My husband, this is serious. I met Hadha Marga yesterday and she told me that our Caltu does not have a Face of Dignity and as a result our whole family is not dignified and is letting down the community.

Abba Caltu:

**Looking really astonished*.* Oh! So what I have heard around the garee is about this then?

Hadha Caltu:

Alarmed What? What are you talking about? What have you heard around the garee?

Abba Caltu:

The Faces of Dignity! Every child is asking his friends about this! "Do you have a Face of Dignity?" Everyone is talking about that. Abba Marga told me about it as well… I was not sure what all this was about. He told me something about washing faces and removing dirt and beauty, dignity and respect in the community… Doesn't he have something better to think and talk about as a leader? He was expected to raise issues like our agriculture, building strong community through our religion and so on.

Hadha Caltu:

Cutting her husband talking Yes, yes, yes, it is all about this. How do you expect to have a strong community without starting first with dignity?

Hadha Marga told me we should wash our faces and hands with water and soap 3 times a day. And now, you are telling me that everyone in the community is already doing it and knows about it. Oh, Abba Caltu, if we want our family to have Faces of Dignity we need to do the same! I usually use just water.

Caltu: **To the audience, focussing on interacting with the children**

I want a Face of Dignity, don't you all want a Face of Dignity?

Abba Caltu:

Pausing to consider his wife's plea. Looking to her and Caltu and agreeing
Yes, you are right. Dignity is most important. Abba Marga also explained to me that washing faces and hands with soap 3 times a day was important to prevent sickness. Washing with soap can prevent trachoma, diarrhoea and other illnesses too.

Hadha Caltu

To Abba Caltu
It is early morning still and Hadha Marga said the first time we need to wash is when we wake up. Let us wash and let me wash my Caltu, please hold her for me while I get the water.
Actress gives the puppet to the actor and goes to take water. Leaves stage

Abba Caltu:

Alone on stage with Caltu Caltu's nose is really stuffy… Oh! And all those flies… Having a Face of Dignity, is it all about this…?

Hadha Caltu:

*Back with jerry can, jug and collector. Starts to wash Caltu with water only. Abba Caltu uses the prop to highlight the face washing [TBD].
To the audience Washing Caltu's face is an easy way to make her dignified and it makes her feel fresh and clean as well. I have done the right thing and that makes me feel good too!
Actress sings ***[INSERT THE DIGNITY SONG]***

Caltu: *To the audience, focussing on interacting with the children*
Great! Mummy forgot to use the soap. Phew! I don't like soap when it gets in my eyes.
But wait… I don't feel clean and dignified like mummy said… I still feel dirty as if she didn't wash my face at all. Why did she bother to get the water and make me wet if I feel just the same? Maybe it isn't so great that mummy forgot the soap after all?

Hadha Caltu:

Ask to the audience
I don't feel as good as I thought. I have the impression I am forgetting something to help my Caltu be a dignified little girl… What am I forgetting? *Audience answers* Soap! Soap! Of course.

Abba Caltu:

I don't understand the whole story here Hadha Caltu, we always wash our faces every morning when we wake up from our sleep. So why are we expected to wash our face and hands with soap let alone the three times?

Hadha Caltu:

Yes, you are right my husband. What I am talking about is adding soap whenever we wash our face and hands so that our face keeps clean longer.
Abba Caltu, now come here and watch Caltu while I fetch the soap. I told you Abba Caltu, soap is essential! We need to remind each other until we get used to washing our face and hands with soap three times a day.
Gives Caltu to the actor and run comically to look for the soap

Abba Caltu:

Right, right, washing with water and soap… But the soap … what is the purpose exactly? You know I am not really keen on wasting soap, if…

Hadha Caltu:

Cutting her husband talking Wasting soap, wasting soap you say? What are you worried about huh? Is it about the money you spend to buy soap? How can you put a price on our children's dignity? Hadha Marga told me that it is the only way to get rid of all discharge, even the tiny ones, and to be truly attractive and dignified. If we do not use soap whenever we can, it is like not washing at all. And anyway, do you want our children not to be dignified when all the other children are washed with water and soap? And what about trachoma? If we forget to wash Caltu's face with soap the flies will come.

Caltu: *To the audience, focussing on the children*
I really want to be clean and dignified and not get sick. I wonder if it is worth not resisting the soap?

Abba Caltu:

Hadha Caltu! Are you mad? Are you really going to use soap and burn Caltu's eyes?

Hadha Caltu:

Enough, Abba Caltu, first you do not want me to use soap at all and now this! Do you want her and our family to be dignified? To get rid of the dirt, discharge, trachoma and the flies? Do you want us to be respected in the community…? Do you want us to stay healthy?

Abba Caltu:

Sheepish, hesitating Yes… Of course, yes… I want to be dignified and respected, and our family and… Caltu as well.

Hadha Caltu:

Good, so let me use soap. And do not worry, I am not stupid! This is a dedicated soap for body washing, it is not harsh. It will not hurt Caltu or make her skin dry. I will only use the right amount, and will not waste it.

Actress takes the puppet again and start to wash, describing in detail what she is doing. Actor makes some comments

Hadha Marga said I need to rub and wash well around the eyes and nose, let me try this. With the body soap and Caltu closing her eyes tightly, soap will not go into her eyes. And if Caltu is crying despite everything, I have another method to calm her down.

Actress sings a song to placate Caltu. Actor and actress do the actions for the song

[INSERT THE DIGNITY SONG]

Caltu: **To the audience, focussing on the children**

Actually, that wasn't too bad! I closed my eyes tightly the whole time mummy was washing my face and it was over quickly!

Now I feel fresh, clean and dignified, and I smell good too! I didn't feel like that without the soap. Don't you want to feel this good too?

Abba Caltu:

Great, you are done, let's drink coffee.

Hadha Caltu:

Not so fast Abba Caltu! We haven't finished yet. Look…

Actress turns to audience and says there is one more important thing that Hadha Marga taught her

Can you please turn to your family members and shake their hands for a minute? Don't let go of their hand until I tell you.

Actress insists until people in the audience shake their family members' hands. The actress continues to speak as people shake:

Abba Caltu, come, shake hands with me.

Abba Caltu should mime disgust as she talks very dramatically Imagine all the things this person you are shaking hands with could have been doing before shaking hands with you and imagine if you keep shaking hands with other people outside your family members. They didn't wash their hand before they shake yours so everything they have touched so far today is still on their hand. They might have cleaned up their child's faeces, cleaned snot from their child's face, cleaned the compound and thrown rotten food away.

Actress tells them to stop shaking How do you feel now? It's disgusting right?! Hands can look clean but they are not always! The dirt and discharge from their child's eyes are one of the many disgusting things that might have transferred to your hands when you were shaking hands. It is not just flies that can carry trachoma from eye to eye. Hands can do this too. In fact, a lot of diseases can be spread by dirty hands, like COVID-19 and diarrhoea. Giving each other diseases is not a very dignified way to behave and is not how we look after one another in the community. We will give you soap before you go home today so you can wash faces and hands directly when you get home.

So you see, I should not forget to wash Caltu's hands whenever I wash her face!

Actress mimes washing the puppets hands with soap and the couple talk about how it is easy for them to wash their hands when they wash their faces because the soap is on their hands when they wash their face, but they need to wash Caltu's hands for her as they do not otherwise get soap on them.

Caltu: **To the audience, focussing on the children**

I am so relieved that mummy has washed my hands properly, I am touching so many hands everyday! I don't want to faeces, diseases or anything from the other people!

Abba Caltu:

Waouh! Look Caltu is quiet now and a beautiful and dignified little girl. We know she is dignified and that is what is most important. Now I see what the "Faces of Dignity" Campaign is all about.

Hadha Caltu:

Yes, it is what Hadha Marga told me, from the "Faces of Dignity" Campaign. It must be important because ***[INSERT NAMES OF LOCAL COMMUNITY, RELIGIOUS AND CLAN LEADERS]*** as well as the Health Volunteers and Health Extension Workers are supporting this.

[If any of these people are in the audience, ask them to stand briefly and agree]

Abba Caltu:

That is really impressive. Previously, the reason why I was complaining about using soap was trying to reduce our financial expenses… But I get it now, it is all about having Faces of Dignity, being a dignified family and being a dignified community. Between our family's dignity, our Caltu's dignity and the money for soap, the 10ETB for the soap is no hard choice. Dignity should always come first. It is the most important thing we can do for our children. I see now that our children's dignity is also important. They are representing our family in the community all the time, as soon as they can run out to our neighbours' homes. I would hate to think people are thinking badly about our family or that we do not represent our community well. Hadha Caltu, we will buy soap and wash as a family. We will wash Caltu's face and hands with water and soap as she is too young to take care of her own dignity.

Hadha Caltu:

You are right, that is a good decision. I am going to prepare coffee to celebrate our dignified family.

**Actress goes to look for the coffee material while the actor is staying with Caltu, humming a song and playing with her, joking about her Face of Dignity*.*

Abba Caltu:

To the puppet Who has a nice *Face of Dignity*? My Caltu, yes, my Caltu!

Caltu: **To the audience, focussing on the children**

At first, I hated the idea of soap and cold water on my face, but now I know that it makes me feel so good, so refreshed, clean and dignified. I am so lucky my mummy and daddy love me so much to take such good care of me and to make sure we always have soap for washing.

Hadha Caltu comes back and prepare coffee. They drink coffee together, Caltu playing on her father's arms

Scene 3: Washing three times a day with soap

Hadha Caltu and Abba Caltu are satisfied with how they have washed Caltu's face. But, they suddenly realise that Caltu's face is dirty again. Thanks to Hadha Marga's advice, they come to the conclusion that they should wash Caltu's face 3x a day!

Caltu: **To the audience, focussing on the children**

I have been running around and I don't feel clean and dignified anymore. My parents don't seem to be aware or doing anything about it. How do I get their attention? I know, I must cry…

Abba Caltu:

While drinking coffee, Abba Caltu realises that his daughter is starting to cry and feel discomfort again

Hadha Caltu, look, it has only been a few hours but our daughter does not seem good again… She is crying.

Hadha Caltu:

Oh! She has a stuffy nose. Look at her eyes, there is discharge as well. I didn't think she could get dirty again this quickly!

Abba Caltu:

Oh! Again! Already?

Hadha Caltu:

Shall I keep washing her all day? Isn't once enough? Let me go to Hadha Marga, maybe she has heard a solution for that from the Faces of Dignity Campaign or the Health Volunteer.
**Actress is leaving the stage to get some advice and leaves Caltu with the actor*.*

Abba Caltu:
**Alone. Actor is complaining about his daughter crying. He tries to calm her down, humming and saying nice words. Shortly, Hadha Caltu comes back*.*

Caltu: **To the audience, focussing on the children**
I don't understand. Mummy washed my face this morning, it is only lunch time and I feel dirty and have sticky eyes again.

Hadha Caltu:
I have told the whole story to Hadha Marga. I said that we washed Caltu's face and hands with soap this morning but it seems like her face needs washing again. She reminded me, that washing only once a day in the morning is not enough because dirt and discharge comes back again and the face loses her beauty and dignity. She said that to keep the dirt away and prevent trachoma we should wash three times each day - in the morning when we wake up, but also before lunch and before dinner when we wash our hands, and always with soap. Hands need to be washed whenever faces are washed as we touch our faces so much! She said that this is important so we all have Faces of Dignity and live dignified lives.

Abba Caltu:
In disbelief How many times a day, have you just said?

Hadha Caltu:
Three times a day, throughout the day, and with soap! Let me go and get the water and soap. We will eat lunch soon and we will wash our hands, it will be easy to wash our faces then too.
Actress leaves the stage to get the wash material, including soap
**Starts*

Abba Caltu:
**Actor alone, holding Caltu, asking his child where her Face of Dignity has gone*.*
Oh my Caltu, it seems unnecessary to wash this much, what can be the benefit?

Caltu: **To the audience, focussing on the children**
Oh Daddy, don't say that! I feel so fresh and good inside and out after my face is washed with soap but that feeling doesn't last the whole day!

Hadha Caltu:
**Actress comes back and starts washing Caltu's face and hands again*.*
[INSERT THE DIGNITY SONG]
Here we go again, what are you thinking Abba Caltu? Clean is always beautiful and dignified. To get truly clean we need to rub very well around the eyes and nose.
So we need to remember, if sometimes I am not at home, in the morning, before lunch or before dinner, please remember to wash Caltu's face and hands, on time, with water and soap. Even though her sister is in school we should not forget to remind her as well. In fact, we need to do it together as a family so we can help each other and set a good example for the children.

Abba Caltu:
Aha! Now you put it like that I understand fully. Caltu's beauty comes from her dignity, and dignity starts with a clean face. I will also make sure there is always soap for her. She is the face of our family. If we want to be dignified she has to be dignified first. She is going to be the priority now.

Hadha Caltu:
Good. I am sure she will get used to face washing if we do it three times a day. Soon she will not cry anymore. Even if she cries, I will continue because I am her mother and I know what is best for her. I will remind her to keep her eyes tightly closed so the soap does not sting her eyes. We must all wash, the whole family! We must also wash hands whenever we wash faces. These behaviours will set a good example for her and will make sure our family is dignified.

Abba Caltu:
You are right and I will buy her a dedicated soap for face washing as well so it is always at home when it is needed. Great! Let me go to the fields now.
Actor leaves the stages. Actress alone with Caltu

Scene 4: Becoming Faces of Dignity role models in the community

Hadha Caltu and Abba Caltu are recognised as role models in the community now they are washing Caltu's face (and hands) three times a day with soap. It makes them feel good!

Hadha Caltu:

Alone on stage. Hugging Caltu and repeating for herself

I wash my Caltu's face. I use soap. I rub well around her eyes and nose so I remove dirt, discharge, flies and disease. Three times a day, in the morning before breakfast, before lunch and before dinner. I will do that for my Caltu so everyone wants to play with her and she has a *Face of Dignity* in the community and is protected from trachoma….

Hadha Caltu is interrupted by her husband coming back from the field… he is carrying a wash station and soap in a soap dish which he puts down on a table as he enters

Abba Caltu:

Interrupting Hadha Caltu's monologue, putting his arm to the sky and screaming loudly and happily

Bravo! Bravo! *(To be translated: moral)*

Hadha Caltu:

What is that?

Abba Caltu:

Bravo! Bravo! Oh, Hadha Caltu! I was outside, working in the fields, when Abba Marga and Abba Kedir came to congratulate me. I was so surprised, so I asked the reason for such nice words. Then, they told me: "Your wife has become a role model for this community since she is washing Caltu's face three times a day with soap. Your family's Faces of Dignity are known to everyone." My wife, bravo! Bravo!

Hadha Caltu:

Actress now smiling and looking happy Oh my dear husband, there is always something which keeps telling me that you love me. That is true, I know that you love me my husband. But, don't you think you are bragging everywhere?

Abba Caltu:

Yes of course my wife, I am bragging! I was so happy when my friends told me that we are considered as role models in our community now. I brag because they told me that now we have Faces of Dignity, we are respected by the whole community. I will continue to do the same because they told me that our name is being given as an example of a dignified family. People are talking about us, saying good things about how you are maintaining our children's dignity and also protecting them from getting trachoma. It is great that we prioritise little Caltu and wash her face first as she is running everywhere representing our family. And they said to me: Bravo! Bravo! That's why I am also saying Bravo! Bravo!

Hadha Caltu:

To the audience I have changed and feel peaceful and better inside because I know that my family leads a dignified life. Dignity is so important don't you think? It is priceless. I expect that you will be telling your neighbours who could not attend this event all about how they can make sure their family has Faces of Dignity, maybe at your next coffee ceremony or next garee meeting? You see, Hadha Marga taught me about this, so I am telling you. In my opinion you should now do the same and tell others. Let's show everyone that we live a dignified life here. It's so simple: just wash your children's faces with soap three times each day! Wash their hands when you wash their faces!

Abba Caltu:

Talking to his wife Don't forget the 3 times! They should wash in the morning before breakfast, before lunch and before dinner. The whole family, but always children first! They will all have Faces of Dignity and feel good inside as well as look good outside.

Goes over to the wash station Look here what I have brought for our family. A wash station! We can make sure we continue as we have started by using this wash station and keeping soap clean in this dish so it is always available for face and hand washing. Using this wash station will make face washing so much easier. There will always be water available and we can use the tap to control how much water we are using. We will make sure we always fills it with water when we come back from

collecting water and keep the soap in the soap dish next to it. Just seeing this wash station will help us always remember!
To the audience Raise your hand if you would like one of these to help you remember to wash faces and hands? Then your family can also show that they live a dignified life!

Oh, it's already getting dark so let's wash our face and hands before we get to our dinner. **Picking Caltu in his hands** Come on my precious child, you have no idea how you made me feel proud and dignified. Who would make me think about all these excellent ideas of protecting personal hygiene if you were not here?
Talking to his wife
And my wonderful wife it is your effort that made our family dignified, clean and healthy. Come join me and our Caltu to wash our face and hands.
The father helps Caltu to wash face and hands using the station, then wash his own face and hands. The mother washes her face and hands as well.

Caltu: **To the audience, focussing on the children**
I am used to washing my face and hands with soap 3 times a day, morning, before lunch and before my evening meal at the nice, new wash station. It is normal for my family and we all wash together whenever we can. The rest of our community is doing it too! When my face is washed with soap, I feel good inside and so proud to be a member of such a dignified family! Now this is our habit we will always do it!

End of the Drama

EVENT 2 – FAMILY FORUM 1

ACTIVATOR MANUAL

**Purpose**	This event is designed to build washing related knowledge, skills, and motivation, and empower households to construct wash stations to aid habit formation.
**Responsible parties**	1 trained Activator + 1 trained Health Volunteer (2 HVs will assist the Activator in each cluster to spread the work load)
**Participants**	All household members living within the 'yolk' of an intervention cluster who attended the Community Event – Split into groups of 5 households.
**Location**	A HH compound (selected in advance when HHs are informed the time and date for the forum)
**Duration**	1h30
**Timing**	Workdays (except Friday morning), 9am to 1pm and after 3pm

Preparation

Day before the event

- Mobilise HVs to recruit 5 neighbouring households living in the 'yolk' of the intervention cluster according to the census list. If 1-to-5 Groups ('ijaarsa olla') exist in the cluster, they should serve as a basis to form groups of 5 neighbouring households. Otherwise, grouping of households will be done according to geographic location. As much as possible, households will be grouped considering any local sensitivities. Groups can range from 4 to 6 HHs to adapt to local circumstances.
- HV to ask one household to host the event. If 1-to-5 Groups exist in the community, select the house of the 1-to-5 Group Leader (if the compound has space to hold the event).
- Communicate time and location of the event to each participant household.
- Inform each household that the whole family should participate and that they will receive some gifts if they stay for the whole event.

Setting

The sketch below illustrates the ideal setting for the forum. Activators are free to amend the set-up according to each HH setting. Any setting should respect the following criteria as much as possible:

- Participants should be able to see each activity and the flipchart easily.
- Participants should be sat in the shade of a tree or of the house, under a canopy whenever possible.
- Materials for further activities should be well-organised and kept out of the way but easily accessible.
- Participants should sit with their household members and observe a physical distance of at least 2m with other households.

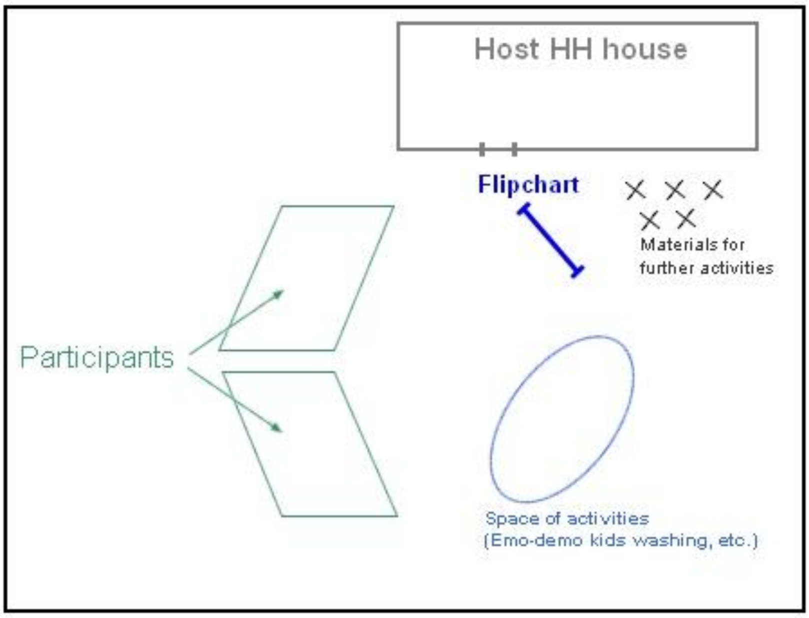

Materials

**General**	– COVID-19 preventive materials: cleaning solution in a spray (x1), alcohol-based sanitiser, facemasks (for activator and HV), sealable plastic bag to dispose masks<br>– Flipchart<br>– Caltu's puppet in its cover<br>– Pen<br>– Notebook<br>– Cell phone or device to play the Dignity Song and the recorded Testimonials<br>– Ask the hosting household to borrow a stool or a chair for installing the wash station for the emo-demo
**Trachoma Transmission Routes**	– Transmission board (magnetic board and laminated sheet)<br>– Paper clips (x4)<br>– 9 magnets: fly magnets (x2), discharge magnets (x4), hand magnets (x2), scarf magnet (x1)
**Face Wipe Emo-Demo**	– Moisturizing cream + Foundation powder with a brush<br>– Green clay for face<br>– Face wipes (x16)<br>– Branded wash station borrowed from the HV (if possible, filled with water) + if possible, branded wash station borrowed from the host HH<br>– 20L jerrycan full of water<br>– Water collector<br>– 14 paper clips<br>– 2 laminated sheets of white paper to present the wipes<br>– 2 'Washing protocol sheets: Water only and Water and Soap' to present the wipes
**Wash stations**	– 15 drawings of the puppet to be coloured in<br>– Wax crayons (x15) *(NB. Crayons will be taken back from the children at the end of Family Forum 1. Children will keep their wax crayon at the end of Family Forum 2)*
**Testimonials**	– Activator's phone with the recorded testimonials on it
**Dignified Day Pledge**	– 5 Dignified Day posters<br>– 10 small nails

Set up

Day of the family forum

- Do you have all materials required for all activities?
- Is equipment working?

  Tablets have full batteries with testimonials for that cluster on the tablets.
- Has the Activator put cream and foundation powder on his/her face (and washed their hands afterwards)?
- Has the flipchart been placed in a suitable location?

Activities

Activity 1: Introduction

Purpose

- To provide introduction to the forum.
- To address any concerns or questions arising from the Community Event.

What to do

1. Install the flipchart – FAMILY FORUM 1 COVER image. Install Caltu's puppet next to the flipchart on its cover.
2. Play the Dignity Song on a cell phone or any other device while participants are arriving.
3. Advice community members to sit with their household members and respect a physical distance of at least 2m with other households.
4. Greet participants and welcome them to the forum.
5. Complete attendance sheet.
6. Introduce yourselves and remind the group that they saw you or your colleagues at the Community Event.
7. Ask participants to raise their hands if they went to the Community Event.
8. Choose one participant who came to the Community Event to summarise its content and key messages for participants who were not present.
9. Ask if anyone has any questions about anything they heard at the Community Event. Answer their questions before proceeding.
10. Say that they have already started to learn how to have *Faces of Dignity*, and that we will continue this learning today.
11. Tell participants that the event should not take more than 1h30. Say that they will be given some small gifts for the entire family, so it is very important they stay until the end if they can.

Activity 2: Trachoma Transmission Routes

Purpose

- To understand how trachoma is transmitted, i.e. flies, fingers and fomites.
- To understand regular and thorough hand and face washing with soap reduces the risk of disease.
- To perceive discharge as disgusting and dangerous to health.

What to do

1. Turn the flipchart – TRACHOMA TRANSMISSION ROUTES page.
2. Ask participants what they learnt about how trachoma can get from one eye to another eye at the Community Event. Correct participants if you need to and congratulate them on their answers (they should mention flies and hands).
3. Set up the Transmission Board (i.e. fixed the laminated sheet on the magnetic board using the paper clips) and explain that we are going to talk a little more about transmission so that everyone understands why it is so important to get rid of discharge.

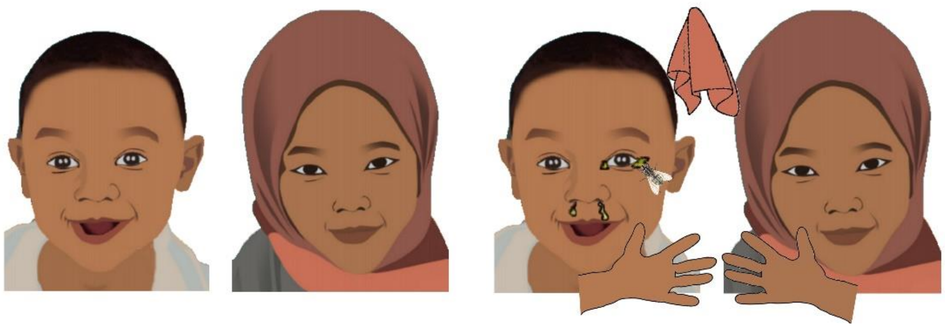

Trachoma is carried in discharge

1. Tell participants that trachoma is a small germ and is carried in discharge that we can often see on people's faces.
2. Ask some children to come and show on the Transmission Board where they usually see discharge on someone's face. They should show discharge below the eyes and nose. Help them if you need to. Give them 4 discharge magnets to put under the boy's eyes and nose.
3. Ask participants whether they agree that discharge carrying trachoma can be found under the eyes and the nose of children.

> **Demonstrate using the board**
> 1. Put/Move the magnetic discharge under the eyes and the nose of the boy's face and explain what you are doing.

First transmission route: Flies

1. Introduce the first route of transmission, flies.
2. Ask some children to come and put the 2 fly magnets on the boy's face on the Transmission Board. They should show flies under the eyes and nose. Help them if they need it. *Invite the children (or their caregivers if the children are too young) to assist with the rest of the demonstrations by moving the magnets when you ask them to. Make sure the rest of the participants can see the board.*
3. Ask participants whether they agree that flies land under children's eyes and noses.

> **Demonstrate using the board**
> 1. **"A fly lands on this discharge under the boy's eyes because it likes to eat it."** – Make magnetic fly travel to the boy's eye.
> 2. **"Discharge from the eye gets on the fly."** – Pick up the discharge magnet using the fly magnet.
> 3. **"The fly then lands on another child's eyes."** – Make the magnetic fly travel towards the girl's face and put the fly next to the clean eye.
> 4. **"Guess what happens?"** – Let people answer. **"Yes, when the fly lands on the girl's face the discharge rubs off the fly onto the girl's face. Her eye will become dirty as well." –** Take the discharge off the fly and put it under the girl's eye.

4. Ask participants whether they agree that flies can transmit discharge carrying trachoma.

Second transmission route: Hands

1. Move the discharge from the girl back to the boy.

2. Inform participants that we are now going to look at a different way discharge can spread from the boy's eye to the girl's eye: hands.

> **Demonstrate using the board and with the help of any children in the group.**
> 1. **"Now imagine those two children are playing together. One has discharge and the other one is clean. While playing, the boy touches his dirty eyes or nose."** – Lift the magnetic hand and place it on the boy's face so it picks up the discharge magnet.
> 2. **"His hands become dirty and now carry the discharge with trachoma."** – Show the dirty hand (with the discharge magnet) to the crowd.
> 3. **"While playing, the boy will certainly touch the hand, or even the face of the girl."** – The two activators put the hands in contact and make sure that the discharge magnet moves onto the second hand.
> 4. **"Then, what's going to happen?"** **Let people answer** **"The discharge will move from the boy to the girl, who is likely to touch her face at some point."** – Show the discharge on the hand of the girl to the crowd (show that the magnet has changed hand).
> 5. **"Eventually, she will put the discharge with trachoma on her own face and get dirty as well."** – Make the girl touch her face and move the magnetic discharge to under the eyes.

3. Ask participants whether they agree that hands touching dirty faces can also transmit trachoma.
4. Mention that caregivers can also easily transmit trachoma between their children if they touch them one after another, notably when they are wiping their children's faces to remove discharge.
    a. Advise caregivers to always wash their hands with soap directly after wiping a child's face to remove discharge from their hands.

Third transmission route: Clothing

1. Move the discharge from the girl back to the boy.
2. Inform participants that we are now going to look at one more way that discharge can spread from the boy's eye to the girl's eye: our clothes.

> **Demonstrate using the board**
> 1. **"Now imagine that these two children are siblings. One has discharge and the other one is clean. Their mother comes and sees discharge on her boy's face. She uses her scarf to remove his nasal discharge."** – Use the piece of cloth/a scarf magnet to "wipe" the discharge under the boy's nose. Attach the magnetic nasal discharge to the scarf.
> 2. **"The scarf of the mother becomes dirty and now carry the discharge with trachoma."** – Show the dirty scarf (with the nasal discharge magnet) to the crowd.
> 3. **"Later on, the mother will certainly use her scarf to touch the hand or the face of her daughter."** – The activator puts the scarf next to the eye of the girl.
> 4. **"Then, what's going to happen?"** – Let people answer. **"The discharge will move from the scarf to the girl's face."** – Put the nasal discharge magnet from the scarf under the girl's eye.

3. Ask participants whether they agree that cleaning discharge using clothing can also transmit trachoma.
    a. Advise caregivers to always use their hands to wipe discharge on their children's face and avoid using a cloth or a scarf. Remind caregivers that they should always wash their hands with soap directly after wiping their children's faces to remove discharge from their hands.
4. Ask participants to summarise the 3 ways they have learnt about how trachoma can be transmitted. – *Let people answer. Point to the props (fly, hand and scarf) as each transmission route is mentioned.*
5. Congratulate the participants on their understanding.

Face washing to limit transmission

1. Ask participants what they can do to get rid of the trachoma-carrying discharge and maintain their children's dignity. They should answer face (and hand) washing. If not, probe for what was done in the community event.
2. Ask participants to raise their hands if they agree that they can wash their children's faces to get rid of disgusting discharge and maintain dignity.
3. Tell participants that faces and hands need to be thoroughly washed to get rid of discharge, especially around the eyes and nose. Pre-school children need to be helped so they can wash well. Caregivers should wash their hands with water and soap when supporting their children and each time they are wiping their children's faces.
4. "Wash the children's faces" on the Transmission Board using water and soap by removing the magnetic discharge.
5. Spray the Transmission board and all magnets with cleaning solution.

Introduce the Face Wipe Emo-Demo

1. Ask participants if they think that they are always able to see all the dirt and discharge on a person's face.
2. Tell the group that this will be the topic of the next activity, and that some discharge is not easy to see but it can still carry trachoma.

ACTIVITY 3: FACE WIPE EMO-DEMO

Purpose

- To perceive discharge as disgusting and dangerous to health.
- To understand germs on hands and faces (particularly of children) are invisible and cause trachoma.
- To understand that to achieve truly clean hands and faces, soap must be used.
- To understand regular and thorough hand and face washing with soap reduces the risk of disease.
- To perceive soap as important to use each time faces are washed.
- To perceive effectively washing face at least 3x per day as important, all year around.

What to do

1. Turn the flipchart – FACE WIPE EMO-DEMO page.

Note for the activators

The Activator should put foundation on their face before the forum.
The HV should put some dirt on his/her face at the start of this activity.
Both should have washed their hands with water and soap before starting.

Demonstration 1: A clean face is not a clean face!

Activators’ demonstration

1. Ask participants/children if they see any difference between the face of the Activator and the face of the HV.
2. Ask children to point towards the person with the dirty face.
3. Explain to participants that you are going to use wipes to confirm what they have just said. Explain that the wipe is just a cloth that is a bit wet.
4. <u>Wash your hands with soap before starting</u>.
5. Ask participants which wipe they expect to be dirty.
6. The activator and HV should <u>wipe the</u> <u>RIGHT side of their faces</u> and pin the wipes onto a laminated piece of white A4 paper
7. Ask the audience what they see: both wipes are dirty. Is this a surprise?
8. Say that faces can look clean even when they are not.
9. Ask the audience if they agree with this.

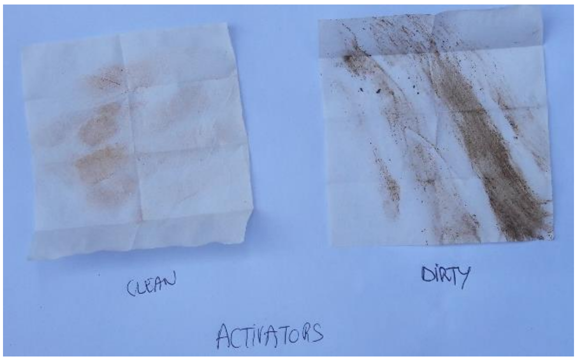

Children’s demonstration

1. Explain to participants that this is true in their community as well, even for children who seem to have clean faces.
2. Put facemasks on and explain to participants why you are doing this because you will get near to their children.
3. <u>Select up to 4 to 6 children</u> aged approximately <u>6 to 9 years</u> old.
4. Explain that you will repeat the activity to see if there is any invisible dirt or discharge on them as well.
5. <u>Turn away from the crowd</u> (so they cannot see which child is the dirtiest) and use a clean wipe to <u>wipe the RIGHT side of each child’s face</u>.
6. Remember which wipe comes from which child, but do not share this with the group.
7. Clip each used wipe onto the laminated piece of white paper and show the dirty wipes to the group.
8. Ask them again if they agree that a face can look clean but it is not necessarily clean and therefore is not a face of dignity and may also carry trachoma.

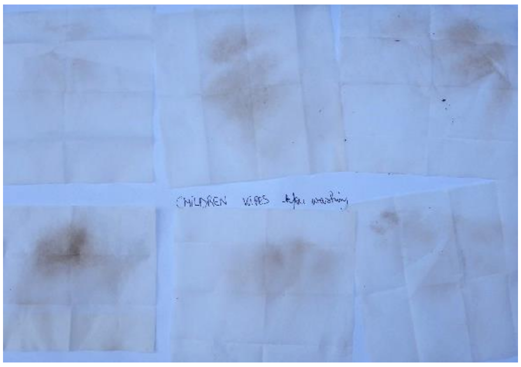

NB. It is unlikely but possible that all children's wipes will be really clean/white. If this happens congratulate the children and parents on their Faces of Dignity and do Demonstration 2 on yourselves and not the children (the Activator with foundation should wash with water only, while the HV with obvious dirt washes with water and soap).

Demonstration 2: A face is clean and dignified only if we use soap!

1. Ask participants what they could do to make sure their children's faces are never dirty and undignified when they appear clean.
2. Tell participants you will do another experiment to show them how children's faces should be washed. Say that half the children will be washed with water and the other half with water and soap, because these are the different ways that we wash our faces.
3. Set the wash station on a stool borrowed to the hosting household and place a water collector on the ground under the tap. Place the soap in the soap dish on top of the station. (If possible, ask the hosting household to borrow their wash station to speed up the face washing process).
4. Ask the children with the dirtiest face wipes from the previous exercise to wash their faces with water, while the other children wash with water and soap. Encourage them to turn the tap of whilst they lather the soap.
5. Wait for the children's faces to dry (wait a minute or two).
6. Turn away from the crowd with the children who washed with water and wipe the LEFT side of each child's face thoroughly (including near the ear / side of neck) with a clean wipe.
7. Clip the wipes onto the laminated "Water only washing protocol" paper.
8. Repeat with the children who washed with water and soap. This time take care not to wipe the face as thoroughly, the wipes should appear clean!
9. Present the wipes to the group on the laminated "Water and soap washing protocol" paper.
10. Ask participants to explain what they see. They should conclude that faces are not clean unless they are washed with soap.
11. Remind participants that soap is required for true cleanliness and dignity.
12. Ask participants to raise their hands if they
    a. Agree that soap is the only way to achieve *Faces of Dignity*.
    b. Value their dignity and the dignity of their children.
13. Ask the group if they want to wash faces with soap regularly so their whole family have *Faces of Dignity*.
14. Conclude this activity by reminding participants that:
    a. A face might look clean but can be dirty anyway (point at appropriate wipes).
    b. Dirt and discharge, as demonstrated in the transmission activity, visible or not, can carry trachoma.
    c. Only a face washed with water and soap is a true "face of dignity", i.e. a face which is really clean (point at appropriate wipes).
15. Safely dispose your facemasks in a sealable plastic bag.

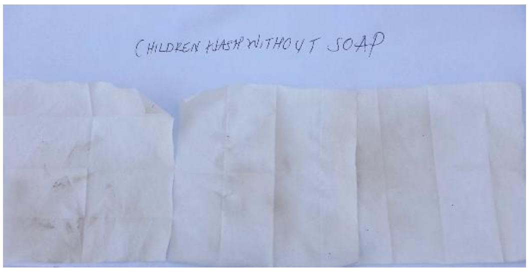

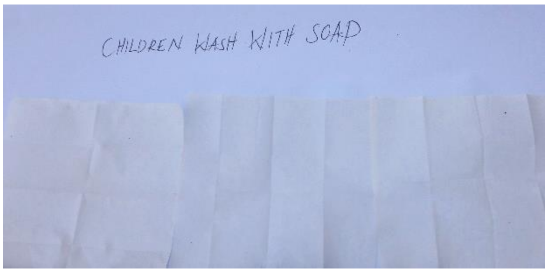

Activity 4: Wash Stations

Purpose

- Can construct a functional wash station.
- Functioning wash station is consistently available and accessible.
- Soap is consistently available and accessible for washing.
- Water storage, soap storage and wash equipment are consistently available and accessible.

What to do

1. Turn the flipchart – WASH STATIONS page.
2. Before starting the Wash Station activity, propose to children (aged 2 to 7 or 8) to colour in a drawing of the puppet explaining about face washing. Tell the children they will be able to keep their drawing to remind them about when they should wash their faces to become *Faces of Dignity*. Put the puppet in a visible place. Give one drawing to each child and a few wax crayons disinfected with the cleaning solution.
3. Introduce the wash station activity:

> **To keep our *Faces of Dignity*, we have just seen that we need to wash our faces, especially our eyes and noses, with water and soap. In the community event, we saw that it is not always easy to gather all the materials to wash – sometimes it can be hard to find soap when we need it. It is hard to keep the soap clean while we wash. It is also easy to forget to wash or to use soap. Here (point to it) you can see a wash station like the one that you got just after the Community Event.**

4. Bring forward the wash station and the soap in a soap dish used in the Face Wipe Emo-Demo (keep it on the stool if possible, so everyone can see the station properly).
5. Ask participants what they like about using a wash station (e.g. when they use them in markets and restaurants) and discuss whether a wash station makes it easier to wash faces and hands with soap. Mention that keeping the soap in the soap dish at the wash station means that a dedicated soap is clean and available for body washing when it is needed.
6. Say that we will now discuss how to construct a stand for the wash station they received at the Community Event and how the station should be used.

Location and Structure

1. Turn the flipchart – WASH STATION 'LOCATION' images.
2. Ask the group to describe the images.
3. Turn the flipchart -– WASH STATION 'STRUCTURE' images.
4. Ask the group to describe the images
5. **Summarise: a 'good' location to keep the station is outside, close to the home but in the shade. The wash station and soap dish are on a built, sturdy wooden structure that is secured and cannot be knocked over by children or animals.**
6. Ask participants if they have any suggestions or concerns about the location or structure. Reassure them that building a wooden structure does not take that long. In neighbouring communities, people have on average spent two hours collecting wood and building the wash station stand.
7. **Guide the discussion** to cover the following potential concerns:
    a. Outside location: because most face washing takes place outside.
    b. Shady location: to protect the wash station container from damage. Could build a roof if area near the house is exposed.

Height

1. Turn the flipchart – WASH STATION 'HEIGHT' images.
2. Ask the group to describe the images.
3. **Summarise: very young children cannot reach the tap and soap without help, but the station should be accessible to all other family members**.
4. Ask participants if they have any suggestions or concerns about the height of the structure for the wash station.
5. **Guide the discussion** to cover the following potential concerns:
    a. Accessibility: which age children should be able to access the station on their own? Who should be prevented from accessing the tap or soap? Who will help them wash?
        i. Is there anyone else in the family who needs to be thought of? e.g. disabled.

Drainage

1. Turn the flipchart – WASH STATION 'DRAINAGE' images.
2. Ask the group to describe the images.
3. **Summarise: a bowl or stones/sand are placed under the tap to prevent the ground from becoming muddy.**
4. Ask participants if they have any suggestions or concerns about drainage for the wash station.
5. **Guide the discussion** to cover the following potential concerns:
    - Drainage vs allocating a dedicated water collector.
    - Avoiding 'splash back' from a collector.

Water

1. Turn the flipchart – WASH STATION 'WATER' images.
2. Ask the group to describe the images.
3. **Summarise: someone is responsible for filling the wash station container with water so there is always water available for use.**
4. Ask participants if they have any suggestions or concerns about keeping water ready for use at the wash station.
5. **Guide the discussion** to cover the following potential concerns:
    - Filling the wash station container: how can they make sure they always have water for washing? Who will be responsible for filling the wash station container? When will they do this during the day? Suggest collecting more water and dedicating some water to the wash station each time water is collected.
    - Not taking the wash station container to the water point: carrying it can break the tap.
    - Avoiding wastage: close the tap whilst lathering hands or whilst rubbing soap on faces to avoid wasting water.

Soap

1. Turn the flipchart – WASH STATION 'SOAP' images.
2. Ask the group to describe the images.
3. **Summarise: a dedicated soap is kept in a soap dish at the wash station.**
4. Ask participants if they have any suggestions or concerns about keeping soap at the wash station.
5. **Guide the discussion** to cover the following potential concerns:
    a. Ability to dedicate soap and keep it at the wash station: put the soap we gave them at the community event in the soap dish as it protects the soap from rain

and getting dirty. Could bring soap inside at night along with the wash station container and take it out again in the morning.
   b. Lack of soap: Could also make soapy water and keep it at the wash station.
   c. Children wasting soap: young children could be supervised?

Night

1. Turn the flipchart – WASH STATION 'NIGHT' image.
2. Ask the group to describe the image.
3. **Summarise: at night, if there is no fence around the station, it might be preferable to take the wash station container and the soap and the soap dish inside the house. Someone should be responsible for bringing the station, soap dish and soap outside every morning.**
4. Ask participants if they have any suggestions or concerns about bringing their materials inside during the night and outside during the day.
5. Guide the discussion to cover the following potential concerns:
   a. Security: The wash station container could be secured outside or brought inside at the end of the day.
   b. Bringing it back outside in the morning: If people want to bring it inside, who will be responsible for moving it each morning and evening? E.g. the person who wakes first in the morning could bring the materials outside when they wash.

Complete station

1. Turn the flipchart – WASH STATION 'COMPLETE' image.
2. Say that this image summarises all the points you have just discussed about wash stations. Go through each point.
3. Ask participants whether they have any other questions or concerns about how to set up their wash station when they get it home.
4. Remind participants that all this information is summarised on the wash station flyer they received at the distribution. Suggest that they use the flyer to remember the important features of the wash station stand. Suggest that neighbours help each other.

Introduce the Testimonials

1. Tell participants that we will now show them some ways members of their own community have built their stations and have overcome barriers to use their wash station to help their family maintain *Faces of Dignity.*

ACTIVITY 5: TESTIMONIALS

Purpose

- Learn from neighbours' and role models' experiences about building, using and maintaining a face washing station.
- Functioning wash station is consistently available and accessible.
- Perceive the wash station to be useful.
- Create the impression that important community members are already using wash stations to practice the target behaviour.

What to do

1. Turn the flipchart – TESTIMONIALS page.
2. Start playing the voice-recorded Testimonials on a tablet or cell phone. Ensure everything can hear what is being said. If requested by the participants, play the Testimonials again. For content of the Testimonials, **refer to Appendix A**.
3. Discuss the testimonials briefly with the participants.
4. Ask if anyone has any comments on anything they have heard. Answer any questions.

5. Ask participants, especially fathers, if they now feel they have all the support and the information they need to set up a wash station to make it easier for their family to wash their faces 3x a day with soap and to maintain their *Faces of Dignity*.
6. Specify that:
    a. We advise them to put the body soap they got at the Community Event inside the soap dish they will be given at the end of this event.
    b. This is a dedicated soap for face washing which should be used at the station. If it has already run out, suggest that they buy more soap when they can.
    c. Next time we meet we will discuss how to make soapy water so that soap lasts even longer.
7. Conclude the activity informing participants that they will be asked to give their own testimonials at our next forum a week from now.

NB. This activity will be refined during pilot-testing. Testimonials might be video or audio-recorded according to what is feasible. If recording Testimonials is not feasible, this activity might also be dropped from Family Forum 1.

ACTIVITY 6: DIGNIFIED DAY PLEDGE

Purpose

- Engaging, accessible, appropriate, strategically placed washing prompts/cues are visible.
- Perceive face washing to be important for maintaining dignity of self and family.
- Perceive soap as important to use each time faces are washed.
- Perceive effectively washing face at least three times per day as important, all year around.
- Accept responsibility for hygiene of young children.
- Perceive an expectation from husbands & neighbours to maintain clean faces of self and family.
- Perceive an expectation from parents to maintain clean face.

What to do

1. Turn the flipchart – DIGNIFIED DAY PLEDGE page.
2. Before starting the Dignified Day activity, ask children to join back the forum. Congratulate children on their drawings and take back the wax crayons. Explain children that they will receive their wax crayon at the end of the event the week after.
3. Take Caltu's puppet in your hand and animate the activity with the puppet.

A Dignified Day

1. Ask participants to raise their hands if they agree that being dignified and ensuring that our children are dignified is really important, that it is valued in the society, and gives them respect from the other members of their community.
2. Ask people to raise their hands if they:
    a. Value their dignity.
    b. Value the dignity of their children.
    c. Believe that children represent their family everywhere they go, at all time, i.e. their faces are the faces of the family.
3. Tell participants that we all know that the way we live each day is important for upholding our dignity and the dignity of our children.
4. Ask the group for examples of important behaviours that they do each day to maintain their *Faces of Dignity*.
5. Turn the flipchart – POSTER – **"DO YOU ENSURE YOUR FAMILY'S DAYS ARE DIGNIFIED?"**
6. Go through the poster.
7. Ask participants if they agree that these activities should be performed daily to maintain their family's *Faces of Dignity*.

8. Remind participants that becoming *Faces of Dignity* will also contribute to enhancing their Community's dignity. Ask participants if they agree that becoming *Faces of Dignity* will enhance their Community's dignity.

Pledge

1. Bring out the **Dignified Day poster**, one per family.
2. Ask each family to hold hands.
3. Ask the parents to repeat the following pledge after you (pause after each sentence). Tell people that they should not repeat any statements that are not relevant to them e.g. if they have no children:

> **I pledge to build a stand for this wash station.**
>
> **I pledge to ensure that there is always soap available at this station so that my family can maintain their *Faces of Dignity.***
>
> **I pledge to help my pre-school age children to wash their faces and hands with soap three times a day: first thing in the morning and before they eat lunch and dinner.**
>
> **I pledge to set a good example for my children by washing my own face and hands with soap three times a day.**
>
> **I pledge to help my family maintain *Faces of Dignity* and enhance my Community's dignity.**

4. Ask any school age children present to repeat the following pledge after "the puppet":

> **I pledge to always wash my face and hands with soap three times a day: first thing in the morning and before lunch and dinner.**
>
> **I will do this so that I always have a *Face of Dignity* and can proudly represent my family.**

5. Clap and congratulate everyone on their pledges and for being valued members of their community.
6. Encourage participants to decorate their wash stations to personalise them.
7. Advise participants to put up the poster inside the house somewhere they will be able to see it each day.
8. Ask whether anyone in the audience has a cell phone. If anyone has one, suggest this person than setting up an alarm three times a day can help them to remind about face washing. Propose your help to set up the alarm on the phone at agreed times. Suggest people with a phone informing their neighbours without a phone each time their alarm rings to remind them about face washing.

ACTIVITY 7: CONCLUSION

What to do

1. Turn the flipchart – CONCLUSION page.
2. Using the puppet, tell participants that we are now at the end of this family forum. Thank them for their participation.
3. Ask participants if they have any question or concerns.

4. Tell participants that you will visit them again in a week to see how they are doing, to answer any questions, and to check on their *Faces of Dignity*.
5. Remind participants that their Community cannot be dignified if all its members are not dignified. Their community's dignity will be publicly recognized and rewarded only if everyone in the community has a *Face of Dignity*.
6. Recommend that all family members attend the second family forum as there will be activities designed for everyone.
7. Check that every family leaves with: **a laminated "Dignified Day" poster and 2 small nails**.
8. Check that families who missed the Community Event receive all their materials (wash station, 2 soaps, soap dish and wash station flyer).
   NB. This will be organised locally by the HVs and/or Berhan enumerators.
9. Play the Dignity Song on a cell phone or any other device while participants are leaving the forum.
10. Put the puppet back into its cover.
11. Wash your hands with water and soap or alcohol-based sanitizer.

End of Family Forum 1.

Report Household Head Names of households who missed the forum on the Family Forum 1 Follow-up Visits Form before ending the event.
These households should be invited to join other Family Fora 1 held with other households in their cluster.
If they cannot join another session, individual follow-up with these families will be organised at their home.
Refer to Appendix B for details of the content to cover during this visit.

Appendices

Appendix A. "Content of Testimonials"

Testimonial 1: **"Building a wash station"** | Interviewee: ideally, a man

Question: Why did you decide to build a wash station stand?

Answer: I built a wash station stand for my wash station so my family and I could wash our faces with soap easily so we have *Faces of Dignity* in the community and.

Question: How did you decide on the location and the height of your station?

Answer: I built my wash station stand near to the entrance of our house so the wash station is protected from the sun and easily moveable at night, when I bring the wash station container, the soap dish and the soap inside. I built the stand like this so my older children can use it easily, but my younger children, who are supported by my wife or myself to wash, cannot play with the soap and make it dirty, or with the tap and waste the water.

Question: Was it hard to build a wash station stand?

Answer: Not at all! *They should explain what they did. E.g.* It was easy and quick to build the stand with some wood I collected. I made a strong structure and then put the container on top of it. I dedicated a baldy to collect the wastewater, and added a dedicated body soap which I put in the soap dish near the container. It was done in less than 2 hours.

Testimonial 2: **"Maintaining a wash station"** | Interviewee(s): a man or a couple

Question: Have you set some roles for taking care of the washing station in your house?

Answer (man): Yes, we have. *They should explain what they have done. E.g.* At first it was not easy to get used to the station, moving the wash station container, filling up the water, always having a dedicated soap. That's why my wife and I decided to set some roles so we don't forget anything. I take care of the materials, bring the station in and out in the morning and at night. My wife is responsible for water and soap and making sure the station is always secured during the day. For instance, that the children do not play with the tap.

Question: Is your wash station always staying outside?

Answer (man): *They should explain what they have done. E.g.* At first, we thought about leaving the washing station outside at all time, but we had nothing good enough to secure it from wild animals or to make sure we do not lose everything overnight. So, our wash station is brought inside our home at night. Because I am often the one waking up and going outside first in the morning, I am the one responsible for putting the wash station container and the soap and soap dish on the washing station stand every morning. At night, after the evening wash, I am also the one responsible for bringing the wash station container and the soap dish inside the house when I close the door. I never forget to put the station outside in the morning or inside at night because the wash station stand is visible from the door of our house.

Question: How do you make sure water is always available at the station?

Answer (woman): *They should explain what they have done. E.g.* I am usually the one responsible to fetch water every morning. Since we have the station, I have dedicated the water of one of our jerry cans to fill the washing station. Like this, that is really easy. I don't have to worry about having enough water for washing 3x a day. Often, there is even some water left inside the washing station container at the end of the day for the day after.

Question: How do you make sure a dedicated body soap is always available at the station?

Answer (woman): *They should explain what they have done. E.g.* Each time I notice that the soap is going to run out soon, I ask my husband for money to buy a dedicated soap for face and body washing. He always gives me money or buys the soap himself. When possible, I buy two soaps at the same time to make sure we always have a spare soap. We thought it would be difficult to keep soap outside because we are not used to do this, but it works and helps us remember to use soap when we wash. That is how we are maintaining our washing station to make sure our family has *Faces of Dignity*.

<u>Testimonial 3:</u> **“Routine use of a wash station”** | Interviewee: ideally, a female primary caregiver

Question: How do you remember to wash your family’s faces three times a day?

Answer: In the morning, we used to wash our faces before having the station, so that is easy. But now we have the station, I never forget about using the soap and supporting my younger children so I can wash their eyes and noses thoroughly. I supervise the older ones too. Before lunch, no one has ever forgotten about face washing with soap, as we are also washing our hands before eating at the wash station. We have associated washing hands and washing faces. That is a good reminder. In the evening, we all wash our faces and hands before dinner, our children even remind us about it. In our family, everyone knows about face washing 3x a day with soap, morning, before lunch and before dinner.

Question: What do you like about using the washing station?

Answer: The washing station has made our lives so much easier and we have saved a lot of water. Now we have it, it is so much simpler to maintain our family’s *Face of Dignity* by washing our children’s faces three times a day with soap. With our wash station and soap always nearby, it is so simple. In my family, we have *Faces of Dignity*, do you?”

Appendix B. "Follow-up with any households that do not attend the Family Forum 1"

HVs should do their best to ensure all households attend Family Forum 1. If any families do not attend, they will need to be followed up at home before Family Forum 2 by the Berhan Activators.
If the household missed the Community Event as well, the wash station, soap dish, soaps (x2) and wash station flyer should be brought to the follow-up visit.
The main activities of the forum should be gone through with the family, with modifications detailed below. The poster giveaway should be provided.

Setting

- Sit with the female primary caregiver and any family member present at home. Make sure the participants are comfortable, i.e. they should be sat in the shade.
- All materials required for the visit are organised and ready to be used.

Materials

**General**	– A facemask, alcohol-based sanitizer, sealable plastic bag for mask disposal		
**Transmission**	– Trachoma Transmission diagram	**Face wipe Emo-Demo**	– 2 wipes
**Transmission Routes**			
**Dignified Day Pledge**	– Dignified Day poster<br>– 2 small nails		

Activities

Introduction

1. Wash your hands with soap or alcohol-based sanitiser and ensure you are wearing a facemask before entering the compound. Explain why you are wearing a facemask. If possible, stay outside to do the visit.
2. Greet the female primary caregiver / female adult. If they are not home **do not proceed: wait or return later.**
3. Say that the visit is short (15 mins) and ask her to gather any family members who are close by.
4. Tell the household you have come because they missed the first Family Forum and you would like to share with them what was discussed at the forum. Ask why they didn't attend.
5. Ask if they have any questions before you start your visit.

Trachoma Transmission Routes

1. Ask what they learnt about how trachoma can get from one eye to another eye at the Community Event (should mention flies and hands). Correct them if needed and congratulate them on their answers.
2. Show the **Trachoma Transmission Diagram** and explain that you are going to talk about transmission so that everyone understands why it is so important to get rid of discharge. Say that trachoma is a small germ and is carried in discharge that we can often see on people's faces.
3. Describe the three routes of trachoma transmission, e.g. flies, hands, clothes. Advise the caregiver to wipe her children's discharge with her hand only and to directly wash her hands with soap to remove the discharge from her hands.
4. Ask them to explain what they have learnt about how trachoma can be transmitted and congratulate them on their understanding.
5. Ask what they can do to get rid of the discharge carrying trachoma and maintain their children's dignity / their own dignity. They should answer face and hand washing. If not, probe for what was done in the community event.

6. Say that faces need to be thoroughly washed to get rid of discharge, especially around the eyes and nose. For families with children, add that pre-school children need to be helped so they can wash well.

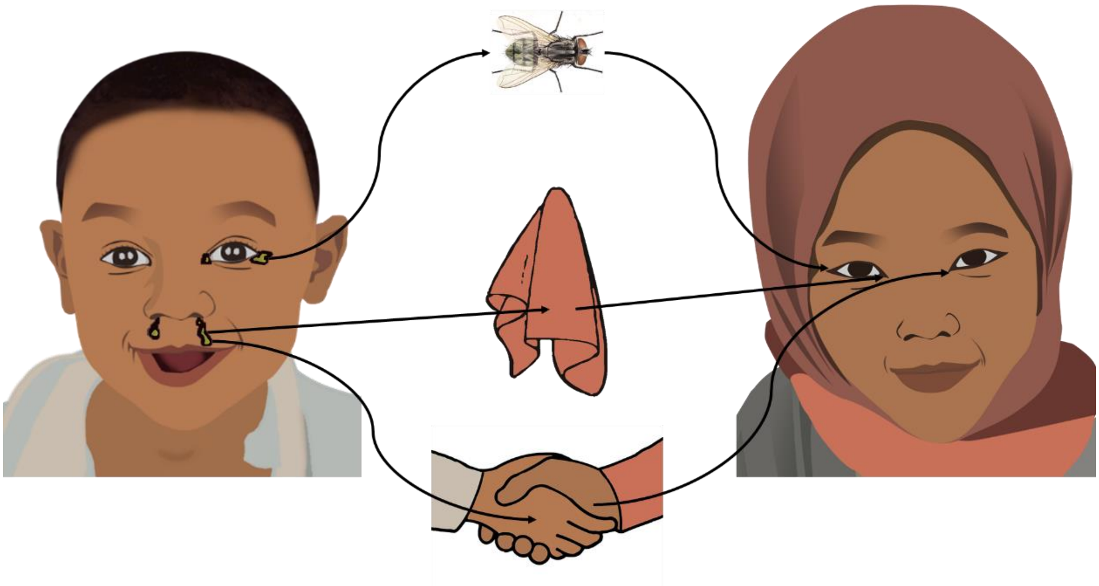

Face Wipe Emo-Demo

NB: The activator should have put foundation on their face before the visit.

1. Ask if they think that they are always able to see all the dirt and discharge on a person's face.
2. Ask if they think that "you" (the activator) have a clean face.
3. Explain that you are going to use a wipe to confirm what they have just said. Explain that the wipe is just a cloth that is a bit wet.
4. The activator should wipe one side of his/her face and show the dirty wipe to the family. Is this a surprise?
5. Say that faces can look clean even when they are not. That is the case for all of us, but especially for children who are spending a lot of time outside, playing with friends, etc.
6. Ask what they could do to make sure their faces/ children's faces are never dirty and undignified when they appear clean (i.e. wash face with water and soap). **Let people answer*.* Probe the family for what the family did in the community event.
7. Remind the household that face washing with soap is important for true cleanliness and dignity.
8. Ask if they agree that soap is the only way to achieve *Faces of Dignity*, that they value their dignity and the dignity of their children, and that they want to wash faces with soap regularly so their whole family have *Faces of Dignity*.

Wash station

1. Tell the household that to keep their *Faces of Dignity*, face washing with water and soap is key, but that it is not always easy to gather all the materials and the soap to wash.
2. Ask the female primary caregiver to bring forward the wash station, the soap dish with the soap and the wash station flyer they received after the community event. Ask if they have already started using their wash station (or used one before, e.g. in town) and what they like about it.
3. Say that you would like to quickly discuss how to construct a stand for their wash station and how the station should be used.
4. Use the **wash station flyer** to describe how to build and use the station. Focus on a male household head if he is present:

a. **Location and Structure:** a 'good' location is outside, close to the home and in the shade. The wash station and soap dish are on a built, sturdy wooden structure that is secured and cannot be knocked over by children or animals.
b. **Height:** if the household has children, very young children cannot reach the tap and soap without help, but the station should be accessible to all other family members.
c. **Drainage:** a bowl or stones/sand are placed under the tap to prevent the ground from becoming muddy.
d. **Water:** someone is responsible for filling the wash station container with water so there is always water available for use. The wash station container should not be taken to the water, the tap is fragile. The tap should be closed whilst lathering the hands or rubbing soap on faces.
e. **Soap:** a dedicated soap is kept in a soap dish at the wash station. The soap is always clean and available for body washing when it is needed.
f. **Night:** at night, if there is no fence around the station, it might be preferable to take the wash station container and the soap and the soap dish inside the house. Someone should be responsible for bringing the station, soap dish and soap outside every morning.

5. Ask whether they have any question or concerns about how to set up their wash station.

Dignified Day Pledge

1. Ask the household if they value their dignity, the dignity of their children (if they have any), and if they believe that children represent their family everywhere they go, at all time, i.e. their faces are the faces of the family.
2. Ask the household if they also value their community and would like to contribute to their community's dignity.
3. Say that we all know that the way we live each day is important for upholding our dignity and the dignity of our children.
4. Show the poster "Do you ensure your family's Days are Dignified?" and go through the poster.
5. Ask the household if they agree that these activities should be performed daily to maintain their family's *Faces of Dignity* and enhance their community's dignity
6. Ask the family members to repeat the following pledge after you (pause after each sentence). Do not read out statements referring to children if there are no children in the household:
   a. I promise to build a stand for this wash station and make sure there is always water and soap there so that my family can maintain their *Faces of Dignity* and we can contribute to enhancing our community's dignity.
   b. I pledge to help my pre-school age children to wash their faces and hands with soap three times a day: first thing in the morning and before they eat lunch and dinner.
7. Congratulate the household and encourage them to decorate their wash station to personalise it.
8. Advise them to put up the poster inside the house somewhere they will be able to see it each day.

Conclusion and Giveaways

1. Tell the household that this is the end of the visit. Ask if they have any question or concerns. Thank them for their time.
2. Tell the household that the second Family Forum will take place in a week and that you recommend that all family members should do their best to attend as there will be activities for everyone. Discuss if they think it will be difficult to attend.
3. Check that the household has received: **a laminated "Dignified Day" poster, and 2 small nails.**

4. Wash your hands with water and soap or alcohol-based sanitize after leaving the compound. Ensure you are safely disposing your facemask in a sealable plastic bag at the end of the morning visits or at the end of the day.

EVENT 3 – FAMILY FORUM 2
ACTIVATOR MANUAL

**Purpose**	Continue to build skills and motivation to practice face washing. Overcome early barriers related to wash station construction and use. Emphasize the need to wash faces with soap three times a day now that everyone has a functioning wash station.
**Responsible Parties**	1 trained Activator + 1 trained Health Volunteer (2 HVs will assist the Activator in each cluster to spread the work load)
**Participants**	All household members living within the 'yolk' of an intervention cluster who attended Family Forum 1 – Split into groups of 5 households.
**Location**	A HH compound (selected in advance when HHs are informed the time and date for the forum)
**Duration**	1h30
**Timing**	Workdays (except Friday morning), 9am to 1pm and after 3pm

Preparation

Day before the event

- Mobilise HVs to invite the same 5 neighbouring households as for Family Forum 1 (including households who joined other groups because they missed their session).
- HV to ask the host of the 1st forum if they will also host Family Forum 2. Ensure the selected household has constructed a wash station stand or provide encouragement so they do so before the event.
- Communicate time and location of the event to each participant household.
- Inform each household that the whole family should participate if possible.

Setting

- Set up the forum in a similar way to Family Forum 1, ensuring at least a 2-meter physical distance between members from different households. Tweak the layout as necessary to enhance the experience of the group based on your learnings from running the previous forum.

Materials

General

- COVID-19 preventive materials: cleaning solution in a spray (x1), alcohol-based sanitiser, facemasks (for Activator and HV), sealable plastic bag to dispose masks
- Flipchart
- Caltu's puppet in its cover
- Pen
- Notebook
- Cell phone or device to play the Dignity Song

Wash-Along

- Wash station containers with tap (one borrowed from the HV, one from the host household) + 2 collectors + 3 soap dishes + 3 soaps + 1 stool borrowed from the host household
- One 20L jerrycan of water (revise quantity based on experience running this event)

**A "Dignified Day"**	– 7 Velcro flashcards
**Barriers and** activity) **Solutions**	– 2 jugs (+ 2 collectors + 1 soap + 1 soap dish + jerrycan of water from the Wash-Along<br>– 1 coffee tray + 1 jebena + 6 coffee cups<br>– Velcro extra people to stick on the Dry season infographics<br>– 6 empty plastic water bottles 1L<br>– 5 strings for participants to attach their soapy water bottle on their wash station<br>– 3 nails<br>– 1 body soap (to make soapy water + to be cut)
**Soaps giveaway**	– 5 body soaps

Set up

Day of the family forum

- Do you have all materials required for all activities?
- Is equipment working?

Activities

Activity 1: Introduction

Purpose

- To provide introduction to the forum.
- To address any concerns or questions arising from the first Family Forum.

What to do

1. Install the flipchart – FAMILY FORUM 2 page. Install Caltu's puppet next to the flipchart on its cover.
2. Play the Dignity Song on a cell phone or any other device while participants are arriving.
3. Advice community members to sit with their household members and respect a physical distance of at least 2m with other households.
4. Greet the participants and welcome them to the forum.
5. Complete attendance sheet.
6. Ask the participants whether they have any question concerning the first family forum or the programme.
7. Tell the group that the event should not take more than 1h30. Giveaways for each household will be given at the end, so they should stay until the end of the forum.

Activity 2: Live testimonials

Purpose

- The primary purpose of this activity is to identify and overcome barriers to face washing with soap x3 a day associated with wash station construction and use.
- Can construct a functional wash station.
- Can maintain the functioning of a constructed wash station.
- Functioning wash station is consistently available and accessible.
- Perceive the wash station to be useful.
- Self-efficacy relating to construction of functional wash station.
- Self-efficacy relating to maintenance of wash station.
- Can troubleshoot complaints of young children during washing.

What to do

1. Turn the flipchart – LIVE TESTIMONIALS page.
2. Introduce the activity.

Building the station

1. Lead a discussion around the following topics to explore barriers and solutions to the construction of a wash station stand:
2. To everyone ask:
    a. Have you managed to build a wash station stand? → Participants raise their hands if they have built a stand. Count the number of hands.
    b. How do you all feel about your wash station? Should we take it away again or is it useful?
3. To the family hosting the forum ask:
    a. Can you show us all your wash station?
    b. Are you pleased with it? Why?
    c. Did you have any problems building it?
    d. Have you had any problems using it?
4. To the other households ask:

    a. Have you built your wash station stands differently to this one? Ask people to volunteer what they have done.
    b. Have you had any issues building your stands?
5. Discuss solutions to any problems that are raised.

Using the station

1. Lead a discussion around the following topics to explore barriers and solutions to the use of the wash station for face washing with soap 3x a day:
2. <u>To the children</u>, ask:
    a. Are you using the wash station? If so, have you found it easier to wash your faces with soap now that you are using the wash station?
    b. Have you felt any difference on your skin now you are washing your face with soap regularly?
        i. If children or adults mention skin being dry:
            1. Tell them that this is nothing to be worried about and that is likely to be temporary.
            2. Advise them to use a body soap (which is softer on the skin) as much as possible.
            3. If having dry skin becomes an issue, suggest applying a moisturizing cream or lotion on their children's faces with clean hands (i.e. primary caregiver should wash hands with soap before applying the cream). That cannot impede their *Faces of Dignity*.
        ii. If no one mentions skin being dry:
            1. Inform children and adults that this could happen and provide similar advice (points 1, 2, 3 above).
3. <u>To the adults</u>, ask:
    a. Are you using the wash station to wash your children's faces?
    b. Is it easy to use the wash station to wash your children's faces at all three times of day (morning, before lunch and before dinner), or is it not practical to use it at a particular time?
    c. Has anyone found it hard to make sure that there is always water in the container? Has anyone found any solutions? E.g. filling it as soon as water is collected.
    d. What about making sure there is always soap in the soap dish and that this is kept at the wash station? Who has managed to do this?
    e. Who is moving their wash station inside at the end of the day? What is everyone else doing and why?
    f. Have you been remembering to wash your children's faces three times a day, in the morning, before lunch and in the evening before dinner? What solutions have you found to help you to do this? E.g. setting an alarm, linking to the daily routine.
        i. Ask participants whether any of them owning a phone was able to set up an alarm 3 times a day to remind themselves and their neighbours about face washing.
            1. If yes, ask these participants to share their experience and to describe how useful the alarm is.
            2. If no, ask whether they have a phone and would like your guidance to set up an alarm 3 times a day.
4. Discuss solutions to any problems that are raised.
5. Ask participants whether they have any remaining questions or comments related to the wash station or its use.

Introduce the Wash Along

> **"Thank you all for sharing your experiences building and using your wash stations. We hope you have been able to get some answers to your questions and to learn from the experience of your neighbours to improve your own practice. We would now like to wash our faces all together using a wash station".**

ACTIVITY 3: WASH ALONG

Purpose

- Can consistently wash own hands and face with soap using an effective and efficient technique.
- Can consistently wash young children's hands and face with soap using an effective and efficient technique.
- Perceive soap as important to use each time faces are washed.
- Accept responsibility for hygiene of young children.
- Self-efficacy relating to effective and efficient washing technique for self and pre-school children.
- Perceive an expectation from neighbours to maintain clean faces and dignity of self and family.

What to do

1. Turn the flipchart – WASH ALONG page.
2. Set up materials for the Wash Along:
   a. Put the HV's wash station on a plastic chair borrowed from the host household, and ask the host household to borrow their wash station. Put 1 collector underneath each station. Bring forward the 3 soaps in soap dishes.
   b. Fill the 2 wash stations with 5L of water.
   c. Disinfect the tap of each wash station by spraying cleaning solution.
   d. Take Caltu's puppet in your hand and animate the rest of the activity with the puppet.

Face washing demonstration

1. Ask a volunteer mother to wash both her face and her pre-school child's face using a wash station.
2. Before she washes, Caltu's puppet asks the rest of the group the following questions:
   a. How can the mother make sure she removes all visible and invisible discharge?
      i. Briefly discuss wiping: remind participants that wiping should be done with hands and hands should be washed with soap immediately after to remove discharge from the hands.
   b. How many times a day should we wash our children's faces with soap to ensure they have *Faces of Dignity*? When?
   c. Whose *Faces of Dignity* are the most important? (i.e. pre-school children as they are the ones having discharge and the one going everywhere in the community and representing the family at all times)
3. Ask the group to observe as she washes.
4. After she has washed the child, clap and congratulate the mother on being a role model for her children to copy her behaviour and for supporting her child to have a *Face of Dignity*. Say that neighbours will know what she is doing and she is representing her family well in the community.
5. Put Caltu's puppet back on its cover.

Closing the tap to avoid wasting water

If the mother closed the tap whilst scrubbing her child's face, congratulate the mother and remind the group how important this is to avoid wasting water.
If the mother did not close the tap whilst scrubbing her child's face, remind the group how important this is to avoid wasting water, remind them to do so for every wash and to teach their children to do so too when there are old enough to wash themselves. Say that washing as a family means they can control how much water (and soap) the children use for face washing.

Hand washing with soap

If the mother washed the child's hands, congratulate the mother and remind the group how important this is.
If the mother did not wash the child's hands, ask her to do so now and remind the group that face and hand washing with soap should always go together unless someone is washing their own face with their hands. Hands should always be washed with soap after wiping discharge.

1. Invite each household to come one by one at the HV's wash station and wash children's face (e.g. washed or supervised by parents or elder siblings). If possible and according to time, every other household member (including caregiver and man/household head) should wash their face at the station. Disinfect the tap of the station between each household by spraying cleaning solution. Ask only the host's family to use their wash station.
2. Remind participants if pre-school children should be prioritized for face washing, everybody should do it to set a good example and to help children do it.
3. Remind adults to support younger children by washing their faces for them.
4. Play the Dignity Song on a cell phone or any other device while all participants are washing faces.
5. Congratulate the group when everyone has finished washing (everyone should clap).
6. Say that washing our family's faces 3x a day with soap, washing our eyes and nose thoroughly, and making sure the younger ones are prioritised and supported every time, will help us achieve *Faces of Dignity*, faces that will make us dignified and respected people.
7. Say that our neighbours notice when we have *Faces of Dignity* and they know we are contributing to enhance the community's dignity, which depends on each individual
8. Conclude the activity by encouraging the group to continue using their wash stations to act with dignity by making sure soap and water are always available for body washing.

Activity 4: "A Dignified Day"

Purpose

- Perceive face washing to be important for maintaining dignity of self and family.
- Perceive effectively washing face at least three times per day as important, all year around.
- Accept responsibility for hygiene of young children.
- Perceive an expectation from husbands and neighbours to maintain clean faces of self and family.

What to do

1. Turn the flipchart – A Dignified Day page.
2. Take Caltu's puppet in your hand and animate the activity with the puppet.
3. Remind participants that they were given a poster in the last forum.
    a. Ask where they have all put their posters.
4. Ask the children:

a. Do you remember the dignified activities on the poster that help a family have a dignified day?
    i. Each time a child gives a right answer, put the corresponding flashcards up on the Velcro flipchart using the puppet.
    ii. It they cannot remember all the activities, show them any outstanding flashcards, ask the group (children first, adults if help is required) what the image represents, and put the flashcard on the flipchart.

5. Ask the whole group to raise their hands if they intend to keep doing these activities so that their families can continue to lead dignified lives and contribute to enhancing the dignity of their community.
6. Conclude the activity by reminding the group that these are small things we can do to help our family's gain and maintain *Faces of Dignity* and contribute to enhancing the community's dignity.
7. Put Caltu's puppet back on its cover.

Activity 5: Barriers to Face washing & Solutions

Purpose

- Can troubleshoot reduced access to water in dry season and continue to prioritise water for hand and face washing with soap.
- Willing to prioritise water for face washing, all year around.
- Self efficacy relating to prioritisation of water for face washing, all year around.

What to do

1. Turn the flipchart – Barriers to Face washing and Solutions page.
2. Before starting the Barriers and Solutions activity, propose to children (aged 2 to 7 or 8) to colour in a drawing of the puppet explaining about face washing. Tell the children they will be able to keep their drawing to remind them about when they should wash their faces to become *Faces of Dignity*. Put the puppet in a visible place. Give one drawing to each child and a few wax crayons disinfected with the cleaning solution.
3. Ask participants whether they feel they might have some issues washing their faces 3x a day with soap using the wash station in the next days, weeks or even months. Discuss.
4. Tell the group that we would like to talk in a bit more detail about some of the issues that they have raised or that other people living near them have found to be problems.

Lack of Water

1. Turn the flipchart – Lack of Water page.
2. The first issue is lack of water, especially during the dry season, and how this affects people's ability to keep a continuous supply of water in their wash stations.

Water perception demonstration

1. Ask participants whether they stop drinking coffee during the dry season.
2. Ask how many times a day they can prepare and drink coffee (at maximum).
3. Agree with participants that they drink coffee all year round and that each time they prepare coffee they use water to clean the materials (i.e. tray, cups, jabena) and they use water to make coffee.
4. Say that you would like to do a quick demonstration to show them something interesting.
5. Do the water perception demonstration.

Water perception demonstration

1. Both activators fill a jug with water and take a water collector.
2. Show the full jugs to participants.
3. The Activator washes his/her face with soap over a collector (trying to minimise water used).
4. The HV washes a coffee tray, 6 coffee cups and a jebena over a collector (washing thoroughly without giving the impression of overusing water).
5. Show the group the difference in the amount of water in both collectors and what is left in both jugs.

6. Ask participants to raise their hands if they agree that preparing and drinking coffee consumes more water than face washing.
7. Get them to agree that they drink coffee several times a day and therefore they also have enough water for face washing 3x a day.
8. Tell participants that water is obviously scarcer during the dry season and that we should teach our children to use it carefully and to turn off the tap on the wash station when water is not needed.

Dry season infographic

1. Turn the flipchart – FACE WASHING IN THE DRY SEASON page.
2. Tell participants that you realise some of them may not yet be convinced that they will have enough water for their whole family to wash their faces with soap x 3 a day, all year round.
3. Say that we watched people in a nearby community using water in their homes in the dry season, rainy season and at harvest time. We want to show them what we found.
4. Discuss the Dry Season Infographic on the flipchart:
   a. Explain the infographic and what it shows.
   b. Ask if people are surprised by what is shown on the infographic (that people wash their faces a lot more in the dry season than at other times of year). Discuss.
   c. Get the group to conclude that they have enough water in their homes to wash faces 3x a day, even in the dry season, especially now that they have a wash station which makes it easier to wash and uses less water.
   d. Stick "extra people" on the infographic to show what we need to change to achieve *Faces of Dignity* all year round (i.e. extra face washes at midday and in the evening).
   e. Get the group to agree that they want to do this and can do this, to keep their *Faces of Dignity*.

Lack of Soap

1. Turn the flipchart – LACK OF SOAP page.
2. Ask participants to raise their hands if they feel that lack of soap is going to be an issue for them to practice face washing 3x a day with soap all year round.
3. Ask participants, regardless of whether they raised their hands, whether they have already found a way to make sure they always have soap for face and hand washing in their home. Discuss.
4. Thank participants for sharing their solutions. Explain that you would also like to share some solutions that communities nearby have come up with.

Soapy water demonstration

1. Explain that we can wash faces with soapy water if we do not have bar soap.
2. Ask anyone who has done this before to share their experiences
3. Demonstrate how to create soapy water using a plastic bottle.

Soapy water demonstration

1. Disinfect each empty plastic water bottle by spraying cleaning solution.
2. **Give every household an empty plastic water bottle of 1L.**
3. Put a small piece of soap in each bottle. Tell them that they will receive the rest of their soap bar at the end of the event.
4. Fill each bottle with water from the jerrycan.
5. Ask participants to shake their plastic bottle many times.
   →Tell them to stop here and leave it to dissolve for a day and finish the rest later.

6. Activator demonstrates how to finish making the soapy water after they have left the soap to dissolve
7. Shake the bottle vigorously again.
8. Check the water creates a good lather, if not, add more soap and shake again.
9. Make a hole in the lid of each plastic bottle using a nail.
10. Use the soapy water for face washing and use clean water to rinse. At the end of the demonstration, the activator reports how it feels to use the soapy water **"Oh, I am definitely using soap and not just water, I can smell it, and it feels different."**

4. Ask participants whether they feel they could do the same themselves.
5. Explain how the soapy water bottle can be attached using a string to the wash station. **Give a string to each household.**
6. Tell the group that soapy water can last a lot longer than the same amount of bar soap.
7. Ask whether anyone has any questions or other ideas about making soapy water.

Prioritising soap use for face washing instead of laundry, i.e. cutting soap

1. Explain that you know that soap is used for many things other than face washing and it can be hard to keep soap just for body washing.
2. Say that some people in nearby communities cut their soap in half so that soap can be kept in the soap dish and used just for face washing.
3. Ask anyone who has done this before to share their experiences.
4. Ask whether anyone has any questions or other ideas about dedicating soap for body washing.
5. Ask participants whether they feel they could do the same themselves.

Prioritising pre-school children for soap use

1. Tell participants to imagine that, despite using all the methods above to have soap, there is one day where there is soap for only one person in their house.
2. Ask participants whose *Face of Dignity* is the most important and who should be prioritised to get the soap. Discuss.
   a. If participants did not mention pre-school children, remind them that pre-school children are the ones having most discharge on their faces and the ones going around the community all day playing with their friends and going to neighbour's homes. Children are the faces of the family and their *Faces of Dignity* should be prioritised at all time.
3. Say that in life we always find a way to do something when it is important to us, and the *Faces of Dignity* of our family is really important so we know that everyone will work hard to make sure they always have soap.

Washing with water only

1. Say that despite all these solutions which can help to always have soap, it is possible that they might lack soap sometimes (e.g. have forgotten to buy a new one).
2. Advise participants to keep washing with water 3 times a day anyway. Tell them that they should not break their habit and feel discouraged. They should rather keep washing with water and reintroduce soap as soon as possible.

Lack of Attention

1. Turn the flipchart – LACK OF TIME AND FORGETFULNESS page.
2. Ask participants to raise their hands if they feel that lack of time or forgetfulness is likely to prevent them from practising face washing with soap x3 a day, all year round.

Forgetfulness

1. Explain that we often remember to do something when we see something in our home or environment that is connected to the activity we want to do.
2. Ask people how they remember to wash their hands before eating. Discuss.
3. Conclude that we can be reminded by lots of different things, such as the time of day, activities that always happen before or after, other people doing it or telling us to do it.
4. Say that the same can be true for face washing. We can help remind each other and we can always wash at fixed times of day, but we can also use visual reminders that make us think of face washing when we see them e.g. the "Dignified Day" poster, the wash station and soap dish, the soapy water bottle, etc.
5. If anyone has a phone, remind they could set alarms 3 times a day on their cell phone to help them to remember.
6. Ask participants whether they have any ideas of other things that could help them remember to wash their faces and their children's faces with soap 3x a day.

Lack of Time

Infographics

1. Explain to participants that many of their neighbours who complained about lack of time for face washing in the past, are now reporting that the face washing station has considerably changed the time it takes for face washing. It is really quick now.
2. Ask participants to raise their hands if they believe that the wash station makes it quicker to face wash.
3. Ask participants to raise their hands if they believe that lack of time will still be an issue for face washing at certain times during the year.
4. Turn the flipchart – PIE CHART 1 page.
    a. Show and explain the infographic
    b. Discuss the fact that face washing does not take much time compared to all the other activities they do every day
5. Turn the flipchart – PIE CHART 2 page.
    a. Show and explain the infographic
    b. Discuss that they do a lot of activities every day. The activities they choose to do are ones that are important. They can fit face washing in if they choose to prioritise it. Isn't it important to give our children the gift of a *Face of Dignity*?
    c. Ask the group whether they can make sure there is always a bit of time for face washing.

Other solutions

1. Discuss: sharing the responsibility of face washing with other adults in the households and older siblings.

2. Discuss: linking face washing with handwashing before meals – it is not really a separate activity.
3. Ask participants whether they have any questions or other ideas about how they can find time to prioritise helping their family achieve *Faces of Dignity*.
4. Encourage participants to try them all and find the solution which works at best for them.

ACTIVITY 6: SOAP GIVEAWAY

Purpose – Soap is consistently available and accessible for washing.

What to do

1. Turn the flipchart – SOAP GIVEAWAY page.
2. Tell participants that in addition to our tips and advice to always have soap, we are providing them with soap now because we really want them to be *Faces of Dignity* in their community.
3. **Give 1 body soap** to each family.
4. Remind them that a small part of it was used to make soapy water. Encourage them to try the soapy water and continue making it if they like it.
5. Tell participants that this soap could be put in the soap dish they got at the first Family Forum if their previous body soap is finished already or that they can store it in their home until their previous soap runs out.
6. Before moving on to the Conclusion activity, ask children to join back the forum. Congratulate children on their drawings and give one wax crayon to each child.

ACTIVITY 7: CONCLUSION

What to do

1. Turn the flipchart – CONCLUSION page.
2. Using the puppet, tell participants that we are now at the end of this family forum. Thank them for their participation.
3. Ask participants whether they have any questions or concerns.
4. Tell participants that the HV will come to visit them in their individual households in a week to see how they are doing, to answer any questions, and to check on their *Faces of Dignity*.
5. Remind participants that their Community cannot be dignified if all its members are not dignified. Their community's dignity will be publicly recognized and rewarded only if everyone in the community has a *Face of Dignity*.
6. Check that every family leaves with: **a soapy water bottle, a string and a body soap.**
7. Play the Dignity Song on a cell phone or any other device while participants are leaving the forum.
8. Put the puppet back into its cover.
9. Wash your hands with water and soap or alcohol-based sanitizer.

End of Family Forum 2.

Report Household Head Names of households who missed the forum on the Family Forum 2 Follow-up Visits Form before ending the event.
These households should be invited to join other Family Fora 2 held with other households in their cluster.
If they cannot join another session, individual follow-up with these families will be organised at their home.
Refer to Appendix A for details of the content to cover during this visit.

Appendices

Appendix A. “Follow-up with any households that do not attend the Family Forum 2”

HVs should do their best to ensure all households attend Family Forum 2 (in their original group or joining another group in their cluster). If any families do not attend, they will need to be followed up at home to go through the main activities of the forum and provide the extra giveaways (soapy water bottle, soap). If it is not possible to do this visit before the 1st House Call, the House Call should be extended so the extra content can be covered.

Setting

- Sit outside with the female primary caregiver and any family member present at home. Make sure the participants are comfortable, i.e. they should be sat in the shade.
- All materials required for the visit are organised and ready to be used.

Materials

General
- A cell phone or any other device to play the Dignity Song
- A facemask, alcohol-based sanitizer, sealable plastic bag for mask disposal

Soapy water demonstration
- 1L full water bottle
- 1 nail and 1 string
- 1 soap

Activities

Introduction

1. Wash your hands with soap or alcohol-based sanitiser and ensure you are wearing a facemask before entering the compound. Explain why you are wearing a facemask. If possible, stay outside to do the visit.
2. Greet the female primary caregiver / female adult. If they are not home **do not proceed: wait or return later.**
3. Say that the visit is short (15 mins) and ask her to gather any family members who are close by.
4. Tell the household you have come because they missed the second Family Forum and you would like to share with them what was discussed at the forum. Ask why they didn’t attend.
5. Ask if they have any questions before you start your visit.

Building and using the station

1. Lead a discussion around the following topics to explore barriers and solutions to the construction of a wash station stand:
    a. Can you show us your wash station?
    b. How do you feel about your station? Should we take it away again or is it useful?
    c. Did you have any problems building it?
    d. Are you having any problems using it?
2. Discuss solutions to any problems that are raised.
3. Lead a discussion around the following topics to explore barriers and solutions to the use of the wash station for face washing with soap 3x a day:
    To the children (if applicable), ask:
    a. Are you using the wash station? If so, have you found it easier to wash your faces with soap now that you are using the wash station?
    b. Have you felt any difference on your skin now you are washing your face with soap regularly? Discuss issues around the skin becoming dry.

To the adults, ask:

c. *If there is a child in the household:* Are you using the wash station to wash your children's faces/your face?
d. Is it easy to use the wash station to wash your children's faces / your face *(if no child)* at all three times of day (morning, before lunch and before dinner), or is it not practical to use it at a particular time?
e. Have you found it hard to make sure that there is always water in the container? What is your solution? E.g. filling it as soon as water is collected.
f. What about making sure there is always soap in the soap dish and that this is kept at the wash station? What is your solution?
g. Are you moving your wash station inside at the end of the day? Why?
h. Have you been remembering to wash your children's faces / your face (if no child) three times a day, in the morning, before lunch and in the evening before dinner? What solutions have you found to help you to do this? E.g. setting an alarm, linking to the daily routine.

4. Discuss solutions to any problems that are raised.
5. Ask the household whether they have any remaining questions or comments related to the wash station or its use.

Troubleshooting issues

1. Ask the household whether they feel they might have some issues washing their faces 3x a day with soap using the wash station in the next days, weeks or even months. Discuss.
2. Say you would like to talk in a bit more detail about some of the issues that they have raised or that other people in their community have found to be problems, especially lack of water and lack of soap.
3. Play the water perception video. *NB. Might be removed if not feasible to show the video.*
   a. Ask people what they think the key message of this video is (Answer: that having water for face washing is more a matter of priority than an issue of scarcity).
4. Soapy water demonstration:
   a. Explain that we can wash faces with soapy water if we do not have bar soap. Ask the family whether they have ever done this before.
   b. Demonstrate how to create soapy water using a plastic bottle.

Soapy water demonstration

1. Disinfect the water bottle with by rubbing it with alcohol-based sanitizer.
2. **Give** the household **a full plastic water bottle of 1L**.
3. Put a small piece of soap in the bottle. Give the rest of the bar soap to the family and inform them it can be used at the station for face washing.
4. Ask the female primary caregiver / female adult to shake the plastic bottle many times.
   → Tell her to stop here and leave it to dissolve for a day and finish the rest later.
5. Explain that after the soap has dissolved, she should shake the bottle vigorously again.
6. Check the water creates a good lather, if not, add more soap and shake again.
7. Make a hole in the lid of each plastic bottle using a nail.
8. Use the soapy water for face washing and use clean water to rinse.
9. At the end of the demonstration, the activator reports how it feels to use the soapy water **"Oh, I am definitely using soap and not just water, I can smell it, and it feels different."**

   c. Explain how the soapy water bottle can be attached using a string to the wash station. **<u>Give a string</u>** to the participant.
   d. Say that soapy water can last a lot longer than the same amount of bar soap.
   e. Ask whether they have questions.
5. Quickly discuss other solutions to troubleshoot lack of soap:
   a. Prioritising soap use for face washing instead of laundry, i.e. cutting soap
   b. If there are children in the household: Prioritising pre-school children for soap use (the ones with most discharge on their face and being the faces of their families in the community)
   c. In worst case scenario, if soap is not available at all, to keep washing faces with water three times a day and reintroduce soap as soon as possible.
6. Ask the household whether they have any questions related to troubleshooting any issues.

Face washing demonstration

1. Ask the female primary caregiver / adult female to wash both her face and her pre-school child's face (if she has children) using the wash station.
2. Before she washes, ask her the following questions:
   a. How can you make sure to remove all visible and invisible discharge?
      i. Briefly discuss wiping: should be done with the hand, hands should be washed with soap immediately to remove discharge.
   b. How many times a day should you wash our children's faces / your face with soap to ensure they have *Faces of Dignity*? When?
   c. Whose *Faces of Dignity* are the most important?
3. After she has washed herself / her child, congratulate her on being a role model for her children to copy her behaviour and for supporting her child to have a *Face of Dignity*.
4. Hand washing with soap: *(households with children only)*
   a. If the mother washed the child's hands, congratulate the mother.
   b. If the mother did not wash the child's hands, ask her to do so now and remind her that face and hand washing with soap should always go together unless someone is washing their own face with their hands.
5. Ask every other family member present (including male adult) to wash their faces using the wash station. Play the Dignity Song while every family member is washing.
6. Congratulate all family members and say that washing our family's faces 3x a day with soap, washing our eyes and nose, and making sure the younger ones are prioritised and supported every time, will help us achieve *Faces of Dignity*, faces that will make us dignified and respected people.

"A Dignified Day" Poster check

1. Ask the household to show you the poster they were given at the last forum.
   a. <u>If the poster is put on the wall:</u>
      i. Congratulate the household.
      ii. Ask all the family members to raise their hands if they intend to keep doing these activities so that they continue to lead dignified lives, be *Faces of Dignity* in their community and contribute to enhancing their community's dignity.
   b. <u>If the poster is not on the wall:</u>
      i. Ask one of the children / female adult if he/she remember the dignified activities on the poster that help a family have a dignified day.
      ii. Advise the family to put the poster up as a way to remind them about doing these activities every day to lead dignified lives and being *Faces of Dignity* for their family and in the community.

Conclusion and Giveaways

1. Say that this is the end of the visit. Ask if they have any question or concerns. Thank them for their time.
2. Inform the household that HV will come to visit them at their home in a week to see how they are doing, to answer any questions, and to check on their *Faces of Dignity*.
3. Check that the household has received: **a soapy water bottle, a string and a body soap**.
4. Wash your hands with water and soap or alcohol-based sanitizer after leaving the compound. Ensure you are safely disposing your facemask in a sealable plastic bag at the end of the morning visits or at the end of the day.

EVENT 4: HOUSE CALL 1 – CHECKLIST

Household ID: ☐☐☐☐☐☐ Date*: ☐☐/☐☐/☐☐

Start time*: ☐☐:☐☐ End time*: ☐☐:☐☐ **Use Ethiopian calendar and times*

Household Head Name: ____________________________ HV Name: ________________________________

Materials

- COVID-19 prevention: 1 facemask, alcohol-based sanitiser, sealable plastic bag to dispose of the mask
- 1 pen
- 1 dangler with 1 string and 1 nail
- 1 wash station flyer (if needed)
- A cell phone or any other device to play the Dignity Song

ACTIVITY 1: INTRODUCTION

1. Wash your hands with soap or alcohol-based sanitiser. Wear a facemask before entering the compound and explain why. If possible, stay outside to do the visit.
2. Greet the female (or male) primary caregiver. If they are not home do not proceed: wait or return later.
3. Say that the visit is short (20 mins) and is to see how they are getting on with their new wash station.
4. Ask the caregiver to gather any family members who are close by.
5. Ask if they have any questions before you begin.

ACTIVITY 2: FACIAL CLEANLINESS ASSESSMENT AND FACE WASHING

1. Ask who in the household has washed their face so far that day. Congratulate them if they all report having washed. Remind them that young children are the most important: The family cannot be truly dignified if the children are not dignified.
2. For the youngest child aged 1 – 6 years present at home, **observe and record**:

◯ Tick if 'No children aged 1 – 6 years were home' – Otherwise, record information below:

Age: ◯◯ years (enter 01 for age=1, 02 for age=2, etc.) Gender: ◯ Male ◯ Female

Face washing reported that morning: ◯ Yes ◯ No

Facial Cleanliness Assessment *(tick all that apply)*

◯ Ocular secretions present i.e. presence of clear or cloudy fluid, or dry matter on the lid margin or eyelid (including in the corners)

◯ Nasal secretions present i.e. presence of wet or dry discharge outside the nostril. Please do not stare up the nostril to find discharge but see the discharge visible outside the nares

◯ Any fly landing on face during examination

3. Ask the caregiver to ask the same child (aged 1- 6 years) to wash their face as they normally would. If a child this age is not present in the household, ask an older child or another household member to wash. Play the Dignity Song while the child is washing face.
4. During the demonstration, **record** the following:

Put an "X" in the box if the person washing: *(tick all that apply)*<br>◯ Uses the wash station ◯ Uses soap<br>If the person washing is a child aged 1-6 years, put an "X" if they: *(tick all that apply)*<br>◯ Are washed by someone else (e.g. mother, father, sibling).<br>◯ Wash/are washed thoroughly around the eyes and nose.<br>◯ Wash/have their hands washed.<br>The tap of the container was closed whilst the person washing was scrubbing his/her face:<br>◯ Yes ◯ No

5. Discuss any points on the checklist that are missed.

Activity 3: Wash station review

1. Tell the family that you would now like to have a look at their wash station.
2. Ask questions, **record** answers and discuss any issues:

Answer the following questions by marking with an "X": *(tick all that apply)*<br>◯ **Wash container** is present → If not, ask where it is: ______________________________<br>◯ Wash container is **inside** OR ◯ Wash container is **outside**<br>◯ **Wash station stand** is present → Discuss height<br>◯ Wash container has **water** (open the tap to see whether water flows out of the tap)<br>◯ Wash container is **functional,** i.e. tap is working and not leaking, container is not damaged<br>◯ **Soap** is present<br>◯ **Soap dish** is present<br>◯ **Soapy water** is present

3. If the wash station stand has not been built, ask to see their wash station flyer and go through it. Give a new flyer if they cannot locate it.
4. If the wash station is damaged say you will report it.

Activity 4: "A Dignified Day" poster review

1. Ask to see the "Dignified Day" poster and **record** the following.
2. Poster present in home:

◯ No ◯ Yes, on wall → Discuss if too high ◯ Yes, not on wall → Discuss placing somewhere visible

Activity 5: Dangler giveaway

1. Ask if they have any problem remembering to wash faces and hands before meals.
2. Say that you have a gift to help them remember to wash faces and hands before lunch and dinner.

House dangler

3. Ask where they usually have their meals and ask to see the room.
4. **Give 1 dangler** to the family as a reminder to wash faces and hands before eating lunch and dinner.
5. Fix the dangler on the wall in a place indicated by the family. Use **the nail and rope**. **Record:**

Dangler put up in household: ◯ Yes, on wall → Discuss if too high ◯ No

Activity 6: Conclusion

1. Ask if they have any questions.
2. Say that someone will return in a week to see how they are getting on with using their wash station for face washing x3 a day as a family and to certify them as a dignified family.
3. Congratulate the household on their hard work to create new habits to maintain *Faces of Dignity* for their whole family, especially the youngest children who cannot maintain their dignity on their own.

4. Congratulate the household for contributing to enhancing their Community's dignity.
5. Thank the household for their time and end House Call 1.
6. Wash your hands with water and soap or alcohol-based sanitiser after leaving the compound. Ensure you are safely disposing of your facemask in a sealable plastic bag at the end of the morning visits or at the end of the day.

Please keep this checklist safe. Give it to the responsible person from Berhan when you next see them.

EVENT 5 – HOUSE CALL 2

ACTIVATOR MANUAL

**Purpose**	To provide ongoing support to motivate families to wash faces with soap 3x a day throughout the year. Provide support to help families maintain their wash stations.
**Responsible parties**	1 trained Activator
**Participants**	All members of a household present at the time of this unannounced visit. Households living within the 'yolk' of an intervention cluster who received House Call 1.
**Location**	Each participant's home
**Duration**	30 to 45 mins
**Timing**	Workdays (except Friday morning), 9am to 5pm

Setting

- Sit in a comfortable place chosen by the household.
- Ask permission to move around their home with them during the visit.
- Seek shade or move inside when using the tablet so that everyone can see the screen clearly.

Materials

General

- COVID-19 prevention materials: 1 facemask, alcohol-based sanitizer, sealable plastic bag to dispose the mask
- House Call 2 – Checklist
- Caltu's puppet
- A pen
- A tablet with full battery – *NB. To amend if not feasible.*
- Notebook
- A cell phone or any other device to play the Dignity Song

Video-Demo

- Trachoma Transmission Routes diagram

Certification

- Certification sticker

Activities

Activity 1: Introduction

Purpose

- To provide introduction to the household visit.
- To address any concerns or questions arising from the first House Call or the Campaign.

What to do

1. Wash your hands with soap or alcohol-based sanitiser and ensure you are wearing a facemask before entering the compound. Explain why you are wearing a facemask. If possible, stay outside to do the visit.
2. Greet the female (or male) primary caregiver and thank them for welcoming you into their home.
   NB: if the female primary caregiver is not home **do not proceed with the visit until they can be found**. Either wait for them to return home, or return later that day or the following day.
3. Conduct the visit with the Household Head and any other household members if the household does not have a female primary caregiver.
4. Say that the visit will take no more than 45 mins.
5. Ask for any family members not present who are close by to be sent for.
6. Say that you are visiting to see how they are getting on with using their wash station.

Activity 2: Video demo

Purpose

- Can consistently wash young children's hands and face with soap using an effective and efficient technique.
- Self-efficacy relating to effective and efficient washing technique for self and pre-school children.
- Perceive soap as important to use each time faces are washed.
- Accept responsibility for hygiene of young children.

What to do

1. Ask a child (aged between 1 and 6yo) to wash their face as they normally would. If no young children are in the home, select an older child or another household member. Do not probe or give any recommendations.
2. Video the person washing from the time when you ask them to wash until the time they finish washing and play the Dignity Song during the demonstration.
   NB. This will be amended after pilot-testing in the field.
3. **Complete the checklist.**
4. Play the video back to the household.
5. Congratulate the volunteer / household on the parts of the wash they did "correctly" and say that it is clear they are working hard to maintain their *Faces of Dignity*
6. Discuss:
   a. **Usual practice:** Is this how the child/person usually washes? Why / why not?
   b. **Wash station use:** Who uses the wash station and when? Discuss any barriers to use and attempt to find solutions.
   c. **Soap use**: Soap should be used for all face washes, especially small children who represent the family in the community and need help to maintain their *Faces of Dignity*. Discuss any barriers raised and attempt to find solutions.
   d. **Support (if the person washing was a pre-school child):** Ask whether the family believes young children can wash their own faces thoroughly enough to remove discharge and remind them that they need help every time they wash.

e. **Thorough washing of the eyes and nose:** We need to rub around the eyes and nose every time we wash our faces. Encourage children to close eyes tightly.
f. **Closing the tap to avoid wasting water**: During every wash, the tap should be closed whilst the person washing (or being washed) is scrubbing his/her face with soap.
g. **Hand washing:** Show the Trachoma Transmission Routes diagram and remind family that our hands are naturally washed when we wash our faces, but any children we wash need their hands washed too. Both hands and faces should be clean to prevent eye and other infections. Remind caregiver that wiping should be done with the hand and hands should be washed with soap immediately to remove discharge.

7. Remind the household that everybody needs to wash faces with soap x3 a day to maintain their dignity and set a good example, but that young children are especially important and should be supported until they go to school.

ACTIVITY 3: WASH STATION REVIEW

Purpose

- Can maintain the functioning of a constructed wash station.
- Functioning wash station is consistently available and accessible.
- Self-efficacy relating to maintenance of wash station.

What to do

1. Ask to view the wash station and **complete the checklist**.
If the household **does not have a wash station,** ask what they have done with it and why. Discuss how they can make sure soap and water are always readily available for face and hand washing.
If the household **has a wash station** or **has a wash station without a wash station stand** discuss:
    a. **Water availability:** Are they able to ensure water is always available at the wash station? Discuss potential solutions e.g. filling the wash station immediately after water is collected / at a particular time of day. Dedicating someone to fill the station.
    b. **Soap availability:** Are they able to ensure soap is always kept at the wash station? Discuss potential solutions e.g. use of soapy water, asking household head for money to replace soap when it starts to run low.
    c. **Location:** Encourage the family to protect the wash station from the sun to avoid sun damage.

ACTIVITY 4: WASH STATION MAINTENANCE

Purpose

- Can repair a dysfunctional wash station.
- Self-efficacy relating to repair of wash station when it becomes dysfunctional.
- Functioning wash station is consistently available and accessible.

What to do

1. Play the Wash Station Maintenance video on the tablet**.**
2. Repeat the video if the household requests it, or if the household is large and cannot all view the screen. Aim to get the attention of the Household Head as a priority.
3. Discuss the video and answer any questions about replacing broken or leaky taps.

NB. This activity will be piloted and amended according to feasibility. If this can't be set up, key elements regarding wash station maintenance should be communicated to Activators during the training, so they can facilitate a discussion with the households instead of showing a video.

Key elements include: habits and behaviours to adopt to avoid damage to the wash station container (esp. making sure the tap does not break by not moving the station around, go collect water with it, etc.), what to do if the tap leaks (e.g. use glu around the tap, where to get it, how much it costs), what to do if the tap breaks (e.g. where to get a new tap, price, how to fix the tap, etc.)

ACTIVITY 5: POSTER AND DANGLER REVIEW

Purpose

- Perceive face washing to be important for maintaining dignity of self and family.
- Perceive effectively washing face at least three times per day as important, all year around.
- Accept responsibility for hygiene of young children.

What to do

1. Ask participants to see their "Dignified Day" Poster and **complete the checklist.**
2. Ask participants to see their Dangler and **complete the checklist.**
3. If poster and dangler are present, congratulate the HH on having their poster and dangler. Remind that these are cues to remind them about face washing 3 times a day and that, if they have phones, setting an alarm 3 times a day can also help them.

ACTIVITY 6: WASH STATION CERTIFICATION

Purpose

- Engaging, accessible, appropriate, strategically placed washing prompts/cues are visible.
- Perceive effectively washing face at least 3x a day as important, all year around
- Perceive face washing to be important for maintaining dignity of self and family.
- Perceive soap as important to use each time faces are washed
- Perceive an expectation from neighbours & husbands to maintain clean faces of self & family
- Create the impression that everyone in the community washes faces with soap x3 a day

What to do

1. Take Caltu's puppet in your hand and animate the activity with the puppet (only if some children are present).
2. Congratulate the family again on working hard to maintain their *Faces of Dignity* and on enhancing their Community's dignity.
3. Show the video of the Influential Role Model congratulating the family on their *Faces of Dignity*, thanking them for being dignified community members and explaining that the a certification sticker acknowledges their efforts at washing faces with soap x3 a day and should be placed on the wash station. **Refer to Appendix A** for content.
   NB. This is likely to be an audio-recorded message from the Influential Role Model. To be amended after piloting.
4. If the family has a wash station and a wash station stand:
   a. **Give** the Household Head (or nearest representative) **the Certification sticker** and ask them to place it on the wash station.
5. If the family has a wash station but has still not built a wash station stand:
   a. Provide personalised suggestions to troubleshoot the issues with building a stand and remind participants of the benefits of using one (e.g. easiness of use, etc.)
   b. **Give** the Household Head (or nearest representative) **the Certification sticker** and ask them to place it on the wash station.
6. If the family does not have a wash station any longer:
   a. Provide advice on dedicating other materials (e.g. a specific jug or bowl) for face and hands washing.

b. **<u>Give</u>** the Household Head (or nearest representative) **<u>the Certification sticker</u>** and ask them to place it on the chosen dedicated material.

7. **Complete the attached checklist.**

ACTIVITY 7: CONCLUSION

What to do

1. Lead this activity using the puppet (only if some children are present).
2. Say that we are now at the end of this HH visit.
3. Ask if there are any final questions about any aspect of this visit or the *Faces of Dignity* campaign.
4. Say that they will be visited again [tell them the likely month/season of this visit].
5. Congratulate the household on their hard work to create new habits to maintain *Faces of Dignity* for their whole family, especially the youngest children. Encourage them to keep washing faces x3 a day so they represent themselves well in the community.
6. Say that a little gathering will take place at *[location where the Dignity Banner is up in the community]* after all households of their community have been visited. If everyone has become a *Face of Dignity*, their community will be publicly recognized as a Dignified Community and rewarded in presence of all the community leaders. HVs will inform them about the gathering and they are welcome to join the celebration.
7. Thank the family for their time.
8. Put the puppet back into its cover.
9. Wash your hands with water and soap or alcohol-based sanitize after leaving the compound. Ensure you are safely disposing your facemask in a sealable plastic bag at the end of the morning visits or at the end of the day.

End of House Call 2.

NB. At the end of House Call 2 delivery, community leaders will be gathered at the location where the Dignity Banner is up to publicly declare the community a Dignified Community. The Dignity Banner will be amended at the occasion. Consecutively, community leaders will be rewarded for their contribution to the intervention. This section will be amended after discussing options with the field team and Berhan.

Appendices

Appendix A. "Content of Influential Role Model Testimonials – Wash station certification"

Testimonial 4: **"Congratulating participants"** | Interviewee: Lead Influential Role Model

"Congratulations for working so hard to become a dignified family and all having *Faces of Dignity*. I personally want to thank you all for being dignified community members. Your community is proud of you as everyone needs to work together to ensure our community all have *Faces of Dignity*. Thanks to all of you, our community is a Dignified Community! The certification sticker which is given to you now acknowledges your efforts over the last month to wash your faces x3 with soap a day to become *Faces of Dignity*. Like me, stick it on your wash station container to always remember your progress. Congratulations again and keep up the good work!"

EVENT 5: HOUSE CALL 2 – DATA FORM

Household ID: ☐☐☐☐☐☐ Date: ☐☐/☐☐/☐☐ Time: ☐☐:☐☐

Ethiopian calendar *Ethiopian time*

Household Head Name: ______________________________ Activator Name: ___________________________

VIDEO DEMO

Age: ◯◯ years (enter 01 for age=1, 02 for age=2, etc.) Gender: ◯ Male ◯ Female

Put an "X" in the box if the person: *(tick all that apply)*

- ◯ Uses the wash station with tap
- ◯ Uses soap. If yes, which soap was used: ◯ Laundry soap ◯ Body soap ◯ Soapy water
- ◯ Is supported by someone (e.g. mother, father, sibling)
- ◯ Washes clearly around the eyes and nose
- ◯ Closes the tap whilst scrubbing his/her face
- ◯ Has hands washed (if face wash is performed by someone other than the child)

WASH STATION REVIEW

Water container with tap is present:

◯ Yes, inside ◯ Yes, outside ◯ Cannot be seen → Ask where the water container is

Record where it is: ______________________________

Family has built a station for their wash station water container:

- ◯ Yes, suitable height.
- ◯ Yes, too high → Discuss changing the height so it can be used by more family members.
- ◯ No → Discuss how they are using the material and how the station could help them.

There is water in the wash station container: ◯ No ◯ Yes, but no water flows when the tap is open ◯ Yes, 1/4 full ◯ Yes, half full or more

The wash station container is functional: ◯ Yes ◯ No, why: ______________________________________

There is bar soap at the station: ◯ Yes, in soap dish ◯ Yes, not in soap dish ◯ No

There is a soapy water bottle at the station: ◯ Yes, attached to the station ◯ Yes, not attached ◯ No

POSTER AND DANGLER REVIEW

Location of the poster: ◯ On wall, suitable height

◯ On wall, too high → Suggest changing location and explain why

◯ Present, but not visible → Suggest nailing to wall

◯ Not seen in home ◯ Other: ________________________________

Location of the dangler: ◯ Hung. If yes, where: ◯ In the kitchen ◯ Place people take meals

◯ Present in the house, but not visible → Suggest hanging the dangler

◯ Not seen in home ◯ Other: ________________________________

WASH STATION CERTIFICATION

Certification sticker has been given to household: ◯ Yes ◯ No
Certification sticker has been stuck on the wash station container or a dedicated material: ◯ Yes ◯ No

Please keep this checklist safe and give it to the responsible person at Berhan when you see them.

REINFORCEMENT EVENTS
ACTIVATOR MANUAL

**Purpose**	Reinforce key messages and narratives of the Campaign (face washing with soap x3 a day, with an emphasis on preschool children) and overcome seasonal barriers to face washing behaviour.
**Responsible parties**	2x trained Activators and 1 Health Volunteer
**Participants**	All adults living within the 'yolk' of an intervention cluster – 10 household groupings
**Location**	TBD – A host household or a public space
**Duration**	45 mins to 1h (maximum)
**Timing**	Workdays (except Friday morning), 9am to 1pm and after 3pm
**Times of year**	Dry season: End of December/Early January for February (driest month in the year)<br>Rainy season: June for July/August (rainiest two months of the year)<br>Final: anytime

Preparation

Week before the start of the delivery

- Train Activators on the content of the Reinforcement Event.

Day before the event

- Provide all materials to the Activators or Health Volunteers.
- Mobilise Activators or Health Volunteers to invite 10x neighbouring households to the event based on the groupings of the Family Forums.
- Ask Activators or Health Volunteers to identify a suitable location for the event, ideally in the compound of one of the households or a public space.
- Communicate time and location of the event to each participating household.
- In the morning, ask Activators or Health Volunteers collect data on the status of wash stations within the cluster.
- *If applicable:* In the afternoon, ask Health Volunteers (HVs) to invite eight individuals, including themselves among kebele leaders, Health Extension Workers (HEWs), community leaders/elders, the kebele women and youth affairs leader, and religious leaders/influential role models. They will provide feedback on the status of their wash stations, engage in a brief discussion based on this feedback, and establish a direction for future improvements.
- *If applicable:* ask Activators to select role model HHs who will provide live testimonials on their experience of using wash stations to practise face washing with soap three times a day. They should also share strategies for overcoming barriers associated with this practice during the Reinforcement Event.

Setting

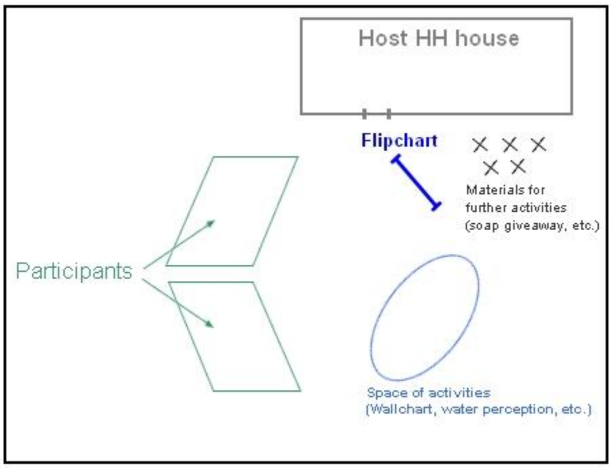

Materials

For all reinforcement events

General
- Flipchart (for use of specific pages + 5 Problem Solving storyboards) *(except for the Second Rainy Season Reinforcement)*
- 2 or 3 tablets to show the "Wash station maintenance" video (First Rainy Season Reinforcement), play the Dignity Song, and record data regarding face station maintenance (Second Rainy Season Reinforcement)
- Face wipes (approximately 1/child) + Green clay for face
- 2 jugs + 2 water collectors + 20L jerrycan full of water

Soap giveaways
- 10 body soaps (1 per household)

First rainy season reinforcement

Maintaining *Faces of Dignity*
- 7 Velcro flashcards

Water perception
- 1 full plastic water bottle of 1L
- 1 full jerrycan (water) of 5L
- 2 full plastic water bottles of 2 litres
- 2 full plastic water bottles of 1litres
- 2 full plastic water bottles of ½ litres

Soapy water
- 1 full plastic water bottle of 1L
- 1 body soap (to make soapy water demonstration + to be cut)
- 1 nail

Dry season reinforcement

Maintaining *Faces of Dignity*
- 7 Velcro flashcards

Wash station maintenance
- Socket set (including bi-hex and full hex) for wash station maintenance

Second rainy season reinforcement

Wash station maintenance
- Socket set (including bi-hex and full hex) for wash station maintenance

Soapy water
- 1 full plastic water bottle of 1L
- 1 body soap (to make soapy water demonstration + to be cut)
- 1 nail

Final reinforcement

Campaign Reminder
- Laminated image of the *Faces of Dignity* Family (A3 or A2)
- *A Dignified Day* Poster

Role as a Role Model Short drama
- Two series of 10 laminated "Role as Role Models" flashcards (10 for men and 10 for women)
- Laminated colour images with the father's role, mother's role, and parent's role
- 2 jugs + 2 water collectors
- Clothing for Activators (funny attire)
- 20 blue and red cards

Soapy water
- 10 full plastic water bottles of 1L
- 10 body soap (to make soapy water demonstration + to be cut)
- 10 nails
- 10 small ropes
- Line drawing of Caltu's puppet (1 per child) + Wax crayons(1/child)

Set up

Day of the reinforcement event

- Do you have all materials required for all activities?
- Is equipment working?
  Tablets have full battery and the drama film is on each tablet.

EVENT 6 – REINFORCEMENT EVENT: RAINY SEASON

ACTIVATOR MANUAL

ACTIVITY 1: INTRODUCTION & SIGNPOSTING TO *THE FACES OF DIGNITY*

What to do

1. Set up the flipchart – REINFORCEMENT EVENT: RAINY SEASON COVER page.
2. Play the Dignity Song on a tablet or cell phone while participants are arriving.
3. Greet the group and welcome them to the event.
4. Advise community members to sit with their household members and respect a physical distance of at least 2m with other households.
5. Ask for a show of hands to see who remembers the *Faces of Dignity* campaign.
6. Invite one or two people to share what they remember from the campaign (be sure to correct them if anything they say is incorrect)
7. Say that you are here to continue your discussions about face washing with soap.
8. Say that the event will not be long and they will receive a gift at the end.

Round table

1. Raise your hand if you feel you have a *Face of Dignity*. Ask volunteers to comment on why they feel this
2. Raise your hand if you feel your children have *Faces of Dignity* that represent your family. Ask volunteers to comment on why they feel this
3. Raise your hand if you feel your community have a *Face of Dignity*. Ask volunteers to comment on why they feel this

4. Confirm the key messages of the *Faces of Dignity* campaign through Q & A with the group:
    a. **Why do we need to wash our faces?** Face washing with soap helps maintain beauty and dignity and thus helps gain respect in the community and enhance the community' dignity.
    b. **How many times a day should faces be washed?** Faces should be washed with soap x3 a day: after waking in the morning, before lunch and before the evening meal.
    c. **When should soap be used during face washing?** Always!
    d. **Which family members should wash their faces with soap x3 a day?** All of them! Young children should be assisted to wash so that eyes and noses can be thoroughly cleaned of any dirt and trachoma-carrying discharge. Soap is essential to remove discharge. Face washing should be a familial activity, where pre-school children are prioritised but everybody is washing to set a good example for the younger ones
    e. **Why do we need to use soap when we wash our faces?** To remove the discharge that can contain trachoma and stop it from spreading to the eyes of other members of our family.

A Dignified Day

1. Turn the flipchart – A DIGNIFIED DAY page.
2. Remind participants that earlier in the Campaign we agreed on a list of daily activities which help us gain *Faces of Dignity* and maintain our family's *Face of Dignity*. These activities were listed on the posters they were given.
    a. Ask the group:
        i. Do you remember the dignified activities on the poster that help a family have a dignified day?

    ii. If they cannot remember all the activities, show them any outstanding flashcards, ask the group what the image represents, and put the flashcard on the flipchart.

  b. Ask the whole group to pledge to keep doing these activities so that their families can continue to lead dignified lives, achieve *Faces of Dignity* and keep enhancing their Community's dignity

Trachoma Transmission Routes

1. Turn the flipchart – TRACHOMA TRANSMISSION ROUTES page.
2. Show the group the TRACHOMA TRANSMISSION DIAGRAM and discuss routes of transmission.

ACTIVITY 2: FACE WIPE EMO-DEMO – SHORT VERSION

What to do

1. Ask participants/children if they see any difference between the face of the two Activators.
2. Ask if the faces look clean.
3. Explain to participants that you are going to use wipes to confirm what they have just said. Explain that the wipe is just a cloth that is a bit wet.
4. Wash your hands with soap before starting.
5. Ask participants which wipe they expect to be dirty.
6. The activators should wipe the RIGHT side of their faces and show the group the wipes
7. Ask the audience what they see: both wipes are dirty. Is this a surprise?
8. Say that faces can look clean even when they are not. Say that we need to use soap to get them really clean and dignified.
9. Ask the audience if they agree with this

ACTIVITY 3: PROBLEM IDENTIFICATION & TESTIMONIALS

What to do

1. Ask the group what problems they expect to face continuing to wash their faces with soap x 3 a day in this season.
2. Ask if they have any challenges using the wash station. Listen and facilitate the discussion for a few minutes.
3. Tell the group we are going to discuss some of the main challenges they have raised and think about how they can be overcome.
4. Turn the flipchart – PROBLEM SOLVING STORYBOARD page.

Live Testimonials

1. Invite the role models HHs to comment on the problems raised by the community during discussion.
2. Ask them to share their experiences of how they have overcome the obstacles/challenges raised by the community and how they are washing their hands and face with soap three times a day using this washing station.
3. Ask the audience if they agree with this and tell them that there is something we can do together to prove what they have just mentioned. *NB: invite role model HHs to respond on from the same cluster, (if possible) who provide statement testifying their experiences of using wash station' on reinforcement event one day before event.*
4. Invite Role Model HHs to in front of gathered people to share their experience of using wash station to practice face washing with soap 3x a day and testifying of how to overcome barriers associated with this practice to become dignified family. How important their behaviour is and how it also reflects well on the community.

5. Tell the participants that you are going to discuss some of the main obstacles they have raised
6. Use the storyboard between each activity below to help structure the discussion about problems and solutions.

Activity 4: Problem-Solving – Lack of Time

What to do

1. Explain to participants that many of their neighbours who complained about lack of time for face washing in the past, are now reporting that the face washing station has considerably changed the time it takes for face washing. It is really quick now.
2. Say that you will prove it with a race:
   a. Ask for two volunteers. One needs to wash their face with soap using a HW station, the other needs to wash their face with soap in the way they do when they don't use a HW station.
   b. Everyone can see that the person who used the HW station finished washing much faster than the other person
3. End the activity by reminding people that if they put water in the wash station and soap nearby they can easily wash their faces all day long.
4. Discuss that they do a lot of activities every day. The activities they choose to do are ones that are important. They can fit face washing in if they choose to prioritise it. Isn't it important to give our children the gift of a *Face of Dignity*?

Other solutions

1. Sharing the responsibility of face washing with other adults in the households and older siblings.
2. Linking face washing with handwashing before meals – it is not really a separate activity.
3. Ask participants whether they have any questions or other ideas about how they can find time to prioritise helping their family achieve *Faces of Dignity*.
4. Encourage participants to try them all and find the solution which works at best for them.

Activity 5: Problem-Solving – Forgetfulness

What to do

1. <u>Turn the flipchart</u> – Lack Of Time and Attention page.

Forgetfulness

1. Explain that we often remember to do something when we see something in our home or environment that is connected to the activity we want to do.
2. Give suggestions: put the wash stations somewhere you will see it when you go to eat so you remember to wash face and hands, put an alarm on your phone if you have one, help remind each other, put the dignified day poster somewhere you will see it regularly,
3. Ask participants whether they have any ideas of other things that could help them remember to wash their faces and their children's faces with soap 3x a day. When there is no water, advise participants to keep washing with water 3 times a day anyway.
4. Tell them that they should not break their habit and feel discouraged. They should rather keep washing with water and reintroduce soap as soon as possible.
5. Ask participants whether they have any remaining questions or ideas related to soap and keeping soap at the station.
6. Encourage participants to try them all and find the solution which works best for them. Say that in life we always find a way to do something when it is important to us, and the *Faces of Dignity* of our family is really important so we know that everyone will work hard to make sure they always have soap.

ACTIVITY 6: PROBLEM SOLVING – WASH STATION USE AND MAINTENANCE

What to do

1. Ask for a show of hands of any families with problems making sure there is water in their wash station.
2. Explain that they don't need to fill it up completely in order to use it as that would take too much water. Turn the flipchart and show them picture of wash station container
3. Discuss who in the family is responsible for checking the water and putting it in the wash station and who will help them to remember to do it.
4. Ask for a show of hands if anyone has any problems with their wash station, is the tap working OK still?
5. Play the Wash Station Maintenance video on the tablet. *NB. This will be amended according to feasibility.*
6. Repeat the video if the group requests it.
7. Discuss the video and answer any questions about replacing broken or leaky taps.

ACTIVITY 7: PROBLEM SOLVING – LACK OF WATER

What to do

1. Turn the flipchart – LACK OF WATER page.

Water perception demonstration

1. Tell the participants that we have spent time observing how people in the community use water for different activities in the household.
2. Take out your cups/bottles with different quantities of water in them.
3. Say that these quantities of water correspond to daily activities. Show them the laminated pictures of these activities (cooking, doing dishes, coffee making/washing coffee cups and face washing with soap).
4. Get the group to guess which amount of water corresponds to which activity. They should place the picture cards in front of the cup/bottle they think it relates to.
5. When the group has finished, tell them their order starting from minimum amount of water they think a typical family uses to perform that activity daily. For instance, if you need to so it reads like this:
    a. Face washing with soap (work out what quantity is minimum you can use)
    b. Coffee making and other coffee-related activities – 3 litres
    c. Cooking – 4 litres
    d. Doing dishes – 4 litres
6. Draw their attention to the smallest amount of water with the picture of face washing with soap. Ask one volunteer HHs members (average 5 persons) to wash their faces with the water in the bottle (1liter) with soap to prove it can be done with very little water. Tell them that 5 family members can use (amount of water they used to wash their faces with soap multiply by three) amount of water to wash their faces with soap three times a day.
7. Ask participants to raise their hands if they agree that other daily activities consume more water than a whole family face washing.
8. Reinforce message that even when water is scarce they have enough for face washing 3x a day as it does not consume much water.
9. Tell participants that water is sometimes scarce and we should teach our children to use it carefully and to turn off the tap on the wash station when water is not needed.

ACTIVITY 8: PROBLEM-SOLVING – LACK OF SOAP

What to do

1. Turn the flipchart – LACK OF SOAP page.

Soapy water demonstration

1. Remind the group that you gave them bottles and they made soapy water.
2. Discuss experiences making and using soapy water.
   a. Who used the soapy water they made?
   b. Who made more when the soapy water ran out?
3. Say that you will do a quick demonstration to remind the group how to make soapy water, for those who find it useful.

Soapy water demonstration

11. Take a full plastic water bottle of 1L.
12. Put a small piece of soap in the bottle.
13. Shake the plastic bottle many times.
    → Tell participants that, when they are preparing soapy water, they should stop here and leave it to dissolve for a day and finish the rest later.

14. HV demonstrates how to finish making the soapy water after they have left the soap to dissolve.
15. Shake the bottle vigorously again.
16. Check the water creates a good lather, if not, add more soap and shake again.
17. Make a hole in the lid of each plastic bottle using a nail.
18. Use the soapy water for face washing and use clean water to rinse.
19. At the end of the demonstration, the HV reports how it feels to use the soapy water **"Oh, I am definitely using soap and not just water, I can smell it, and it feels different."**

ACTIVITY 9: SOAP GIVEAWAY

What to do

1. **Give** each household **with 1 body soap**. Encourage them to put it in their soap dish and use it only for body washing.

ACTIVITY 10: CONCLUSION

What to do

1. Turn the flipchart – CONCLUSION page.
2. Tell participants that we are now at the end of this Reinforcement Event.
3. Thank them for their time and participation.
4. Ask whether they have any remaining questions or concerns.
5. Remind the group that we will host other events like this in the coming months.
6. Play the Dignity Song while participants are leaving the event.
7. Wash your hands with water and soap or alcohol-based sanitizer.

End of Rainy Season Reinforcement.

EVENT 7 – REINFORCEMENT EVENT: DRY SEASON
ACTIVATOR MANUAL

ACTIVITY 1: INTRODUCTION & SIGNPOSTING TO *THE FACES OF DIGNITY*

What to do

1. Set up the flipchart – REINFORCEMENT EVENT: DRY SEASON COVER page.
2. Play the Dignity Song on a tablet or cell phone while participants are arriving.
3. Greet the group and welcome them to the event.
4. Advise community members to sit with their household members and respect a physical distance of at least 2m with other households.
5. Ask for a show of hands to see who remembers the *Faces of Dignity* campaign.
6. Invite one or two people to share what they remember from the campaign (be sure to correct them if anything they say is incorrect)
7. Say that you are here to continue your discussions about face washing with soap.
8. Say that the event will not be long and they will receive a gift at the end.

Round table

1. Raise your hand if you feel you have a *Face of Dignity*. Ask volunteers to comment on why they feel this
2. Raise your hand if you feel your children have *Faces of Dignity* that represent your family. Ask volunteers to comment on why they feel this
3. Raise your hand if you feel your community have *Face of Dignity*. Ask volunteers to comment on why they feel this

4. Confirm the key messages of the *Faces of Dignity* campaign through Q & A with the group:
   a. **Why do we need to wash our faces?** Face washing with soap helps maintain beauty and dignity and thus helps gain respect in the community and enhance the community' dignity.
   b. **How many times a day should faces be washed?** Faces should be washed with soap x3 a day: after waking in the morning, before lunch and before the evening meal.
   c. **When should soap be used during face washing?** Always!
   d. **Which family members should wash their faces with soap x3 a day?** All of them! Young children should be assisted to wash so that eyes and noses can be thoroughly cleaned of any dirt and trachoma-carrying discharge. Soap is essential to remove discharge. Face washing should be a familial activity, where pre-school children are prioritised but everybody is washing to set a good example for the younger ones
   e. **Why do we need to use soap when we wash our faces?** To remove the discharge that can contain trachoma and stop it spreading to the eyes of other members of our family.

A Dignified Day

1. Turn the flipchart – A DIGNIFIED DAY page.
2. Remind participants that earlier in the Campaign we agreed on a list of daily activities which help us gain *Faces of Dignity* and maintain our family's *Face of Dignity*. These activities were listed on the posters they were given.
   a. Ask the group:

   i. Do you remember the dignified activities on the poster that help a family have a dignified day?
   ii. If they cannot remember all the activities, show them any outstanding flashcards, ask the group what the image represents, and put the flashcard on the flipchart.

b. Ask the whole group to pledge to keep doing these activities so that their families can continue to lead dignified lives, achieve *Faces of Dignity* and keep enhancing their Community's dignity

Trachoma Transmission Routes

1. Turn the flipchart – TRACHOMA TRANSMISSION ROUTES page.
2. Show the group the TRACHOMA TRANSMISSION DIAGRAM and discuss routes of transmission.

ACTIVITY 2: DEMONSTRATION OF THE BENEFITS OF WASHING WITH SOAP

What to do

1. HVs and activator both paint of their faces with clay. When applying, they should apply so that participants cannot see them. They both stand in front of the participants and ask what their faces look like.
2. Ask participants/children if they see any difference between the face of the two Activators. Participants will be asked to raise their hands and tell them what they understand
3. They both start washing their faces saying that you will follow us to see what we are going to do.
4. The activator washes his face and hands thoroughly with soap and water. When washing, he should wash his face back to the participants.
5. HVs washes his face and hands with water only. When he washes, he should wash slightly so that the dirt remains on his face. He should wash his face back to the participants
6. After washing, they both stand in front of the participants and ask them to tell them the difference between the two faces. If the participants raise their hands and say, "The face washed with soap and water is clean and there is no dirt on it," the activator should say and has to confirm that "yes, my face is clean. I feel very happy." If the participants raise their hands and say "there is visible dirt on the face washed with water only." HVs should say and has to confirm that "yes, it is true that washing the face with water alone does not clean the dirt and discharge properly as you can see now. This can easily expose us to trachoma."
7. Ask the audience if they agree with this
   Conclusion: The activator will conclude the following points:
   a. As we have been teaching you on face emo demo in the past during the family forum, our face can be dignified and respectful and dirt can only be removed properly if we wash it with soap.
   b. Please confirm that you agree with this idea by raising your hand. Conclude by confirming that participants agree

ACTIVITY 3: PROBLEM IDENTIFICATION AND TESTIMONIALS

What to do

1. Ask the group what problems they expect to face continuing to wash their faces with soap x 3 a day in this season.
2. Ask if they have any challenges using the wash station. Listen and facilitate the discussion for a few minutes.
3. Tell the group we are going to discuss some of the main challenges they have raised and think about how they can be overcome.

4. Turn the flipchart – PROBLEM SOLVING STORYBOARD page.
5. Live testimonial: Invite the role models HHs to comment on the problems raised by the community during discussion.
6. Ask them to share their experiences of how they have overcome the obstacles/challenges raised by the community and how they are washing their hands and face with soap three times a day using this washing station.
7. Ask the audience if they agree with this and tell them that there is something we can do together to prove what they have just mentioned. *NB: invite role model HHs to respond on from the same cluster, (if possible) who provide statement testifying their experiences of using wash station' on reinforcement event one day before event.*
8. Invite Role Model HHs to in front of gathered people to share their experience of using wash station to practice face washing with soap 3x a day and testifying of how to overcome barriers associated with this practice to become dignified family. How important their behaviour is and how it also reflects well on the community.
9. Tell the participants that you are going to discuss some of the main obstacles they have raised
10. Use the storyboard between each activity below to help structure the discussion about problems and solutions.

ACTIVITY 4: PROBLEM-SOLVING WASH STATION USE AND MAINTENANCE & DISCUSSION ON PERCEPTIONS OF ROLES (MEN AND WOMEN)

What to do

Wash station maintenance

1. Ask for a show of hands if anyone has any issues with their wash station. Is the tap still working properly?
2. Instead of showing a wash station maintenance video, each activator should be provided with a socket set (including bi-hex and full hex) to demonstrate wash station maintenance during every group discussion with 10 households. To carry out this activity, the activator should follow these steps:
   a. He/she should have a wash station container with a drain/leak.
   b. Stand in front of the participants and show the participants how to repair/maintain the wash station container. After the maintenance/repair, he/she will show the participants that the container does not leak. If their wash station is leaked water, he/she will remind participants that they should repair/maintain it accordingly
   c. Repeat the how to repair/maintain the wash station container if the group requests it.
   d. Discuss the repair steps and answer any questions about replacing broken or leaky taps.
   e. Inform participants that socket sets will be available in their clusters through a volunteer, and if they encounter similar issues, they can ask the volunteer for assistance with repairs.

Perceptions of roles (men and women)

1. The activator will conduct an open discussion in the form of Q and A with the participants on the following points: The activator will let the participants to share their family experiences by raising their hands
   a. **Wash station container taking out and in**: Who in your household member monitors the wash station to take out in the morning and take in in the evening?
   b. **Replacing soap:** How are you replacing soap when it runs out? Who in your family is given responsibility?

c. **If the wash station runs out of water**: Who in your household members is refilling the wash station container regularly before it runs out?
d. **If the mother travels**, who in your family will make /help the young children (children who cannot wash on their own) to wash their face and hands?
e. The activator will conclude based on the comments given from participants

ACTIVITY 5: PROBLEM-SOLVING – LACK OF WATER

What to do

1. Turn the flipchart – LACK OF WATER page.

Water perception demonstration

1. Tell the participants that we have spent time observing how people in the community save water using their wash station.
2. This activity should be done in a competitive manner between Activator and HVs.
3. The activator should wash his face and hands with soapy water using wash station.
4. The HVs should wash his hands and face using a water collector and jug
5. The activator should save water when washing face and hands. When washing, he/she should use soap watery hanging near the wash station container. Activator should use water and less time while washing
6. The HVs should wash with excess water when washing. He also has to find and bring a water collector, jug, water and soap before washing. HVs should use more water and time while washing
7. To determine the amount of water they use to wash their faces and hands. When they both wash, they must wash carefully so that the waste water does not fall off the ground and has to be collector on water collector
8. Both activator and HVs wastewater should be placed in a half-litre bottle and shown to participants. Ask participants to voluntarily raise their hands and tell the difference between the waste water in the two bottles. Participants should appreciate the differences they see.
9. Ask participants if they agree that using wash station container saves water and that many families can wash with less water. One person washing face and hands using water collector consumes more water and time than a whole family face washing. Make sure they agree.
10. Remind them that using a wash station is a solution to their water shortages and lack of time so that they can save water and time.
11. Reinforce message that even when water is scarce they have enough for face washing 3x a day as it does not consume much water.
12. Tell participants that water is sometimes scarce and we should teach our children to use it carefully and to turn off the tap on the wash station when water is not needed.

ACTIVITY 6: PROBLEM-SOLVING – LACK OF SOAP

What to do

1. Turn the flipchart – LACK OF SOAP page.

Live testimonials: Soapy water

1. Remind the group that you gave them bottles and they made soapy water.
2. Discuss experiences making and using soapy water.
   a. Who used the soapy water they made?
   b. Who made more when the soapy water ran out?
3. Invite volunteer HHs from participant who is currently using wash station properly to show the following points to participants:

4. He/she prepares his/her own soapy water and will show the participants. He/she tells participants that soapy water is very easy to prepare
5. He/she tells to participants that more than twenty one-litre bottles of soapy water can be prepared with a bar of soap
6. He/she used to prepare soapy water from a bar of soap and tell the participants that a bar of soap will serve them for more than two months.

Conclusion

1. Ask "Do you agree with what they just told us?" to ensure that the participants agree. Ask participants to confirm it by raising their hands in agreement.
2. As people have just told us, it is very easy to prepare soapy water.
3. We have learned from model HHs that many soapy waters can be prepared from a bar of soap and that a bar of soap can be used for at least two months. I would like to ask other participants to apply the same procedure as we have just seen.

Activity 7: Problem-Solving – Procrastination: Short Drama

What to do

1. Turn the flipchart – PROCRASTINATION page.

Procrastination

1. Play the short Procrastination Drama

The Activator, acting as a volunteer, goes to follow up with a household that has not set up their wash station outside.

Aba Chala:
Good morning/afternoon, Hadha Caltu!
Hadha Caltu:
Good morning/afternoon, Aba Chala. What brings you by? Are you well? Come in, please.
Aba Chala:
Haven't you put the wash station outside today, like you did the last couple of days?
Hadha Caltu:
Thank you, I'm well. Do come in. Apologies – I got caught up with something just now. I was about to finish it and then take the wash station outside.
Aba Chala:
Hadha Caltu, let's sit and have a word about this. I'm here for the third day now, and I still haven't seen any change.
Hadha Caltu:
I know, I just have this small task in hand, then I'll get right to it.
Aba Chala:
Alright then, I'll wait while you finish.

After a few minutes…
Aba Chala:
Are you ready now?
Hadha Caltu:
Yes, what is it you wanted to discuss?

Aba Chala:

Hadha Caltu, I've come by for three days now. What's stopping you from setting up the wash station outside?

Hadha Caltu:

You're right, you've come each day, and each time, I've said I'd finish what I'm doing and put it out.

Aba Chala:

Please, tell me honestly – what's really keeping you from setting it up? Are you and your family actually washing your hands and faces with soap and water?

Hadha Caltu:

Lying is forbidden, and yes, we try to keep up with it as best we can.

Aba Chala:

But if the wash station isn't even out, how are you all washing properly? What's the real issue?

Hadha Caltu:

You're right. They taught us how to use it, showed us how to use soap – we understand it well enough. But like the old saying goes, "A habit doesn't leave a person, just like a hill doesn't move."

Aba Chala:

So you know the benefits, but what's really getting in your way, Hadha Caltu?

Hadha Caltu:

It's this procrastination – every day I say I'll do it, and then I push it off. I even meant to put it out today before heading to the hospital.

Aba Chala:

Is everything alright? What's taken you to the hospital?

Hadha Caltu:

I haven't slept all night – my eyes sting, and it feels like they're burning. I haven't had a wink of sleep.

Aba Chala:

Please, go and wash them with soap.

Hadha Caltu:

Yes, I'll get up and do just that.

After washing...

Hadha Caltu:

Goodness! It's like a miracle! I feel so much better – if only I'd washed like this three times a day, I wouldn't be in this state.

Aba Chala:

That's exactly the point. From now on, keep the wash station outside and wash with soap and water three times daily, for yourself and your family.

Hadha Caltu:

You're right. From today, I promise we'll wash three times a day – once in the morning when we wake, before lunch, and again before dinner.

Aba Chala:

That's good, Hadha haltu. Let's make it a habit – washing with soap and water three times a day will help prevent trachoma. Take care!

Hadha Caltu:

Thank you, Aba Chala, for looking out for us. Have a good day.

ACTIVITY 8: SOAP GIVEAWAY

What to do

1. **Give** each household **with 1 body soap**. Encourage them to put it in their soap dish and use it only for body washing.

ACTIVITY 9: CONCLUSION

What to do

1. Tell participants that we are now at the end of this Reinforcement Event.
2. Thank them for their time and participation.
3. Ask whether they have any remaining questions or concerns.
4. Remind the group that we will host other events like this in the coming months.
5. Play the Dignity Song while participants are leaving the event.
6. Wash your hands with water and soap or alcohol-based sanitizer.

End of Dry Season Reinforcement.

EVENT 8 – REINFORCEMENT EVENT: RAINY SEASON
ACTIVATOR MANUAL

Purpose Focus on getting the faces of pre-school children washed with soap and providing resources for wash station maintenance. Continue to address lack of soap and water and encourage role modelling. Reinforce key intervention's messages.

Implementers Health Volunteer x1

Participants All individual households in intervention 'yolk'. Individual household visits followed by small gatherings of 5 to 10 neighbouring households.

Duration 15 minutes in individual households followed by 30 minutes in small groups.

Location Appropriate host house or public space that is not too noisy / distracting.

Timing Workdays (except Friday morning), 9am to 1pm and after 2pm. On the first day in each cluster, Activators will replace damaged wash stations and assist in constructing wash station stands using locally available materials.

INDIVIDUAL HOUSEHOLD'S EVENT (15 MINS)

ACTIVITY 1: WASH STATION CHECKS

What to do

1. Open the data record form "Wash station check" on the tablet.
2. Observe the wash station in each individual home and complete the form:

1. **Observe:** is the wash station present in the home?
NB. This question is about the physical presence of the wash station, not whether it is functional / in use.
◯ Yes, inside ◯ Yes, outside ◯ No
Record where it is: ____________________
2. **Observe:** if yes, does the wash station have water?
Turn the tap and check if water comes out.
◯ Yes, but water is below the level of the tap
◯ Yes, water flows when the tap is turned
◯ No
3. **Observe:** is the wash station functional?
The wash station could be used to wash hands and faces if it had water in it e.g. there is a stand of some kind, the tap is working and not leaking, the container is not damaged etc. This question is about functionality, not use.
◯ Yes *(go to question 5)*
◯ No *(go to question 4 and skip questions 5, 6 and 9)*
4. **If no, ask:** why is the wash station not functional? *Select all that apply.*
◯ No stand ◯ Tap is broken ◯ Tap is leaking ◯ Container is damaged
◯ Other: ________________
5. **Observe:** is soap present? ◯ Yes ◯ No *(go to question 7)*
6. **If yes, record:** which kind of soap is present?
◯ Soapy water ◯ Other kinds of soap

7. **Observe:** is a soap dish present? ◯ Yes ◯ No
8. **Ask:** is there soap available elsewhere in the home?

Ask the caregiver to bring the soap for you to see it.

◯ Yes (seen) ◯ Yes (not seen) ◯ No

9. **Observe:** is there water on the ground or in a water collector below the wash station?

Does the wash station appear to be in use.

◯ Yes *(go to question 11)* ◯ Unsure *(go to question 10)* ◯ No

10. **Ask:** for which purposes are you using this wash station? *Select all that apply*

◯ Body washing ◯ Other purposes ◯ Not being used

11. **Ask:** Which of the following challenges do you face using the wash station?

Read out each challenge and select all that apply.

◯ Lack of soap
◯ Lack of water in the home
◯ Filling the wash station with water (when there is enough water)
◯ Absence of wash station stand
◯ Fear of theft of wash station
◯ Do not find it easy to use
◯ Prefer to wash in another way
◯ Other
◯ None of the above

3. Thank the household for responding to your questions.

Group Event (30 mins)

ACTIVITY 1: Wash station Maintenance

What to do

1. Instead of showing a wash station maintenance video, demonstrate wash station maintenance using the provided socket set (including bi-hex and full hex). Follow/demonstrate the following steps:
   a. Stand in front of the participants and show the participants how to repair/maintain the wash station container. After the maintenance/repair, show the participants that the container does not leak. If their wash station is leaked water, remind participants that they should repair/maintain it accordingly
   b. Repeat the how to repair/maintain the wash station container if the group requests it.
   c. Discuss the repair steps and answer any questions about replacing broken or leaky taps.
   d. Inform participants that socket sets will be available in their clusters, and if they encounter similar issues, they can ask you for assistance with repairs.

ACTIVITY 2: Demonstration of the Benefits of Soap

What to do

1. Paint your face with clay without being seen by the household members – rub it in so the face looks clean.
2. Gather the family and tell them you are going to explain why soap is important.
3. Ask them if your face looks clean (they should agree that it does).

4. Wash the left side of your face with water only, and the right side with water and soap. Explain what you are doing as you do it. *(Use your own soap and water).* Remind participant how little water you are using and catch the waste water in a bowl.
5. Wipe the left side of your face with a face wipe and show the family that it is dirty. Remind them that you washed this side of your face with just water. Wipe the right side of your face with another face wipe and show the family that it is clean. Remind them that you washed this side of your face with water and soap.
6. Ask participants to explain what they have understood: you need to use soap to get a face truly clean. Tell them you are an adult and your face is this dirty. Say that it is even more important to wash our children's faces and to use soap as children get more dirty than adults and washing faces is an easy way to maintain dignity.
7. Tell the family that you know that they do not always have soap, and when they do not have soap they should still wash their face and their children's faces as using water only is better than not washing at all.

Activity 3: Problem-Solving – Lack of Soap

What to do

1. Ask participants if they remember that they can make soapy water if they find it hard to always have enough soap in the home.
2. Take your bottle with water and cut up bits of bar soap in it. Show them the small bits of soap and the water and remind them how to make the soapy water.

Soapy water demonstration

1. Take a full plastic water bottle of 1L.
2. Put a small piece of soap in the bottle.
3. Shake the plastic bottle many times.
   → Tell participants that, when they are preparing soapy water, they should stop here and leave it to dissolve for a day and finish the rest later.
4. HV demonstrates how to finish making the soapy water after they have left the soap to dissolve.
5. Shake the bottle vigorously again.
6. Check the water creates a good lather, if not, add more soap and shake again.
7. Make a hole in the lid of each plastic bottle using a nail.

Activity 4: Wash-Along

What to do

1. Ensure all pre-school children and their caregivers join and participate in this activity.
2. Before the children are washed by their caregivers, make sure that there is soap and water in the same place. If they do not have any soap, give them a small soap.
3. Invite all participant dyads (caregivers and children) to wash their faces with soap.
4. When the faces have been washed, continue:
   a. Ask the gathered participants if the faces look clean, i.e. there is no discharge around the eyes or nose. If needed, ask them to wash the face again.
   b. Ask the gathered participants to recognise that the mother can wash the child's face better than the child can do it themself. Say that young children often wash their own faces, but they do not rub well around the eyes and nose. It is better to do it for them so we know their face is truly clean and dignified.
   c. Comment on how much easier it is to wash a child's face when the soap and water are kept together (at the wash station).

d. Remind the caregivers that they can save water by using the wash station, but they need to turn the tap off.
e. Say that children's faces should always be washed when they wake up and before they go to bed. We should do this even if there is no soap.

ACTIVITY 5: CONCLUSION

What to do

1. Tell participants that we are now at the end of this Reinforcement Event.
2. Thank them for their time and participation.
3. Ask whether they have any remaining questions or concerns.
4. Remind the group that we will host other events like this in the coming months.
5. Wash your hands with water and soap or alcohol-based sanitizer.

End of Second Rainy Season Reinforcement.

Event 9 – Final Reinforcement Event

Activator Manual

Purpose Focus on getting the faces of pre-school children washed with soap, with family role modelling to ensure all family members also wash faces with soap. Continue to address lack of soap and water and encourage role modelling.

Implementers Activators x2 (1 to lead the men's event and 1 to lead the women's event before the groups merge into a Whole Family Event) with support of x2 Health Volunteers.

Participants All intervention families living in 'yolk' households. Kids should attend the women's group so it is easy to make sure they are present when the groups are merged for the joint session.

Duration 30 minutes for separate groups, followed by up to 1 hour in the combined group.

Location Appropriate host house or public space that is not too noisy / distracting.

Timing Workdays (except Friday morning), 9am to 1pm and after 2pm. On the first day in each cluster, Activators will replace damaged wash stations and assist in constructing wash station stands using locally available materials.

Men's Event (30 mins)

Activity 1: Introduction & Campaign Reminder

What to do

1. Greet the group and welcome them to the event.
2. Ask participants: what do you remember from our previous sessions? *Community Event, Family Forums, House Calls and two Reinforcement Events.*
3. Ask for a show of hands:
    a. Who remembers the *Faces of Dignity* campaign?
4. Put a large A3/A2 image of the *Faces of Dignity* family in the middle of the group.
5. Ask volunteers to share what they remember about the *Faces of Dignity* campaign. Be sure to correct them if they say anything wrong.
6. Recap the key messages through Q & A with the group:
    a. **Whose face should be washed?** Faces of all family members should be washed.
    b. **Whose faces wash needs to be assisted and prioritized?** Young children should be assisted to wash so that eyes and noses can be thoroughly cleaned of any dirt and trachoma-carrying discharge. Soap is essential to remove discharge. Face washing should be a familial activity, where pre-school children are prioritised but everybody is washing to set a good example for the younger ones.
    c. **What times of day should faces be washed?** Faces should be washed with soap x3 a day: after waking in the morning, before lunch and before the evening meal.
    d. **When should soap be used?** Always! But if you don't have any still wash faces!
    e. **Why do we need to use soap when we wash our faces?** To remove the discharge that can contain trachoma and stop it from spreading to the eyes of other members of our family.
5. Talk about dignity, ask the group:
    a. What types of activities help you maintain dignity?
        i. Show the group the Dignified Day Poster or Flipchart page (large images) to help the discussion.

b. Raise your hand if you feel you have a *Face of Dignity*. Ask volunteers to comment on why they feel this.
c. Raise your hand if you feel your children have *Faces of Dignity* that represent your family in the community. Ask volunteers to comment on why they feel this.

Activity 2: Role as a Role Model

What to do

1. Ask the group what a role model is. Who are your role models from your family and why?
2. Ask the group if they believe they are important role models for their children.
3. Show the group a series of cards depicting activities to do with face washing and wash station maintenance. The cards to be piloted are as follows:
   a. Role modelling face washing by washing own face with soap at same time as children.
   b. Making sure there is budget to have soap in the home at all times.
   c. Notice when soap / soapy water is running low and inform the person who will replace it so there is always soap at the wash station.
   d. Purchasing soap when the soap is running low (before it runs out).
   e. Making soapy water when the soap is running low (before it runs out).
   f. Making sure the wash station is kept in the location where it is used (e.g. outside) every day, not just when someone is coming to check it.
   g. Making sure the wash station stand is properly made and durable.
   h. Making sure the wash station is well maintained and used properly so it isn't damaged.
   i. Making sure there is water in the wash station and replacing it when it runs out.
   j. Supporting each other as a family to wash faces with soap together in the morning and evening (times when they are all together).
   k. Washing children's faces for them.
4. Ask them to group the cards into three groups to show whose role they are: the father's role; the mother's role; or both parents' role.
   a. Stick each card into the column they select on the flipchart using velcro.
   b. When they have finished sorting the cards into groups, discuss any cards that you feel are in the wrong group and try to persuade the group to regroup them.

Activity 3: Problem-Solving – Wash Station Maintenance

What to do

1. Wash station stands
   a. Show the group an A3 or larger laminated image of a good, durable wash station stand that has been made with locally available materials (no cost).
   b. Using the laminated image discuss how to make a sustainable stand like this. Discuss drainage. Discuss where the stand will be located.
2. Maintenance of taps (leaking / breaking).
   a. Who has problems with their wash station, is the tap working OK still?
   b. Remind the group how to avoid breaking the taps. This should have just been discussed in Activity 2.
   c. Go through the following maintenance points using a wash station container with a drain/leak:
      i. Stand in front of the participants and show the participants how to repair/maintain the wash station container. After the maintenance/repair, he/she will show the participants that the container does not leak. If their wash station is leaking water, he/she will remind participants that they should repair/maintain it accordingly

   ii. Repeat the how to repair/maintain the wash station container if the group requests it.
   iii. Discuss the how to repair/maintain and answer any questions about replacing broken or leaky taps.

Activity 4: Problem-Solving – Lack of Soap

What to do

1. Ask volunteers to share why it is important to use soap or soapy water. Be sure to correct them if they say anything wrong.
2. Remind the group that in our previous discussion about the preparation of soapy water we confirmed that a bar of soap can be used for more than two months.
3. Ask for a show of hands: Who provides the budget for purchasing soap and is ultimately responsible for soap being available in the household?
4. Look at the tasks related to soap on the flipchart. Go back through the cards that the group agreed were men's responsibilities related to soap.
   a. Remind your wife, elder son, or daughter to regularly prepare soapy water and ensure that it is always available next to the wash station
   b. Remind everyone in the family to wash their faces with soap using a wash station
   c. Give money to the wife if it runs out.
   d. Supporting children to wash faces with soap, and role modelling this for them.

Women's Event (30 mins)

Activity 1: Introduction & Campaign Reminder

What to do

1. Greet the group and welcome them to the event.
2. Ask participants: what do you remember from our previous sessions? *Community Event, Family Forums, House Calls and two Reinforcement Events.*
3. Ask for a show of hands:
   a. Who remembers the *Faces of Dignity* campaign?
4. Put a large A3/A2 image of the *Faces of Dignity* family in the middle of the group.
5. Ask volunteers to share what they remember about the *Faces of Dignity* campaign. Be sure to correct them if they say anything wrong.
6. Recap the key messages through Q & A with the group:
   a. **Whose face should be washed?** Faces of all family members should be washed.
   b. **Whose faces wash needs to be assisted and prioritized?** Young children should be assisted to wash so that eyes and noses can be thoroughly cleaned of any dirt and trachoma-carrying discharge. Soap is essential to remove discharge. Face washing should be a familial activity, where pre-school children are prioritised but everybody is washing to set a good example for the younger ones.
   c. **What times of day should faces be washed?** Faces should be washed with soap x3 a day: after waking in the morning, before lunch and before the evening meal.
   d. **When should soap be used?** Always! But if you don't have any still wash faces!
   e. **Why do we need to use soap when we wash our faces?** To remove the discharge that can contain trachoma and stop it spreading to the eyes of other members of our family.
7. Talk about dignity, ask the group:
   a. What types of activities help you maintain dignity
      i. Show the group the Dignified Day Poster or Flipchart page (large images) to help the discussion.

b. Raise your hand if you feel you have a *Face of Dignity*. Ask volunteers to comment on why they feel this.
c. Raise your hand if you feel your children have *Faces of Dignity* that represent your family in the community. Ask volunteers to comment on why they feel this.

Activity 2: Role as a Role Model

What to do

1. Ask the group what a role model is. Who are your role models from your family and why?
2. Ask the group if they believe they are important role models for their children.
3. Show the group a series of cards depicting activities to do with face washing and wash station maintenance. The cards to be piloted are as follows:
    a. Role modelling face washing by washing own face with soap at same time as children.
    b. Making sure there is budget to have soap in the home at all times.
    c. Notice when soap / soapy water is running low and inform the person who will replace it so there is always soap at the wash station.
    d. Purchasing soap when the soap is running low (before it runs out).
    e. Making soapy water when the soap is running low (before it runs out).
    f. Making sure the wash station is kept in the location where it is used (e.g. outside) every day, not just when someone is coming to check it.
    g. Making sure the wash station stand is properly made and durable.
    h. Making sure the wash station is well maintained and used properly so it isn't damaged.
    i. Making sure there is water in the wash station and replacing it when it runs out.
    j. Supporting each other as a family to wash faces with soap together in the morning and evening (times when they are all together).
    k. Washing children's faces for them.
4. Ask them to group the cards into three groups to show whose role they are: the father's role; the mother's role; or both parents' role.
    a. Stick each card into the column they select on the flipchart using velcro.
    b. When they have finished sorting the cards into groups, discuss any cards that you feel are in the wrong group and try to persuade the group to regroup them.

Activity 3: Soap!

What to do

1. Ask volunteers to share why it is important to use soap or soapy water. Be sure to correct them if they say anything wrong.
2. Remind the group that in our previous discussion about the preparation of soapy water we confirmed that a bar of soap can be used for more than two months
3. Ask for a show of hands: Who takes on the responsibility of regularly buying soap for their family when it runs out?
4. Look at the tasks related to soap on the flipchart. Go back through the cards that the group agreed were men's responsibilities related to soap.
5. Go back through the cards that the group agreed were women's responsibilities. Show the women how many tasks relate to soap.
    a. Regularly prepare soapy water and ensure that it is always available next to the wash station
    b. Remind everyone in the family to wash their faces with soap using a wash station
    c. Prioritise washing faces of young children with soap
    d. Ask husband for money for soap if it runs out.

Whole Family Event

NB. Ensure that children (especially preschool children) are present (hopefully they will have already been with the women in the previous session so it is quick to merge the two groups into family units.

Activity 1: Roles & Responsibilities

What to do

1. Tell the group that the sessions they just participated in had very similar content, looking at roles and being a role model.
2. Bring out the flipcharts from the men's and women's groups and show the group what was discussed. The purpose of this is to get everyone to take accountability for their responsibilities in front of their husband/wife/community.
3. Discuss each card.
    a. Ask those people for suggestions of how each task can be achieved.
4. Conclude the activity :
    a. Ask the group if they notice how many of the tasks are their role / the role of both parents. Tell them they need to support their wives so the whole family has *Faces of Dignity*.
    b. Tell the group that even if they are succeeding, no one is perfect and they can always improve.
5. Get the group to look at the cards and decide which 1 thing they will absolutely take away from this session and implement at home. This should be done individually, they may not all focus on the same thing. Go around the group and get each person to say out loud what they will do and how they will make sure they achieve it *[this is like a pledge to each other – saying it out loud gives them some accountability].*
6. Show the *Faces of Dignity* family image, printed large and laminated. Ask the group whose face should be washed if there is only enough water and soap to wash one face. If they don't say the youngest child, remind them that young children are the most likely to get dirty faces and to have trachoma and their faces should be washed to stop trachoma spreading to other people.

Public pledge

1. Say that the family must work together to ensure everyone washes their faces with soap, adults should wash their own faces to model the behaviour for children.
2. Ask each family to hold hands to form a circle.
3. Ask them to repeat the pledge out loud: ***"A clean face is attractive, it is also dignifying. I promise to do my part to make sure there is always soap and water available for face washing in our household. I will wash my face with soap as an example for our children. We will support each other to wash our children's faces."***
4. Ask each family to discuss how they will support each other.

Conclusion

1. Conclude the activity by congratulating the families for working together to maintain their family's dignity.

ACTIVITY 2: SUCCESS TESTIMONIALS

What to do

1. Acknowledge that there are barriers that they all experience related to lack of soap and water and remembering to wash faces. Ask for volunteers to share how they are <u>overcoming these barriers and succeeding</u> to wash their children's faces with soap.
2. Ask a household (HH) currently utilizing the wash station to provide a testimonial about their successful experience.
   NB. If none of the HHs present are using the wash station, showcase photos on the flipchart depicting other HHs from the same or different clusters using the wash station.
3. Remind the gathered HHs to utilize the wash station in a similar manner as other community members.

ACTIVITY 3: GET THESE CHILDREN'S FACES WASHED (WITH SOAP)!

What to do

Drama

1. Do the **short drama**. *Activators are wearing funny attire and introduce themselves, e.g. one is the father, one the mother.* Select two children aged 4 and 8 from the gathered children. They are a *"family"*. Ask the family to wash their faces in front of the group:
   a. Give *"the family"*a bowl, a jug with water and small bar of soap. Show the group how much water there is.
   b. Instruct the family to wash their own faces, and not to help their children.
   c. Use the following script for this family, but make it fun and engaging:

Aba Caltu:
Oh, what a bright morning! A new day's here, everyone!
Hadha Caltu:
Yee! (meaning "yes").
Aba Caltu:
Hadha Caltu, can you bring me some water so I can wash my face?
Hadha Caltu:
Ishii, (meaning "okay").
Aba Caltu:
I'm in a rush to get to meditation, Hadha Caltu. Make sure the whole family follows suit and washes their faces with soap.
Hadha Caltu:
Caltu, my daughter, have you seen the jug? Where's the water? Where's the soap? I put the bowl right here yesterday – why did someone move it? Every day it's the same search! Who used the soap last?

Hadha Caltu searches around the house for each of the items.

Aba Caltu:
Hadha Chaltu, why are you always so busy? Please, I'm telling you, I'm in a hurry!
Hadha Caltu:
Apologies, Aba Caltu. Here you go. I couldn't find the things where I left them – someone must have moved it all.
Aba Caltu:
What can we do? We always waste time looking for everything.

Aba Caltu quickly washes his face with soap and heads out for meditation, giving a quick, casual goodbye to the family without much affection.

Hadha Caltu washes her face, then places the soap on top of the wash station and tells the 4- and 8-year-old children to wash their faces.
The children struggle a bit. The 4-year-old tries to reach the soap, but after a few attempts, gives up and washes only with water, spilling some from the jug. The 8-year-old manages to grab the soap, applies it to her face, then reaches for the jug. Seeing it nearly empty, she shouts at the 4-year-old for spilling the water. The two exchange some cross words.

2. Now ask the volunteer family to wash their faces again, with a few changes:
    a. Give the family a wash station and soap in a soap dish placed on a wash station stand so it is easily accessible.
    b. Instruct the family to wash their own faces and then the faces of any preschool age children.
    c. Use the following script for this family.

Aba Caltu: *Muttering to himself.*
Right, up bright and early to get the wash station outside. Soapy water's ready, hanging from the wash station, and the soap's in the dish.
Aba Caltu washes his face with soap at the wash station, then calls out.

Aba Caltu:
Hadha Caltu, come on out and wash your face with soap!
Hadha Caltu: *Hadha Caltu steps outside, washes her face with soap, then calls to her children.*
Come on, children, time to wash faces!

She carefully helps her 4-year-old, washing their face with soap, making sure they're clean. She then invites her 8-year-old to wash on their own, which they manage with no trouble. The family all chat happily together, sharing a warm hug before Aba Chaltu heads off for the day.

Voting exercise

1. Give each person in the group two laminated voting cards. One blue (on both sides) with a smiley face (= easy) and one red (on both sides) with a sad face (= difficult).
2. Ask them to answer the following questions about the face washing they have just observed by voting with their cards by holding up the side of the card. Discuss the reasons why people voted as they did after each question:
    a. Was it easy (blue) or hard (red) for the family to wash their faces together?
    b. Did young children manage to wash their faces thoroughly (blue) or could they have washed them better (red)?
    c. Was it quick (blue) or did it take a long time (red) for the family to wash?
    d. Was it easy (blue) or hard (red) for everyone to find and use the soap?
    e. Did it use a little water (blue) or a lot of water (red)?
3. Ask the group to comment on what they have observed. Prompt them if needed to discuss the following points:
    a. When young children wash on their own they use a lot of water
    b. When young children wash on their own they find it hard to use soap properly
    c. When soap and water are not kept together it takes longer to wash and is more awkward
    d. When the wash station is used it makes it easier to wash young children's faces properly

Conclusion

1. Conclude that helping young kids to wash uses less water, keeps the soap clean, and gets faces cleaner so they are dignified and make the family dignified for each other and the community

Activity 4: Recap

What to do

1. Ask the group to shout out the one change each individual is going to make to support their family to wash their faces with soap x3 a day.

Activity 5: Soap Giveaway & Soapy water bottles

What to do

1. Instruct all families to prepare soapy water and take it with them. If it runs out instruct them to replenish it accordingly.
2. The children can colour in Caltu instead.

Activity 6: Conclusion

What to do

1. Tell participants that we are now at the end of this Reinforcement Event.
2. Thank them for their time and participation.
3. Ask whether they have any remaining questions or concerns.
4. Thank them for taking part of the *Faces of Dignity* Campaign
5. Play the Dignity Song while participants are leaving the event.
6. Wash your hands with water and soap or alcohol-based sanitizer.

End of Final Reinforcement.
