# Supplementary material for: Translating Formative Research into Intervention Content: Experiences with Face Washing for Trachoma Control in Rural Ethiopia
Source: Behav Sci (Basel). 2025 Mar 13;15(3):355. doi: 10.3390/bs15030355 (PMC11939790; doi:10.3390/bs15030355)
Supplement: Supplementary file 1 [file behavsci-15-00355-s001.zip › PDF files/04_Family Forum 2 Manual_Paper.pdf]

EVENT 3 – FAMILY FORUM 2

ACTIVATOR MANUAL

**Purpose**	Continue to build skills and motivation to practice face washing. Overcome early barriers related to wash station construction and use. Emphasize the need to wash faces with soap three times a day now that everyone has a functioning wash station.
**Responsible Parties**	1 trained Activator + 1 trained Health Volunteer (2 HVs will assist the Activator in each cluster to spread the work load)
**Participants**	All household members living within the 'yolk' of an intervention cluster who attended Family Forum 1 – Split into groups of 5 households.
**Location**	A HH compound (selected in advance when HHs are informed the time and date for the forum)
**Duration**	1h30
**Timing**	Workdays (except Friday morning), 9am to 1pm and after 3pm

Preparation

Day before the event

- Mobilise HVs to invite the same 5 neighbouring households as for Family Forum 1 (including households who joined other groups because they missed their session).
- HV to ask the host of the 1st forum if they will also host Family Forum 2. Ensure the selected household has constructed a wash station stand or provide encouragement so they do so before the event.
- Communicate time and location of the event to each participant household.
- Inform each household that the whole family should participate if possible.

Setting

- Set up the forum in a similar way to Family Forum 1, ensuring at least a 2-meter physical distance between members from different households. Tweak the layout as necessary to enhance the experience of the group based on your learnings from running the previous forum.

Materials

General

- COVID-19 preventive materials: cleaning solution in a spray (x1), alcohol-based sanitiser, facemasks (for Activator and HV), sealable plastic bag to dispose masks
- Flipchart
- Caltu's puppet in its cover
- Pen
- Notebook
- Cell phone or device to play the Dignity Song

Wash-Along

- Wash station containers with tap (one borrowed from the HV, one from the host household) + 2 collectors + 3 soap dishes + 3 soaps + 1 stool borrowed from the host household
- One 20L jerrycan of water (revise quantity based on experience running this event)

**A "Dignified Day"**	– 7 Velcro flashcards
**Barriers and Solutions**	– 2 jugs (+ 2 collectors + 1 soap + 1 soap dish + jerrycan of water from the Wash-Along activity)<br>– 1 coffee tray + 1 jebena + 6 coffee cups<br>– Velcro extra people to stick on the Dry season infographics<br>– 6 empty plastic water bottles 1L<br>– 5 strings for participants to attach their soapy water bottle on their wash station<br>– 3 nails<br>– 1 body soap (to make soapy water + to be cut)
**Soaps giveaway**	– 5 body soaps

Set up

Day of the family forum

- Do you have all materials required for all activities?
- Is equipment working?

Activities

Activity 1: Introduction

Purpose

- To provide introduction to the forum.
- To address any concerns or questions arising from the first Family Forum.

What to do

1. Install the flipchart – Family Forum 2 page. Install Caltu's puppet next to the flipchart on its cover.
2. Play the Dignity Song on a cell phone or any other device while participants are arriving.
3. Advice community members to sit with their household members and respect a physical distance of at least 2m with other households.
4. Greet the participants and welcome them to the forum.
5. Complete attendance sheet.
6. Ask the participants whether they have any question concerning the first family forum or the programme.
7. Tell the group that the event should not take more than 1h30. Giveaways for each household will be given at the end, so they should stay until the end of the forum.

Activity 2: Live testimonials

Purpose

- The primary purpose of this activity is to identify and overcome barriers to face washing with soap x3 a day associated with wash station construction and use.
- Can construct a functional wash station.
- Can maintain the functioning of a constructed wash station.
- Functioning wash station is consistently available and accessible.
- Perceive the wash station to be useful.
- Self-efficacy relating to construction of functional wash station.
- Self-efficacy relating to maintenance of wash station.
- Can troubleshoot complaints of young children during washing.

What to do

1. Turn the flipchart – Live Testimonials page.
2. Introduce the activity.

Building the station

1. Lead a discussion around the following topics to explore barriers and solutions to the construction of a wash station stand:
2. To everyone ask:
    a. Have you managed to build a wash station stand? → Participants raise their hands if they have built a stand. Count the number of hands.
    b. How do you all feel about your wash station? Should we take it away again or is it useful?
3. To the family hosting the forum ask:
    a. Can you show us all your wash station?
    b. Are you pleased with it? Why?
    c. Did you have any problems building it?
    d. Have you had any problems using it?
4. To the other households ask:

    a. Have you built your wash station stands differently to this one? Ask people to volunteer what they have done.
    b. Have you had any issues building your stands?
5. Discuss solutions to any problems that are raised.

Using the station

1. Lead a discussion around the following topics to explore barriers and solutions to the use of the wash station for face washing with soap 3x a day:
2. To the children, ask:
    a. Are you using the wash station? If so, have you found it easier to wash your faces with soap now that you are using the wash station?
    b. Have you felt any difference on your skin now you are washing your face with soap regularly?
        i. If children or adults mention skin being dry:
            1. Tell them that this is nothing to be worried about and that is likely to be temporary.
            2. Advise them to use a body soap (which is softer on the skin) as much as possible.
            3. If having dry skin becomes an issue, suggest applying a moisturizing cream or lotion on their children's faces with clean hands (i.e. primary caregiver should wash hands with soap before applying the cream). That cannot impede their *Faces of Dignity*.
        ii. If no one mentions skin being dry:
            1. Inform children and adults that this could happen and provide similar advice (points 1, 2, 3 above).
3. To the adults, ask:
    a. Are you using the wash station to wash your children's faces?
    b. Is it easy to use the wash station to wash your children's faces at all three times of day (morning, before lunch and before dinner), or is it not practical to use it at a particular time?
    c. Has anyone found it hard to make sure that there is always water in the container? Has anyone found any solutions? E.g. filling it as soon as water is collected.
    d. What about making sure there is always soap in the soap dish and that this is kept at the wash station? Who has managed to do this?
    e. Who is moving their wash station inside at the end of the day? What is everyone else doing and why?
    f. Have you been remembering to wash your children's faces three times a day, in the morning, before lunch and in the evening before dinner? What solutions have you found to help you to do this? E.g. setting an alarm, linking to the daily routine.
        i. Ask participants whether any of them owning a phone was able to set up an alarm 3 times a day to remind themselves and their neighbours about face washing.
            1. If yes, ask these participants to share their experience and to describe how useful the alarm is.
            2. If no, ask whether they have a phone and would like your guidance to set up an alarm 3 times a day.
4. Discuss solutions to any problems that are raised.
5. Ask participants whether they have any remaining questions or comments related to the wash station or its use.

Introduce the Wash Along

> **"Thank you all for sharing your experiences building and using your wash stations. We hope you have been able to get some answers to your questions and to learn from the experience of your neighbours to improve your own practice. We would now like to wash our faces all together using a wash station".**

ACTIVITY 3: WASH ALONG

Purpose

- Can consistently wash own hands and face with soap using an effective and efficient technique.
- Can consistently wash young children's hands and face with soap using an effective and efficient technique.
- Perceive soap as important to use each time faces are washed.
- Accept responsibility for hygiene of young children.
- Self-efficacy relating to effective and efficient washing technique for self and pre-school children.
- Perceive an expectation from neighbours to maintain clean faces and dignity of self and family.

What to do

1. Turn the flipchart – WASH ALONG page.
2. Set up materials for the Wash Along:
    a. Put the HV's wash station on a plastic chair borrowed from the host household, and ask the host household to borrow their wash station. Put 1 collector underneath each station. Bring forward the 3 soaps in soap dishes.
    b. Fill the 2 wash stations with 5L of water.
    c. Disinfect the tap of each wash station by spraying cleaning solution.
    d. Take Caltu's puppet in your hand and animate the rest of the activity with the puppet.

Face washing demonstration

1. Ask a volunteer mother to wash both her face and her pre-school child's face using a wash station.
2. Before she washes, Caltu's puppet asks the rest of the group the following questions:
    a. How can the mother make sure she removes all visible and invisible discharge?
        i. Briefly discuss wiping: remind participants that wiping should be done with hands and hands should be washed with soap immediately after to remove discharge from the hands.
    b. How many times a day should we wash our children's faces with soap to ensure they have *Faces of Dignity*? When?
    c. Whose *Faces of Dignity* are the most important? (i.e. pre-school children as they are the ones having discharge and the one going everywhere in the community and representing the family at all times)
3. Ask the group to observe as she washes.
4. After she has washed the child, clap and congratulate the mother on being a role model for her children to copy her behaviour and for supporting her child to have a *Face of Dignity*. Say that neighbours will know what she is doing and she is representing her family well in the community.
5. Put Caltu's puppet back on its cover.

Closing the tap to avoid wasting water

<u>If the mother closed the tap whilst scrubbing her child's face</u>, congratulate the mother and remind the group how important this is to avoid wasting water.
<u>If the mother did not close the tap whilst scrubbing her child's face,</u> remind the group how important this is to avoid wasting water, remind them to do so for every wash and to teach their children to do so too when there are old enough to wash themselves. Say that washing as a family means they can control how much water (and soap) the children use for face washing.

Hand washing with soap

<u>If the mother washed the child's hands</u>, congratulate the mother and remind the group how important this is.
<u>If the mother did not wash the child's hands</u>, ask her to do so now and remind the group that face and hand washing with soap should always go together unless someone is washing their own face with their hands. Hands should always be washed with soap after wiping discharge.

1. <u>Invite each household to come</u> one by one <u>at the HV's wash station and wash children's face</u> (e.g. washed or supervised by parents or elder siblings). If possible and according to time, every other household member (including caregiver and man/household head) should wash their face at the station. Disinfect the tap of the station between each household by spraying cleaning solution. Ask <u>only the host's family</u> to use their wash station.
2. Remind participants if pre-school children should be prioritized for face washing, everybody should do it to set a good example and to help children do it.
3. Remind adults to support younger children by washing their faces for them.
4. Play the Dignity Song on a cell phone or any other device while all participants are washing faces.
5. Congratulate the group when everyone has finished washing (everyone should clap).
6. Say that washing our family's faces 3x a day with soap, washing our eyes and nose thoroughly, and making sure the younger ones are prioritised and supported every time, will help us achieve *Faces of Dignity*, faces that will make us dignified and respected people.
7. Say that our neighbours notice when we have *Faces of Dignity* and they know we are contributing to enhance the community's dignity, which depends on each individual
8. Conclude the activity by encouraging the group to continue using their wash stations to act with dignity by making sure soap and water are always available for body washing.

ACTIVITY 4: "A DIGNIFIED DAY"

Purpose

- Perceive face washing to be important for maintaining dignity of self and family.
- Perceive effectively washing face at least three times per day as important, all year around.
- Accept responsibility for hygiene of young children.
- Perceive an expectation from husbands and neighbours to maintain clean faces of self and family.

What to do

1. <u>Turn the flipchart</u> – A DIGNIFIED DAY page.
2. <u>Take Caltu's puppet in your hand and animate the activity with the puppet.</u>
3. Remind participants that they were given a poster in the last forum.
   a. Ask where they have all put their posters.
4. <u>Ask the children</u>:

a. Do you remember the dignified activities on the poster that help a family have a dignified day?
    i. Each time a child gives a right answer, put the corresponding flashcards up on the Velcro flipchart using the puppet.
    ii. It they cannot remember all the activities, show them any outstanding flashcards, ask the group (children first, adults if help is required) what the image represents, and put the flashcard on the flipchart.

5. Ask the whole group to raise their hands if they intend to keep doing these activities so that their families can continue to lead dignified lives and contribute to enhancing the dignity of their community.
6. Conclude the activity by reminding the group that these are small things we can do to help our family's gain and maintain *Faces of Dignity* and contribute to enhancing the community's dignity.
7. Put Caltu's puppet back on its cover.

ACTIVITY 5: BARRIERS TO FACE WASHING & SOLUTIONS

Purpose

- Can troubleshoot reduced access to water in dry season and continue to prioritise water for hand and face washing with soap.
- Willing to prioritise water for face washing, all year around.
- Self efficacy relating to prioritisation of water for face washing, all year around.

What to do

1. Turn the flipchart – BARRIERS TO FACE WASHING AND SOLUTIONS page.
2. Before starting the Barriers and Solutions activity, propose to children (aged 2 to 7 or 8) to colour in a drawing of the puppet explaining about face washing. Tell the children they will be able to keep their drawing to remind them about when they should wash their faces to become *Faces of Dignity*. Put the puppet in a visible place. Give one drawing to each child and a few wax crayons disinfected with the cleaning solution.
3. Ask participants whether they feel they might have some issues washing their faces 3x a day with soap using the wash station in the next days, weeks or even months. Discuss.
4. Tell the group that we would like to talk in a bit more detail about some of the issues that they have raised or that other people living near them have found to be problems.

Lack of Water

1. Turn the flipchart – LACK OF WATER page.
2. The first issue is lack of water, especially during the dry season, and how this affects people's ability to keep a continuous supply of water in their wash stations.

Water perception demonstration

1. Ask participants whether they stop drinking coffee during the dry season.
2. Ask how many times a day they can prepare and drink coffee (at maximum).
3. Agree with participants that they drink coffee all year round and that each time they prepare coffee they use water to clean the materials (i.e. tray, cups, jabena) and they use water to make coffee.
4. Say that you would like to do a quick demonstration to show them something interesting.
5. Do the water perception demonstration.

Water perception demonstration

1. Both activators fill a jug with water and take a water collector.
2. Show the full jugs to participants.
3. The Activator washes his/her face with soap over a collector (trying to minimise water used).
4. The HV washes a coffee tray, 6 coffee cups and a jebena over a collector (washing thoroughly without giving the impression of overusing water).
5. Show the group the difference in the amount of water in both collectors and what is left in both jugs.

6. Ask participants to raise their hands if they agree that preparing and drinking coffee consumes more water than face washing.
7. Get them to agree that they drink coffee several times a day and therefore they also have enough water for face washing 3x a day.
8. Tell participants that water is obviously scarcer during the dry season and that we should teach our children to use it carefully and to turn off the tap on the wash station when water is not needed.

Dry season infographic

1. Turn the flipchart – FACE WASHING IN THE DRY SEASON page.
2. Tell participants that you realise some of them may not yet be convinced that they will have enough water for their whole family to wash their faces with soap x 3 a day, all year round.
3. Say that we watched people in a nearby community using water in their homes in the dry season, rainy season and at harvest time. We want to show them what we found.
4. Discuss the Dry Season Infographic on the flipchart:
    a. Explain the infographic and what it shows.
    b. Ask if people are surprised by what is shown on the infographic (that people wash their faces a lot more in the dry season than at other times of year). Discuss.
    c. Get the group to conclude that they have enough water in their homes to wash faces 3x a day, even in the dry season, especially now that they have a wash station which makes it easier to wash and uses less water.
    d. Stick "extra people" on the infographic to show what we need to change to achieve *Faces of Dignity* all year round (i.e. extra face washes at midday and in the evening).
    e. Get the group to agree that they want to do this and can do this, to keep their *Faces of Dignity*.

Lack of Soap

1. Turn the flipchart – LACK OF SOAP page.
2. Ask participants to raise their hands if they feel that lack of soap is going to be an issue for them to practice face washing 3x a day with soap all year round.
3. Ask participants, regardless of whether they raised their hands, whether they have already found a way to make sure they always have soap for face and hand washing in their home. Discuss.
4. Thank participants for sharing their solutions. Explain that you would also like to share some solutions that communities nearby have come up with.

Soapy water demonstration

1. Explain that we can wash faces with soapy water if we do not have bar soap.
2. Ask anyone who has done this before to share their experiences
3. Demonstrate how to create soapy water using a plastic bottle.

Soapy water demonstration

1. Disinfect each empty plastic water bottle by spraying cleaning solution.
2. **Give every household an empty plastic water bottle of 1L.**
3. Put a small piece of soap in each bottle. Tell them that they will receive the rest of their soap bar at the end of the event.
4. Fill each bottle with water from the jerrycan.
5. Ask participants to shake their plastic bottle many times.
   →Tell them to stop here and leave it to dissolve for a day and finish the rest later.

6. Activator demonstrates how to finish making the soapy water after they have left the soap to dissolve
7. Shake the bottle vigorously again.
8. Check the water creates a good lather, if not, add more soap and shake again.
9. Make a hole in the lid of each plastic bottle using a nail.
10. Use the soapy water for face washing and use clean water to rinse. At the end of the demonstration, the activator reports how it feels to use the soapy water **"Oh, I am definitely using soap and not just water, I can smell it, and it feels different."**

4. Ask participants whether they feel they could do the same themselves.
5. Explain how the soapy water bottle can be attached using a string to the wash station. **Give a string to each household.**
6. Tell the group that soapy water can last a lot longer than the same amount of bar soap.
7. Ask whether anyone has any questions or other ideas about making soapy water.

Prioritising soap use for face washing instead of laundry, i.e. cutting soap

1. Explain that you know that soap is used for many things other than face washing and it can be hard to keep soap just for body washing.
2. Say that some people in nearby communities cut their soap in half so that soap can be kept in the soap dish and used just for face washing.
3. Ask anyone who has done this before to share their experiences.
4. Ask whether anyone has any questions or other ideas about dedicating soap for body washing.
5. Ask participants whether they feel they could do the same themselves.

Prioritising pre-school children for soap use

1. Tell participants to imagine that, despite using all the methods above to have soap, there is one day where there is soap for only one person in their house.
2. Ask participants whose *Face of Dignity* is the most important and who should be prioritised to get the soap. Discuss.
   a. If participants did not mention pre-school children, remind them that pre-school children are the ones having most discharge on their faces and the ones going around the community all day playing with their friends and going to neighbour's homes. Children are the faces of the family and their *Faces of Dignity* should be prioritised at all time.
3. Say that in life we always find a way to do something when it is important to us, and the *Faces of Dignity* of our family is really important so we know that everyone will work hard to make sure they always have soap.

Washing with water only

1. Say that despite all these solutions which can help to always have soap, it is possible that they might lack soap sometimes (e.g. have forgotten to buy a new one).
2. Advise participants to keep washing with water 3 times a day anyway. Tell them that they should not break their habit and feel discouraged. They should rather keep washing with water and reintroduce soap as soon as possible.

Lack of Attention

1. Turn the flipchart – LACK OF TIME AND FORGETFULNESS page.
2. Ask participants to raise their hands if they feel that lack of time or forgetfulness is likely to prevent them from practising face washing with soap x3 a day, all year round.

Forgetfulness

1. Explain that we often remember to do something when we see something in our home or environment that is connected to the activity we want to do.
2. Ask people how they remember to wash their hands before eating. Discuss.
3. Conclude that we can be reminded by lots of different things, such as the time of day, activities that always happen before or after, other people doing it or telling us to do it.
4. Say that the same can be true for face washing. We can help remind each other and we can always wash at fixed times of day, but we can also use visual reminders that make us think of face washing when we see them e.g. the "Dignified Day" poster, the wash station and soap dish, the soapy water bottle, etc.
5. If anyone has a phone, remind they could set alarms 3 times a day on their cell phone to help them to remember.
6. Ask participants whether they have any ideas of other things that could help them remember to wash their faces and their children's faces with soap 3x a day.

Lack of Time

Infographics

1. Explain to participants that many of their neighbours who complained about lack of time for face washing in the past, are now reporting that the face washing station has considerably changed the time it takes for face washing. It is really quick now.
2. Ask participants to raise their hands if they believe that the wash station makes it quicker to face wash.
3. Ask participants to raise their hands if they believe that lack of time will still be an issue for face washing at certain times during the year.
4. Turn the flipchart – PIE CHART 1 page.
    a. Show and explain the infographic
    b. Discuss the fact that face washing does not take much time compared to all the other activities they do every day
5. Turn the flipchart – PIE CHART 2 page.
    a. Show and explain the infographic
    b. Discuss that they do a lot of activities every day. The activities they choose to do are ones that are important. They can fit face washing in if they choose to prioritise it. Isn't it important to give our children the gift of a *Face of Dignity*?
    c. Ask the group whether they can make sure there is always a bit of time for face washing.

Other solutions

1. Discuss: sharing the responsibility of face washing with other adults in the households and older siblings.

2. Discuss: linking face washing with handwashing before meals – it is not really a separate activity.
3. Ask participants whether they have any questions or other ideas about how they can find time to prioritise helping their family achieve *Faces of Dignity*.
4. Encourage participants to try them all and find the solution which works at best for them.

Activity 6: Soap Giveaway

Purpose – Soap is consistently available and accessible for washing.

What to do

1. Turn the flipchart – Soap Giveaway page.
2. Tell participants that in addition to our tips and advice to always have soap, we are providing them with soap now because we really want them to be *Faces of Dignity* in their community.
3. **Give 1 body soap** to each family.
4. Remind them that a small part of it was used to make soapy water. Encourage them to try the soapy water and continue making it if they like it.
5. Tell participants that this soap could be put in the soap dish they got at the first Family Forum if their previous body soap is finished already or that they can store it in their home until their previous soap runs out.
6. Before moving on to the Conclusion activity, ask children to join back the forum. Congratulate children on their drawings and give one wax crayon to each child.

Activity 7: Conclusion

What to do

1. Turn the flipchart – Conclusion page.
2. Using the puppet, tell participants that we are now at the end of this family forum. Thank them for their participation.
3. Ask participants whether they have any questions or concerns.
4. Tell participants that the HV will come to visit them in their individual households in a week to see how they are doing, to answer any questions, and to check on their *Faces of Dignity*.
5. Remind participants that their Community cannot be dignified if all its members are not dignified. Their community's dignity will be publicly recognized and rewarded only if everyone in the community has a *Face of Dignity*.
6. Check that every family leaves with: **a soapy water bottle, a string and a body soap.**
7. Play the Dignity Song on a cell phone or any other device while participants are leaving the forum.
8. Put the puppet back into its cover.
9. Wash your hands with water and soap or alcohol-based sanitizer.

End of Family Forum 2.

Report Household Head Names of households who missed the forum on the Family Forum 2 Follow-up Visits Form before ending the event.
These households should be invited to join other Family Fora 2 held with other households in their cluster.
If they cannot join another session, individual follow-up with these families will be organised at their home.
Refer to Appendix A for details of the content to cover during this visit.

Appendices

Appendix A. "Follow-up with any households that do not attend the Family Forum 2"

HVs should do their best to ensure all households attend Family Forum 2 (in their original group or joining another group in their cluster). If any families do not attend, they will need to be followed up at home to go through the main activities of the forum and provide the extra giveaways (soapy water bottle, soap). If it is not possible to do this visit before the 1st House Call, the House Call should be extended so the extra content can be covered.

Setting

- Sit outside with the female primary caregiver and any family member present at home. Make sure the participants are comfortable, i.e. they should be sat in the shade.
- All materials required for the visit are organised and ready to be used.

Materials

General
- A cell phone or any other device to play the Dignity Song
- A facemask, alcohol-based sanitizer, sealable plastic bag for mask disposal

Soapy water demonstration
- 1L full water bottle
- 1 nail and 1 string
- 1 soap

Activities

Introduction

1. Wash your hands with soap or alcohol-based sanitiser and ensure you are wearing a facemask before entering the compound. Explain why you are wearing a facemask. If possible, stay outside to do the visit.
2. Greet the female primary caregiver / female adult. If they are not home **do not proceed: wait or return later.**
3. Say that the visit is short (15 mins) and ask her to gather any family members who are close by.
4. Tell the household you have come because they missed the second Family Forum and you would like to share with them what was discussed at the forum. Ask why they didn't attend.
5. Ask if they have any questions before you start your visit.

Building and using the station

1. Lead a discussion around the following topics to explore barriers and solutions to the construction of a wash station stand:
    a. Can you show us your wash station?
    b. How do you feel about your station? Should we take it away again or is it useful?
    c. Did you have any problems building it?
    d. Are you having any problems using it?
2. Discuss solutions to any problems that are raised.
3. Lead a discussion around the following topics to explore barriers and solutions to the use of the wash station for face washing with soap 3x a day:
   To the children (if applicable), ask:
    a. Are you using the wash station? If so, have you found it easier to wash your faces with soap now that you are using the wash station?
    b. Have you felt any difference on your skin now you are washing your face with soap regularly? Discuss issues around the skin becoming dry.

To the adults, ask:

c. *If there is a child in the household:* Are you using the wash station to wash your children's faces/your face?
d. Is it easy to use the wash station to wash your children's faces / your face *(if no child)* at all three times of day (morning, before lunch and before dinner), or is it not practical to use it at a particular time?
e. Have you found it hard to make sure that there is always water in the container? What is your solution? E.g. filling it as soon as water is collected.
f. What about making sure there is always soap in the soap dish and that this is kept at the wash station? What is your solution?
g. Are you moving your wash station inside at the end of the day? Why?
h. Have you been remembering to wash your children's faces / your face (if no child) three times a day, in the morning, before lunch and in the evening before dinner? What solutions have you found to help you to do this? E.g. setting an alarm, linking to the daily routine.

4. Discuss solutions to any problems that are raised.
5. Ask the household whether they have any remaining questions or comments related to the wash station or its use.

Troubleshooting issues

1. Ask the household whether they feel they might have some issues washing their faces 3x a day with soap using the wash station in the next days, weeks or even months. Discuss.
2. Say you would like to talk in a bit more detail about some of the issues that they have raised or that other people in their community have found to be problems, especially lack of water and lack of soap.
3. Play the water perception video. *NB. Might be removed if not feasible to show the video.*
    a. Ask people what they think the key message of this video is (Answer: that having water for face washing is more a matter of priority than an issue of scarcity).
4. Soapy water demonstration:
    a. Explain that we can wash faces with soapy water if we do not have bar soap. Ask the family whether they have ever done this before.
    b. Demonstrate how to create soapy water using a plastic bottle.

Soapy water demonstration

1. Disinfect the water bottle with by rubbing it with alcohol-based sanitizer.
2. **Give** the household **a full plastic water bottle of 1L**.
3. Put a small piece of soap in the bottle. Give the rest of the bar soap to the family and inform them it can be used at the station for face washing.
4. Ask the female primary caregiver / female adult to shake the plastic bottle many times.
   → Tell her to stop here and leave it to dissolve for a day and finish the rest later.
5. Explain that after the soap has dissolved, she should shake the bottle vigorously again.
6. Check the water creates a good lather, if not, add more soap and shake again.
7. Make a hole in the lid of each plastic bottle using a nail.
8. Use the soapy water for face washing and use clean water to rinse.
9. At the end of the demonstration, the activator reports how it feels to use the soapy water **"Oh, I am definitely using soap and not just water, I can smell it, and it feels different."**

    c. Explain how the soapy water bottle can be attached using a string to the wash station. **<u>Give a string</u>** to the participant.
    d. Say that soapy water can last a lot longer than the same amount of bar soap.
    e. Ask whether they have questions.
5. Quickly discuss other solutions to troubleshoot lack of soap:
    a. Prioritising soap use for face washing instead of laundry, i.e. cutting soap
    b. If there are children in the household: Prioritising pre-school children for soap use (the ones with most discharge on their face and being the faces of their families in the community)
    c. In worst case scenario, if soap is not available at all, to keep washing faces with water three times a day and reintroduce soap as soon as possible.
6. Ask the household whether they have any questions related to troubleshooting any issues.

Face washing demonstration

1. Ask the female primary caregiver / adult female to wash both her face and her pre-school child's face (if she has children) using the wash station.
2. Before she washes, ask her the following questions:
    a. How can you make sure to remove all visible and invisible discharge?
        i. Briefly discuss wiping: should be done with the hand, hands should be washed with soap immediately to remove discharge.
    b. How many times a day should you wash our children's faces / your face with soap to ensure they have *Faces of Dignity*? When?
    c. Whose *Faces of Dignity* are the most important?
3. After she has washed herself / her child, congratulate her on being a role model for her children to copy her behaviour and for supporting her child to have a *Face of Dignity*.
4. Hand washing with soap: *(households with children only)*
    a. If the mother washed the child's hands, congratulate the mother.
    b. If the mother did not wash the child's hands, ask her to do so now and remind her that face and hand washing with soap should always go together unless someone is washing their own face with their hands.
5. Ask every other family member present (including male adult) to wash their faces using the wash station. Play the Dignity Song while every family member is washing.
6. Congratulate all family members and say that washing our family's faces 3x a day with soap, washing our eyes and nose, and making sure the younger ones are prioritised and supported every time, will help us achieve *Faces of Dignity*, faces that will make us dignified and respected people.

"A Dignified Day" Poster check

1. Ask the household to show you the poster they were given at the last forum.
    a. <u>If the poster is put on the wall:</u>
        i. Congratulate the household.
        ii. Ask all the family members to raise their hands if they intend to keep doing these activities so that they continue to lead dignified lives, be *Faces of Dignity* in their community and contribute to enhancing their community's dignity.
    b. <u>If the poster is not on the wall:</u>
        i. Ask one of the children / female adult if he/she remember the dignified activities on the poster that help a family have a dignified day.
        ii. Advise the family to put the poster up as a way to remind them about doing these activities every day to lead dignified lives and being *Faces of Dignity* for their family and in the community.

Conclusion and Giveaways

1. Say that this is the end of the visit. Ask if they have any question or concerns. Thank them for their time.
2. Inform the household that HV will come to visit them at their home in a week to see how they are doing, to answer any questions, and to check on their *Faces of Dignity*.
3. Check that the household has received: **a soapy water bottle, a string and a body soap**.
4. Wash your hands with water and soap or alcohol-based sanitizer after leaving the compound. Ensure you are safely disposing your facemask in a sealable plastic bag at the end of the morning visits or at the end of the day.
