# Supplementary material for: Translating Formative Research into Intervention Content: Experiences with Face Washing for Trachoma Control in Rural Ethiopia
Source: Behav Sci (Basel). 2025 Mar 13;15(3):355. doi: 10.3390/bs15030355 (PMC11939790; doi:10.3390/bs15030355)
Supplement: Supplementary file 1 [file behavsci-15-00355-s001.zip › PDF files/05_House Call 1 Checklist_Paper.pdf]

EVENT 4: HOUSE CALL 1 – CHECKLIST

Household ID: ☐☐☐☐☐☐ Date*: ☐☐/☐☐/☐☐

Start time*: ☐☐:☐☐ End time*: ☐☐:☐☐ *Use Ethiopian calendar and times

Household Head Name: ____________________________ HV Name: ________________________________

Materials

- COVID-19 prevention: 1 facemask, alcohol-based sanitiser, sealable plastic bag to dispose of the mask
- 1 pen
- 1 dangler with 1 string and 1 nail
- 1 wash station flyer (if needed)
- A cell phone or any other device to play the Dignity Song

ACTIVITY 1: INTRODUCTION

1. Wash your hands with soap or alcohol-based sanitiser. Wear a facemask before entering the compound and explain why. If possible, stay outside to do the visit.
2. Greet the female (or male) primary caregiver. If they are not home do not proceed: wait or return later.
3. Say that the visit is short (20 mins) and is to see how they are getting on with their new wash station.
4. Ask the caregiver to gather any family members who are close by.
5. Ask if they have any questions before you begin.

ACTIVITY 2: FACIAL CLEANLINESS ASSESSMENT AND FACE WASHING

1. Ask who in the household has washed their face so far that day. Congratulate them if they all report having washed. Remind them that young children are the most important: The family cannot be truly dignified if the children are not dignified.
2. For the youngest child aged 1 – 6 years present at home, **observe and record**:

◯ Tick if 'No children aged 1 – 6 years were home' – Otherwise, record information below:

Age: ◯ ◯ years (enter 01 for age=1, 02 for age=2, etc.) Gender: ◯ Male ◯ Female

Face washing reported that morning: ◯ Yes ◯ No

Facial Cleanliness Assessment *(tick all that apply)*

◯ Ocular secretions present i.e. presence of clear or cloudy fluid, or dry matter on the lid margin or eyelid (including in the corners)

◯ Nasal secretions present i.e. presence of wet or dry discharge outside the nostril. Please do not stare up the nostril to find discharge but see the discharge visible outside the nares

◯ Any fly landing on face during examination

3. Ask the caregiver to ask the same child (aged 1- 6 years) to wash their face as they normally would. If a child this age is not present in the household, ask an older child or another household member to wash. Play the Dignity Song while the child is washing face.
4. During the demonstration, **record** the following:

Put an "X" in the box if the person washing: *(tick all that apply)*

◯ Uses the wash station ◯ Uses soap

If the person washing is a child aged 1-6 years, put an "X" if they: *(tick all that apply)*

◯ Are washed by someone else (e.g. mother, father, sibling).

◯ Wash/are washed thoroughly around the eyes and nose.

◯ Wash/have their hands washed.

The tap of the container was closed whilst the person washing was scrubbing his/her face:

◯ Yes ◯ No

5. Discuss any points on the checklist that are missed.

Activity 3: Wash station review

1. Tell the family that you would now like to have a look at their wash station.
2. Ask questions, **record** answers and discuss any issues:

Answer the following questions by marking with an "X": *(tick all that apply)*

- ◯ **Wash container** is present → If not, ask where it is: ____
- ◯ Wash container is **inside** OR ◯ Wash container is **outside**
- ◯ **Wash station stand** is present → Discuss height
- ◯ Wash container has **water** (open the tap to see whether water flows out of the tap)
- ◯ Wash container is **functional,** i.e. tap is working and not leaking, container is not damaged
- ◯ **Soap** is present
- ◯ **Soap dish** is present
- ◯ **Soapy water** is present

3. If the wash station stand has not been built, ask to see their wash station flyer and go through it. Give a new flyer if they cannot locate it.
4. If the wash station is damaged say you will report it.

Activity 4: "A Dignified Day" poster review

1. Ask to see the "Dignified Day" poster and **record** the following.
2. Poster present in home:

◯ No ◯ Yes, on wall → Discuss if too high ◯ Yes, not on wall → Discuss placing somewhere visible

Activity 5: Dangler giveaway

1. Ask if they have any problem remembering to wash faces and hands before meals.
2. Say that you have a gift to help them remember to wash faces and hands before lunch and dinner.

House dangler

3. Ask where they usually have their meals and ask to see the room.
4. **Give 1 dangler** to the family as a reminder to wash faces and hands before eating lunch and dinner.
5. Fix the dangler on the wall in a place indicated by the family. Use **the nail and rope**. **Record:**

Dangler put up in household: ◯ Yes, on wall → Discuss if too high ◯ No

Activity 6: Conclusion

1. Ask if they have any questions.
2. Say that someone will return in a week to see how they are getting on with using their wash station for face washing x3 a day as a family and to certify them as a dignified family.
3. Congratulate the household on their hard work to create new habits to maintain *Faces of Dignity* for their whole family, especially the youngest children who cannot maintain their dignity on their own.
4. Congratulate the household for contributing to enhancing their Community's dignity.
5. Thank the household for their time and end House Call 1.
6. Wash your hands with water and soap or alcohol-based sanitiser after leaving the compound. Ensure you are safely disposing of your facemask in a sealable plastic bag at the end of the morning visits or at the end of the day.

Please keep this checklist safe. Give it to the responsible person from Berhan when you next see them.
