# Supplementary material for: Translating Formative Research into Intervention Content: Experiences with Face Washing for Trachoma Control in Rural Ethiopia
Source: Behav Sci (Basel). 2025 Mar 13;15(3):355. doi: 10.3390/bs15030355 (PMC11939790; doi:10.3390/bs15030355)
Supplement: Supplementary file 1 [file behavsci-15-00355-s001.zip › PDF files/07_House Call 2 Checklist_Paper.pdf]

EVENT 5: HOUSE CALL 2 – DATA FORM

Household ID: ☐☐☐☐☐☐ Date: ☐☐/☐☐/☐☐ Time: ☐☐:☐☐
Ethiopian calendar *Ethiopian time*

Household Head Name: ______________________________ Activator Name: ___________________________

VIDEO DEMO

Age: ◯ ◯ years (enter 01 for age=1, 02 for age=2, etc.) Gender: ◯ Male ◯ Female

Put an "X" in the box if the person: *(tick all that apply)*

◯ Uses the wash station with tap
◯ Uses soap. If yes, which soap was used: ◯ Laundry soap ◯ Body soap ◯ Soapy water
◯ Is supported by someone (e.g. mother, father, sibling)
◯ Washes clearly around the eyes and nose
◯ Closes the tap whilst scrubbing his/her face
◯ Has hands washed (if face wash is performed by someone other than the child)

WASH STATION REVIEW

Water container with tap is present:
◯ Yes, inside ◯ Yes, outside ◯ Cannot be seen → Ask where the water container is
Record where it is: ______________________________

Family has built a station for their wash station water container:
◯ Yes, suitable height.
◯ Yes, too high → Discuss changing the height so it can be used by more family members.
◯ No → Discuss how they are using the material and how the station could help them.

There is water in the wash station container: ◯ No ◯ Yes, but no water flows when the tap is open
◯ Yes, 1/4 full ◯ Yes, half full or more

The wash station container is functional: ◯ Yes ◯ No, why: ______________________________________

There is bar soap at the station: ◯ Yes, in soap dish ◯ Yes, not in soap dish ◯ No

There is a soapy water bottle at the station: ◯ Yes, attached to the station ◯ Yes, not attached ◯ No

POSTER AND DANGLER REVIEW

Location of the poster: ◯ On wall, suitable height
◯ On wall, too high → Suggest changing location and explain why
◯ Present, but not visible → Suggest nailing to wall
◯ Not seen in home ◯ Other: _________________________________

Location of the dangler: ◯ Hung. If yes, where: ◯ In the kitchen ◯ Place people take meals
◯ Present in the house, but not visible → Suggest hanging the dangler
◯ Not seen in home ◯ Other: _________________________________

WASH STATION CERTIFICATION

Certification sticker has been given to household: ◯ Yes ◯ No
Certification sticker has been stuck on the wash station container or a dedicated material: ◯ Yes ◯ No

Please keep this checklist safe and give it to the responsible person at Berhan when you see them.
