# Supplementary material for: Translating Formative Research into Intervention Content: Experiences with Face Washing for Trachoma Control in Rural Ethiopia
Source: Behav Sci (Basel). 2025 Mar 13;15(3):355. doi: 10.3390/bs15030355 (PMC11939790; doi:10.3390/bs15030355)
Supplement: Supplementary file 1 [file behavsci-15-00355-s001.zip › PDF files/08_Reinforcement Events Manual_Paper.pdf]

REINFORCEMENT EVENTS
ACTIVATOR MANUAL

**Purpose**	Reinforce key messages and narratives of the Campaign (face washing with soap x3 a day, with an emphasis on preschool children) and overcome seasonal barriers to face washing behaviour.
**Responsible parties**	2x trained Activators and 1 Health Volunteer
**Participants**	All adults living within the 'yolk' of an intervention cluster – 10 household groupings
**Location**	TBD – A host household or a public space
**Duration**	45 mins to 1h (maximum)
**Timing**	Workdays (except Friday morning), 9am to 1pm and after 3pm
**Times of year**	Dry season: End of December/Early January for February (driest month in the year)<br>Rainy season: June for July/August (rainiest two months of the year)<br>Final: anytime

Preparation

Week before the start of the delivery

- Train Activators on the content of the Reinforcement Event.

Day before the event

- Provide all materials to the Activators or Health Volunteers.
- Mobilise Activators or Health Volunteers to invite 10x neighbouring households to the event based on the groupings of the Family Forums.
- Ask Activators or Health Volunteers to identify a suitable location for the event, ideally in the compound of one of the households or a public space.
- Communicate time and location of the event to each participating household.
- In the morning, ask Activators or Health Volunteers collect data on the status of wash stations within the cluster.
- *If applicable:* In the afternoon, ask Health Volunteers (HVs) to invite eight individuals, including themselves among kebele leaders, Health Extension Workers (HEWs), community leaders/elders, the kebele women and youth affairs leader, and religious leaders/influential role models. They will provide feedback on the status of their wash stations, engage in a brief discussion based on this feedback, and establish a direction for future improvements.
- *If applicable:* ask Activators to select role model HHs who will provide live testimonials on their experience of using wash stations to practise face washing with soap three times a day. They should also share strategies for overcoming barriers associated with this practice during the Reinforcement Event.

Setting

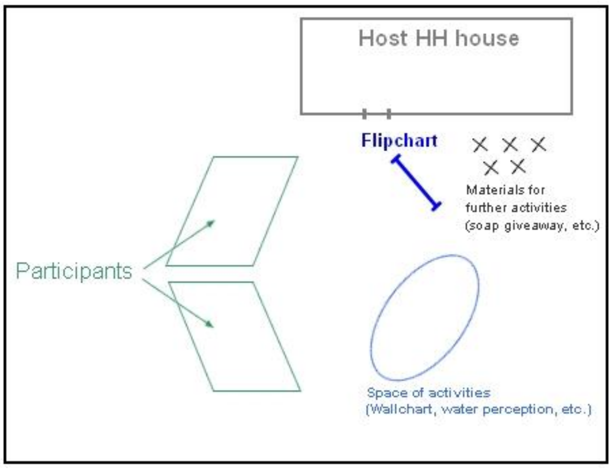

Materials

For all reinforcement events

General
- Flipchart (for use of specific pages + 5 Problem Solving storyboards) *(except for the Second Rainy Season Reinforcement)*
- 2 or 3 tablets to show the "Wash station maintenance" video (First Rainy Season Reinforcement), play the Dignity Song, and record data regarding face station maintenance (Second Rainy Season Reinforcement)
- Face wipes (approximately 1/child) + Green clay for face
- 2 jugs + 2 water collectors + 20L jerrycan full of water

Soap giveaways
- 10 body soaps (1 per household)

First rainy season reinforcement

Maintaining *Faces of Dignity*
- 7 Velcro flashcards

Water perception
- 1 full plastic water bottle of 1L
- 1 full jerrycan (water) of 5L
- 2 full plastic water bottles of 2 litres
- 2 full plastic water bottles of 1litres
- 2 full plastic water bottles of ½ litres

Soapy water
- 1 full plastic water bottle of 1L
- 1 body soap (to make soapy water demonstration + to be cut)
- 1 nail

Dry season reinforcement

Maintaining *Faces of Dignity*
- 7 Velcro flashcards

Wash station maintenance
- Socket set (including bi-hex and full hex) for wash station maintenance

Second rainy season reinforcement

Wash station maintenance
- Socket set (including bi-hex and full hex) for wash station maintenance

Soapy water – 1 full plastic water bottle of 1L
– 1 body soap (to make soapy water demonstration + to be cut)
– 1 nail

Final reinforcement

Campaign Reminder
– Laminated image of the *Faces of Dignity* Family (A3 or A2)
– *A Dignified Day* Poster

Role as a Role Model Short drama
– Two series of 10 laminated "Role as Role Models" flashcards (10 for men and 10 for women)
– Laminated colour images with the father's role, mother's role, and parent's role
– 2 jugs + 2 water collectors
– Clothing for Activators (funny attire)
– 20 blue and red cards

Soapy water
– 10 full plastic water bottles of 1L
– 10 body soap (to make soapy water demonstration + to be cut)
– 10 nails
– 10 small ropes
– Line drawing of Caltu's puppet (1 per child) + Wax crayons(1/child)

Set up

Day of the reinforcement event

- Do you have all materials required for all activities?
- Is equipment working?
  Tablets have full battery and the drama film is on each tablet.

EVENT 6 – REINFORCEMENT EVENT: RAINY SEASON
ACTIVATOR MANUAL

ACTIVITY 1: INTRODUCTION & SIGNPOSTING TO *THE FACES OF DIGNITY*

What to do

1. Set up the flipchart – REINFORCEMENT EVENT: RAINY SEASON COVER page.
2. Play the Dignity Song on a tablet or cell phone while participants are arriving.
3. Greet the group and welcome them to the event.
4. Advise community members to sit with their household members and respect a physical distance of at least 2m with other households.
5. Ask for a show of hands to see who remembers the *Faces of Dignity* campaign.
6. Invite one or two people to share what they remember from the campaign (be sure to correct them if anything they say is incorrect)
7. Say that you are here to continue your discussions about face washing with soap.
8. Say that the event will not be long and they will receive a gift at the end.

Round table

1. Raise your hand if you feel you have a *Face of Dignity*. Ask volunteers to comment on why they feel this
2. Raise your hand if you feel your children have *Faces of Dignity* that represent your family. Ask volunteers to comment on why they feel this
3. Raise your hand if you feel your community have a *Face of Dignity*. Ask volunteers to comment on why they feel this

4. Confirm the key messages of the *Faces of Dignity* campaign through Q & A with the group:
   a. **Why do we need to wash our faces?** Face washing with soap helps maintain beauty and dignity and thus helps gain respect in the community and enhance the community' dignity.
   b. **How many times a day should faces be washed?** Faces should be washed with soap x3 a day: after waking in the morning, before lunch and before the evening meal.
   c. **When should soap be used during face washing?** Always!
   d. **Which family members should wash their faces with soap x3 a day?** All of them! Young children should be assisted to wash so that eyes and noses can be thoroughly cleaned of any dirt and trachoma-carrying discharge. Soap is essential to remove discharge. Face washing should be a familial activity, where pre-school children are prioritised but everybody is washing to set a good example for the younger ones
   e. **Why do we need to use soap when we wash our faces?** To remove the discharge that can contain trachoma and stop it from spreading to the eyes of other members of our family.

A Dignified Day

1. Turn the flipchart – A DIGNIFIED DAY page.
2. Remind participants that earlier in the Campaign we agreed on a list of daily activities which help us gain *Faces of Dignity* and maintain our family's *Face of Dignity*. These activities were listed on the posters they were given.
   a. Ask the group:
      i. Do you remember the dignified activities on the poster that help a family have a dignified day?

ii. If they cannot remember all the activities, show them any outstanding flashcards, ask the group what the image represents, and put the flashcard on the flipchart.

b. Ask the whole group to pledge to keep doing these activities so that their families can continue to lead dignified lives, achieve *Faces of Dignity* and keep enhancing their Community's dignity

Trachoma Transmission Routes

1. Turn the flipchart – TRACHOMA TRANSMISSION ROUTES page.
2. Show the group the TRACHOMA TRANSMISSION DIAGRAM and discuss routes of transmission.

ACTIVITY 2: FACE WIPE EMO-DEMO – SHORT VERSION

What to do

1. Ask participants/children if they see any difference between the face of the two Activators.
2. Ask if the faces look clean.
3. Explain to participants that you are going to use wipes to confirm what they have just said. Explain that the wipe is just a cloth that is a bit wet.
4. Wash your hands with soap before starting.
5. Ask participants which wipe they expect to be dirty.
6. The activators should wipe the RIGHT side of their faces and show the group the wipes
7. Ask the audience what they see: both wipes are dirty. Is this a surprise?
8. Say that faces can look clean even when they are not. Say that we need to use soap to get them really clean and dignified.
9. Ask the audience if they agree with this

ACTIVITY 3: PROBLEM IDENTIFICATION & TESTIMONIALS

What to do

1. Ask the group what problems they expect to face continuing to wash their faces with soap x 3 a day in this season.
2. Ask if they have any challenges using the wash station. Listen and facilitate the discussion for a few minutes.
3. Tell the group we are going to discuss some of the main challenges they have raised and think about how they can be overcome.
4. Turn the flipchart – PROBLEM SOLVING STORYBOARD page.

Live Testimonials

1. Invite the role models HHs to comment on the problems raised by the community during discussion.
2. Ask them to share their experiences of how they have overcome the obstacles/challenges raised by the community and how they are washing their hands and face with soap three times a day using this washing station.
3. Ask the audience if they agree with this and tell them that there is something we can do together to prove what they have just mentioned. *NB: invite role model HHs to respond on from the same cluster, (if possible) who provide statement testifying their experiences of using wash station' on reinforcement event one day before event.*
4. Invite Role Model HHs to in front of gathered people to share their experience of using wash station to practice face washing with soap 3x a day and testifying of how to overcome barriers associated with this practice to become dignified family. How important their behaviour is and how it also reflects well on the community.

5. Tell the participants that you are going to discuss some of the main obstacles they have raised
6. Use the storyboard between each activity below to help structure the discussion about problems and solutions.

Activity 4: Problem-Solving – Lack of Time

What to do

1. Explain to participants that many of their neighbours who complained about lack of time for face washing in the past, are now reporting that the face washing station has considerably changed the time it takes for face washing. It is really quick now.
2. Say that you will prove it with a race:
    a. Ask for two volunteers. One needs to wash their face with soap using a HW station, the other needs to wash their face with soap in the way they do when they don't use a HW station.
    b. Everyone can see that the person who used the HW station finished washing much faster than the other person
3. End the activity by reminding people that if they put water in the wash station and soap nearby they can easily wash their faces all day long.
4. Discuss that they do a lot of activities every day. The activities they choose to do are ones that are important. They can fit face washing in if they choose to prioritise it. Isn't it important to give our children the gift of a *Face of Dignity*?

Other solutions

1. Sharing the responsibility of face washing with other adults in the households and older siblings.
2. Linking face washing with handwashing before meals – it is not really a separate activity.
3. Ask participants whether they have any questions or other ideas about how they can find time to prioritise helping their family achieve *Faces of Dignity*.
4. Encourage participants to try them all and find the solution which works at best for them.

Activity 5: Problem-Solving – Forgetfulness

What to do

1. Turn the flipchart – Lack Of Time and Attention page.

Forgetfulness

1. Explain that we often remember to do something when we see something in our home or environment that is connected to the activity we want to do.
2. Give suggestions: put the wash stations somewhere you will see it when you go to eat so you remember to wash face and hands, put an alarm on your phone if you have one, help remind each other, put the dignified day poster somewhere you will see it regularly,
3. Ask participants whether they have any ideas of other things that could help them remember to wash their faces and their children's faces with soap 3x a day. When there is no water, advise participants to keep washing with water 3 times a day anyway.
4. Tell them that they should not break their habit and feel discouraged. They should rather keep washing with water and reintroduce soap as soon as possible.
5. Ask participants whether they have any remaining questions or ideas related to soap and keeping soap at the station.
6. Encourage participants to try them all and find the solution which works best for them. Say that in life we always find a way to do something when it is important to us, and the *Faces of Dignity* of our family is really important so we know that everyone will work hard to make sure they always have soap.

ACTIVITY 6: PROBLEM SOLVING – WASH STATION USE AND MAINTENANCE

What to do

1. Ask for a show of hands of any families with problems making sure there is water in their wash station.
2. Explain that they don't need to fill it up completely in order to use it as that would take too much water. Turn the flipchart and show them picture of wash station container
3. Discuss who in the family is responsible for checking the water and putting it in the wash station and who will help them to remember to do it.
4. Ask for a show of hands if anyone has any problems with their wash station, is the tap working OK still?
5. Play the Wash Station Maintenance video on the tablet. *NB. This will be amended according to feasibility.*
6. Repeat the video if the group requests it.
7. Discuss the video and answer any questions about replacing broken or leaky taps.

ACTIVITY 7: PROBLEM SOLVING – LACK OF WATER

What to do

1. Turn the flipchart – LACK OF WATER page.

Water perception demonstration

1. Tell the participants that we have spent time observing how people in the community use water for different activities in the household.
2. Take out your cups/bottles with different quantities of water in them.
3. Say that these quantities of water correspond to daily activities. Show them the laminated pictures of these activities (cooking, doing dishes, coffee making/washing coffee cups and face washing with soap).
4. Get the group to guess which amount of water corresponds to which activity. They should place the picture cards in front of the cup/bottle they think it relates to.
5. When the group has finished, tell them their order starting from minimum amount of water they think a typical family uses to perform that activity daily. For instance, if you need to so it reads like this:
    a. Face washing with soap (work out what quantity is minimum you can use)
    b. Coffee making and other coffee-related activities – 3 litres
    c. Cooking – 4 litres
    d. Doing dishes – 4 litres
6. Draw their attention to the smallest amount of water with the picture of face washing with soap. Ask one volunteer HHs members (average 5 persons) to wash their faces with the water in the bottle (1liter) with soap to prove it can be done with very little water. Tell them that 5 family members can use (amount of water they used to wash their faces with soap multiply by three) amount of water to wash their faces with soap three times a day.
7. Ask participants to raise their hands if they agree that other daily activities consume more water than a whole family face washing.
8. Reinforce message that even when water is scarce they have enough for face washing 3x a day as it does not consume much water.
9. Tell participants that water is sometimes scarce and we should teach our children to use it carefully and to turn off the tap on the wash station when water is not needed.

ACTIVITY 8: PROBLEM-SOLVING – LACK OF SOAP

What to do

1. <u>Turn the flipchart</u> – LACK OF SOAP page.

Soapy water demonstration

1. Remind the group that you gave them bottles and they made soapy water.
2. Discuss experiences making and using soapy water.
    a. Who used the soapy water they made?
    b. Who made more when the soapy water ran out?
3. <u>Say that you will do a quick demonstration to remind the group how to make soapy water, for those who find it useful</u>.

> **Soapy water demonstration**
> 1. Take a full plastic water bottle of 1L.
> 2. Put a small piece of soap in the bottle.
> 3. Shake the plastic bottle many times.
>   → Tell participants that, when they are preparing soapy water, they should stop here and leave it to dissolve for a day and finish the rest later.
>
> 4. HV demonstrates how to finish making the soapy water after they have left the soap to dissolve.
> 5. Shake the bottle vigorously again.
> 6. Check the water creates a good lather, if not, add more soap and shake again.
> 7. Make a hole in the lid of each plastic bottle using a nail.
> 8. Use the soapy water for face washing and use clean water to rinse.
> 9. At the end of the demonstration, the HV reports how it feels to use the soapy water **"Oh, I am definitely using soap and not just water, I can smell it, and it feels different."**

ACTIVITY 9: SOAP GIVEAWAY

What to do

1. **<u>Give</u>** each household **<u>with 1 body soap</u>**. Encourage them to put it in their soap dish and use it only for body washing.

ACTIVITY 10: CONCLUSION

What to do

1. <u>Turn the flipchart</u> – CONCLUSION page.
2. Tell participants that we are now at the end of this Reinforcement Event.
3. Thank them for their time and participation.
4. Ask whether they have any remaining questions or concerns.
5. Remind the group that we will host other events like this in the coming months.
6. Play the Dignity Song while participants are leaving the event.
7. Wash your hands with water and soap or alcohol-based sanitizer.

End of Rainy Season Reinforcement.

EVENT 7 – REINFORCEMENT EVENT: DRY SEASON
ACTIVATOR MANUAL

ACTIVITY 1: INTRODUCTION & SIGNPOSTING TO *THE FACES OF DIGNITY*

What to do

1. Set up the flipchart – REINFORCEMENT EVENT: DRY SEASON COVER page.
2. Play the Dignity Song on a tablet or cell phone while participants are arriving.
3. Greet the group and welcome them to the event.
4. Advise community members to sit with their household members and respect a physical distance of at least 2m with other households.
5. Ask for a show of hands to see who remembers the *Faces of Dignity* campaign.
6. Invite one or two people to share what they remember from the campaign (be sure to correct them if anything they say is incorrect)
7. Say that you are here to continue your discussions about face washing with soap.
8. Say that the event will not be long and they will receive a gift at the end.

Round table

1. Raise your hand if you feel you have a *Face of Dignity*. Ask volunteers to comment on why they feel this
2. Raise your hand if you feel your children have *Faces of Dignity* that represent your family. Ask volunteers to comment on why they feel this
3. Raise your hand if you feel your community have *Face of Dignity*. Ask volunteers to comment on why they feel this

4. Confirm the key messages of the *Faces of Dignity* campaign through Q & A with the group:
   a. **Why do we need to wash our faces?** Face washing with soap helps maintain beauty and dignity and thus helps gain respect in the community and enhance the community' dignity.
   b. **How many times a day should faces be washed?** Faces should be washed with soap x3 a day: after waking in the morning, before lunch and before the evening meal.
   c. **When should soap be used during face washing?** Always!
   d. **Which family members should wash their faces with soap x3 a day?** All of them! Young children should be assisted to wash so that eyes and noses can be thoroughly cleaned of any dirt and trachoma-carrying discharge. Soap is essential to remove discharge. Face washing should be a familial activity, where pre-school children are prioritised but everybody is washing to set a good example for the younger ones
   e. **Why do we need to use soap when we wash our faces?** To remove the discharge that can contain trachoma and stop it spreading to the eyes of other members of our family.

A Dignified Day

1. Turn the flipchart – A DIGNIFIED DAY page.
2. Remind participants that earlier in the Campaign we agreed on a list of daily activities which help us gain *Faces of Dignity* and maintain our family's *Face of Dignity*. These activities were listed on the posters they were given.
   a. Ask the group:

    i. Do you remember the dignified activities on the poster that help a family have a dignified day?
    ii. If they cannot remember all the activities, show them any outstanding flashcards, ask the group what the image represents, and put the flashcard on the flipchart.
b. Ask the whole group to pledge to keep doing these activities so that their families can continue to lead dignified lives, achieve *Faces of Dignity* and keep enhancing their Community's dignity

Trachoma Transmission Routes

1. Turn the flipchart – TRACHOMA TRANSMISSION ROUTES page.
2. Show the group the TRACHOMA TRANSMISSION DIAGRAM and discuss routes of transmission.

ACTIVITY 2: DEMONSTRATION OF THE BENEFITS OF WASHING WITH SOAP

What to do

1. HVs and activator both paint of their faces with clay. When applying, they should apply so that participants cannot see them. They both stand in front of the participants and ask what their faces look like.
2. Ask participants/children if they see any difference between the face of the two Activators. Participants will be asked to raise their hands and tell them what they understand
3. They both start washing their faces saying that you will follow us to see what we are going to do.
4. The activator washes his face and hands thoroughly with soap and water. When washing, he should wash his face back to the participants.
5. HVs washes his face and hands with water only. When he washes, he should wash slightly so that the dirt remains on his face. He should wash his face back to the participants
6. After washing, they both stand in front of the participants and ask them to tell them the difference between the two faces. If the participants raise their hands and say, "The face washed with soap and water is clean and there is no dirt on it," the activator should say and has to confirm that "yes, my face is clean. I feel very happy." If the participants raise their hands and say "there is visible dirt on the face washed with water only." HVs should say and has to confirm that "yes, it is true that washing the face with water alone does not clean the dirt and discharge properly as you can see now. This can easily expose us to trachoma."
7. Ask the audience if they agree with this

   Conclusion: The activator will conclude the following points:
    a. As we have been teaching you on face emo demo in the past during the family forum, our face can be dignified and respectful and dirt can only be removed properly if we wash it with soap.
    b. Please confirm that you agree with this idea by raising your hand. Conclude by confirming that participants agree

ACTIVITY 3: PROBLEM IDENTIFICATION AND TESTIMONIALS

What to do

1. Ask the group what problems they expect to face continuing to wash their faces with soap x 3 a day in this season.
2. Ask if they have any challenges using the wash station. Listen and facilitate the discussion for a few minutes.
3. Tell the group we are going to discuss some of the main challenges they have raised and think about how they can be overcome.

4. Turn the flipchart – PROBLEM SOLVING STORYBOARD page.
5. Live testimonial: Invite the role models HHs to comment on the problems raised by the community during discussion.
6. Ask them to share their experiences of how they have overcome the obstacles/challenges raised by the community and how they are washing their hands and face with soap three times a day using this washing station.
7. Ask the audience if they agree with this and tell them that there is something we can do together to prove what they have just mentioned. *NB: invite role model HHs to respond on from the same cluster, (if possible) who provide statement testifying their experiences of using wash station' on reinforcement event one day before event.*
8. Invite Role Model HHs to in front of gathered people to share their experience of using wash station to practice face washing with soap 3x a day and testifying of how to overcome barriers associated with this practice to become dignified family. How important their behaviour is and how it also reflects well on the community.
9. Tell the participants that you are going to discuss some of the main obstacles they have raised
10. Use the storyboard between each activity below to help structure the discussion about problems and solutions.

ACTIVITY 4: PROBLEM-SOLVING WASH STATION USE AND MAINTENANCE & DISCUSSION ON PERCEPTIONS OF ROLES (MEN AND WOMEN)

What to do

Wash station maintenance

1. Ask for a show of hands if anyone has any issues with their wash station. Is the tap still working properly?
2. Instead of showing a wash station maintenance video, each activator should be provided with a socket set (including bi-hex and full hex) to demonstrate wash station maintenance during every group discussion with 10 households. To carry out this activity, the activator should follow these steps:
    a. He/she should have a wash station container with a drain/leak.
    b. Stand in front of the participants and show the participants how to repair/maintain the wash station container. After the maintenance/repair, he/she will show the participants that the container does not leak. If their wash station is leaked water, he/she will remind participants that they should repair/maintain it accordingly
    c. Repeat the how to repair/maintain the wash station container if the group requests it.
    d. Discuss the repair steps and answer any questions about replacing broken or leaky taps.
    e. Inform participants that socket sets will be available in their clusters through a volunteer, and if they encounter similar issues, they can ask the volunteer for assistance with repairs.

Perceptions of roles (men and women)

1. The activator will conduct an open discussion in the form of Q and A with the participants on the following points: The activator will let the participants to share their family experiences by raising their hands
    a. **Wash station container taking out and in**: Who in your household member monitors the wash station to take out in the morning and take in in the evening?
    b. **Replacing soap:** How are you replacing soap when it runs out? Who in your family is given responsibility?

c. **If the wash station runs out of water**: Who in your household members is refilling the wash station container regularly before it runs out?
d. **If the mother travels**, who in your family will make /help the young children (children who cannot wash on their own) to wash their face and hands?
e. The activator will conclude based on the comments given from participants

ACTIVITY 5: PROBLEM-SOLVING – LACK OF WATER

What to do

1. Turn the flipchart – LACK OF WATER page.

Water perception demonstration

1. Tell the participants that we have spent time observing how people in the community save water using their wash station.
2. This activity should be done in a competitive manner between Activator and HVs.
3. The activator should wash his face and hands with soapy water using wash station.
4. The HVs should wash his hands and face using a water collector and jug
5. The activator should save water when washing face and hands. When washing, he/she should use soap watery hanging near the wash station container. Activator should use water and less time while washing
6. The HVs should wash with excess water when washing. He also has to find and bring a water collector, jug, water and soap before washing. HVs should use more water and time while washing
7. To determine the amount of water they use to wash their faces and hands. When they both wash, they must wash carefully so that the waste water does not fall off the ground and has to be collector on water collector
8. Both activator and HVs wastewater should be placed in a half-litre bottle and shown to participants. Ask participants to voluntarily raise their hands and tell the difference between the waste water in the two bottles. Participants should appreciate the differences they see.
9. Ask participants if they agree that using wash station container saves water and that many families can wash with less water. One person washing face and hands using water collector consumes more water and time than a whole family face washing. Make sure they agree.
10. Remind them that using a wash station is a solution to their water shortages and lack of time so that they can save water and time.
11. Reinforce message that even when water is scarce they have enough for face washing 3x a day as it does not consume much water.
12. Tell participants that water is sometimes scarce and we should teach our children to use it carefully and to turn off the tap on the wash station when water is not needed.

ACTIVITY 6: PROBLEM-SOLVING – LACK OF SOAP

What to do

1. Turn the flipchart – LACK OF SOAP page.

Live testimonials: Soapy water

1. Remind the group that you gave them bottles and they made soapy water.
2. Discuss experiences making and using soapy water.
   a. Who used the soapy water they made?
   b. Who made more when the soapy water ran out?
3. Invite volunteer HHs from participant who is currently using wash station properly to show the following points to participants:

4. He/she prepares his/her own soapy water and will show the participants. He/she tells participants that soapy water is very easy to prepare
5. He/she tells to participants that more than twenty one-litre bottles of soapy water can be prepared with a bar of soap
6. He/she used to prepare soapy water from a bar of soap and tell the participants that a bar of soap will serve them for more than two months.

Conclusion

1. Ask "Do you agree with what they just told us?" to ensure that the participants agree. Ask participants to confirm it by raising their hands in agreement.
2. As people have just told us, it is very easy to prepare soapy water.
3. We have learned from model HHs that many soapy waters can be prepared from a bar of soap and that a bar of soap can be used for at least two months. I would like to ask other participants to apply the same procedure as we have just seen.

Activity 7: Problem-Solving – Procrastination: Short Drama

What to do

1. Turn the flipchart – PROCRASTINATION page.

Procrastination

1. Play the short Procrastination Drama

The Activator, acting as a volunteer, goes to follow up with a household that has not set up their wash station outside.

Aba Chala:
Good morning/afternoon, Hadha Caltu!
Hadha Caltu:
Good morning/afternoon, Aba Chala. What brings you by? Are you well? Come in, please.
Aba Chala:
Haven't you put the wash station outside today, like you did the last couple of days?
Hadha Caltu:
Thank you, I'm well. Do come in. Apologies – I got caught up with something just now. I was about to finish it and then take the wash station outside.
Aba Chala:
Hadha Caltu, let's sit and have a word about this. I'm here for the third day now, and I still haven't seen any change.
Hadha Caltu:
I know, I just have this small task in hand, then I'll get right to it.
Aba Chala:
Alright then, I'll wait while you finish.

After a few minutes…
Aba Chala:
Are you ready now?
Hadha Caltu:
Yes, what is it you wanted to discuss?

Aba Chala:

Hadha Caltu, I've come by for three days now. What's stopping you from setting up the wash station outside?

Hadha Caltu:

You're right, you've come each day, and each time, I've said I'd finish what I'm doing and put it out.

Aba Chala:

Please, tell me honestly – what's really keeping you from setting it up? Are you and your family actually washing your hands and faces with soap and water?

Hadha Caltu:

Lying is forbidden, and yes, we try to keep up with it as best we can.

Aba Chala:

But if the wash station isn't even out, how are you all washing properly? What's the real issue?

Hadha Caltu:

You're right. They taught us how to use it, showed us how to use soap – we understand it well enough. But like the old saying goes, "A habit doesn't leave a person, just like a hill doesn't move."

Aba Chala:

So you know the benefits, but what's really getting in your way, Hadha Caltu?

Hadha Caltu:

It's this procrastination – every day I say I'll do it, and then I push it off. I even meant to put it out today before heading to the hospital.

Aba Chala:

Is everything alright? What's taken you to the hospital?

Hadha Caltu:

I haven't slept all night – my eyes sting, and it feels like they're burning. I haven't had a wink of sleep.

Aba Chala:

Please, go and wash them with soap.

Hadha Caltu:

Yes, I'll get up and do just that.

After washing...

Hadha Caltu:

Goodness! It's like a miracle! I feel so much better – if only I'd washed like this three times a day, I wouldn't be in this state.

Aba Chala:

That's exactly the point. From now on, keep the wash station outside and wash with soap and water three times daily, for yourself and your family.

Hadha Caltu:

You're right. From today, I promise we'll wash three times a day – once in the morning when we wake, before lunch, and again before dinner.

Aba Chala:

That's good, Hadha haltu. Let's make it a habit – washing with soap and water three times a day will help prevent trachoma. Take care!

Hadha Caltu:

Thank you, Aba Chala, for looking out for us. Have a good day.

ACTIVITY 8: SOAP GIVEAWAY

What to do

1. **Give** each household **with 1 body soap**. Encourage them to put it in their soap dish and use it only for body washing.

ACTIVITY 9: CONCLUSION

What to do

1. Tell participants that we are now at the end of this Reinforcement Event.
2. Thank them for their time and participation.
3. Ask whether they have any remaining questions or concerns.
4. Remind the group that we will host other events like this in the coming months.
5. Play the Dignity Song while participants are leaving the event.
6. Wash your hands with water and soap or alcohol-based sanitizer.

End of Dry Season Reinforcement.

EVENT 8 – REINFORCEMENT EVENT: RAINY SEASON

ACTIVATOR MANUAL

Purpose Focus on getting the faces of pre-school children washed with soap and providing resources for wash station maintenance. Continue to address lack of soap and water and encourage role modelling. Reinforce key intervention's messages.

Implementers Health Volunteer x1

Participants All individual households in intervention 'yolk'. Individual household visits followed by small gatherings of 5 to 10 neighbouring households.

Duration 15 minutes in individual households followed by 30 minutes in small groups.

Location Appropriate host house or public space that is not too noisy / distracting.

Timing Workdays (except Friday morning), 9am to 1pm and after 2pm. On the first day in each cluster, Activators will replace damaged wash stations and assist in constructing wash station stands using locally available materials.

INDIVIDUAL HOUSEHOLD'S EVENT (15 MINS)

ACTIVITY 1: WASH STATION CHECKS

What to do

1. Open the data record form "Wash station check" on the tablet.
2. Observe the wash station in each individual home and complete the form:

1. **Observe:** is the wash station present in the home?
NB. This question is about the physical presence of the wash station, not whether it is functional / in use.
◯ Yes, inside ◯ Yes, outside ◯ No
Record where it is: ___________________
2. **Observe:** if yes, does the wash station have water?
Turn the tap and check if water comes out.
◯ Yes, but water is below the level of the tap
◯ Yes, water flows when the tap is turned
◯ No
3. **Observe:** is the wash station functional?
The wash station could be used to wash hands and faces if it had water in it e.g. there is a stand of some kind, the tap is working and not leaking, the container is not damaged etc. This question is about functionality, not use.
◯ Yes *(go to question 5)*
◯ No *(go to question 4 and skip questions 5, 6 and 9)*
4. **If no, ask:** why is the wash station not functional? *Select all that apply.*
◯ No stand ◯ Tap is broken ◯ Tap is leaking ◯ Container is damaged
◯ Other: ________________
5. **Observe:** is soap present? ◯ Yes ◯ No *(go to question 7)*
6. **If yes, record:** which kind of soap is present?
◯ Soapy water ◯ Other kinds of soap

7. **Observe:** is a soap dish present? ◯ Yes ◯ No
8. **Ask:** is there soap available elsewhere in the home?

Ask the caregiver to bring the soap for you to see it.

◯ Yes (seen) ◯ Yes (not seen) ◯ No

9. **Observe:** is there water on the ground or in a water collector below the wash station?

Does the wash station appear to be in use.

◯ Yes *(go to question 11)* ◯ Unsure *(go to question 10)* ◯ No

10. **Ask:** for which purposes are you using this wash station? *Select all that apply*

◯ Body washing ◯ Other purposes ◯ Not being used

11. **Ask:** Which of the following challenges do you face using the wash station?

Read out each challenge and select all that apply.

◯ Lack of soap
◯ Lack of water in the home
◯ Filling the wash station with water (when there is enough water)
◯ Absence of wash station stand
◯ Fear of theft of wash station
◯ Do not find it easy to use
◯ Prefer to wash in another way
◯ Other
◯ None of the above

3. Thank the household for responding to your questions.

Group Event (30 mins)

ACTIVITY 1: Wash station Maintenance

What to do

1. Instead of showing a wash station maintenance video, demonstrate wash station maintenance using the provided socket set (including bi-hex and full hex). Follow/demonstrate the following steps:
    a. Stand in front of the participants and show the participants how to repair/maintain the wash station container. After the maintenance/repair, show the participants that the container does not leak. If their wash station is leaked water, remind participants that they should repair/maintain it accordingly
    b. Repeat the how to repair/maintain the wash station container if the group requests it.
    c. Discuss the repair steps and answer any questions about replacing broken or leaky taps.
    d. Inform participants that socket sets will be available in their clusters, and if they encounter similar issues, they can ask you for assistance with repairs.

ACTIVITY 2: Demonstration of the Benefits of Soap

What to do

1. Paint your face with clay without being seen by the household members – rub it in so the face looks clean.
2. Gather the family and tell them you are going to explain why soap is important.
3. Ask them if your face looks clean (they should agree that it does).

4. Wash the left side of your face with water only, and the right side with water and soap. Explain what you are doing as you do it. *(Use your own soap and water).* Remind participant how little water you are using and catch the waste water in a bowl.
5. Wipe the left side of your face with a face wipe and show the family that it is dirty. Remind them that you washed this side of your face with just water. Wipe the right side of your face with another face wipe and show the family that it is clean. Remind them that you washed this side of your face with water and soap.
6. Ask participants to explain what they have understood: you need to use soap to get a face truly clean. Tell them you are an adult and your face is this dirty. Say that it is even more important to wash our children's faces and to use soap as children get more dirty than adults and washing faces is an easy way to maintain dignity.
7. Tell the family that you know that they do not always have soap, and when they do not have soap they should still wash their face and their children's faces as using water only is better than not washing at all.

Activity 3: Problem-Solving – Lack of Soap

What to do

1. Ask participants if they remember that they can make soapy water if they find it hard to always have enough soap in the home.
2. Take your bottle with water and cut up bits of bar soap in it. Show them the small bits of soap and the water and remind them how to make the soapy water.

Soapy water demonstration

1. Take a full plastic water bottle of 1L.
2. Put a small piece of soap in the bottle.
3. Shake the plastic bottle many times.
   → Tell participants that, when they are preparing soapy water, they should stop here and leave it to dissolve for a day and finish the rest later.
4. HV demonstrates how to finish making the soapy water after they have left the soap to dissolve.
5. Shake the bottle vigorously again.
6. Check the water creates a good lather, if not, add more soap and shake again.
7. Make a hole in the lid of each plastic bottle using a nail.

Activity 4: Wash-Along

What to do

1. <u>Ensure all pre-school children and their caregivers join and participate in this activity.</u>
2. Before the children are washed by their caregivers, make sure that there is soap and water in the same place. If they do not have any soap, give them a small soap.
3. Invite all participant dyads (caregivers and children) to wash their faces with soap.
4. When the faces have been washed, continue:
   a. Ask the gathered participants if the faces look clean, i.e. there is no discharge around the eyes or nose. If needed, ask them to wash the face again.
   b. Ask the gathered participants to recognise that the mother can wash the child's face better than the child can do it themself. Say that young children often wash their own faces, but they do not rub well around the eyes and nose. It is better to do it for them so we know their face is truly clean and dignified.
   c. Comment on how much easier it is to wash a child's face when the soap and water are kept together (at the wash station).

d. Remind the caregivers that they can save water by using the wash station, but they need to turn the tap off.
e. Say that children's faces should always be washed when they wake up and before they go to bed. We should do this even if there is no soap.

Activity 5: Conclusion

What to do

1. Tell participants that we are now at the end of this Reinforcement Event.
2. Thank them for their time and participation.
3. Ask whether they have any remaining questions or concerns.
4. Remind the group that we will host other events like this in the coming months.
5. Wash your hands with water and soap or alcohol-based sanitizer.

End of Second Rainy Season Reinforcement.

Event 9 – Final Reinforcement Event

Activator Manual

Purpose Focus on getting the faces of pre-school children washed with soap, with family role modelling to ensure all family members also wash faces with soap. Continue to address lack of soap and water and encourage role modelling.

Implementers Activators x2 (1 to lead the men's event and 1 to lead the women's event before the groups merge into a Whole Family Event) with support of x2 Health Volunteers.

Participants All intervention families living in 'yolk' households. Kids should attend the women's group so it is easy to make sure they are present when the groups are merged for the joint session.

Duration 30 minutes for separate groups, followed by up to 1 hour in the combined group.

Location Appropriate host house or public space that is not too noisy / distracting.

Timing Workdays (except Friday morning), 9am to 1pm and after 2pm. On the first day in each cluster, Activators will replace damaged wash stations and assist in constructing wash station stands using locally available materials.

Men's Event (30 mins)

Activity 1: Introduction & Campaign Reminder

What to do

1. Greet the group and welcome them to the event.
2. Ask participants: what do you remember from our previous sessions? *Community Event, Family Forums, House Calls and two Reinforcement Events.*
3. Ask for a show of hands:
   a. Who remembers the *Faces of Dignity* campaign?
4. Put a large A3/A2 image of the *Faces of Dignity* family in the middle of the group.
5. Ask volunteers to share what they remember about the *Faces of Dignity* campaign. Be sure to correct them if they say anything wrong.
6. Recap the key messages through Q & A with the group:
   a. **Whose face should be washed?** Faces of all family members should be washed.
   b. **Whose faces wash needs to be assisted and prioritized?** Young children should be assisted to wash so that eyes and noses can be thoroughly cleaned of any dirt and trachoma-carrying discharge. Soap is essential to remove discharge. Face washing should be a familial activity, where pre-school children are prioritised but everybody is washing to set a good example for the younger ones.
   c. **What times of day should faces be washed?** Faces should be washed with soap x3 a day: after waking in the morning, before lunch and before the evening meal.
   d. **When should soap be used?** Always! But if you don't have any still wash faces!
   e. **Why do we need to use soap when we wash our faces?** To remove the discharge that can contain trachoma and stop it from spreading to the eyes of other members of our family.
5. Talk about dignity, ask the group:
   a. What types of activities help you maintain dignity?
      i. Show the group the Dignified Day Poster or Flipchart page (large images) to help the discussion.

   b. Raise your hand if you feel you have a *Face of Dignity*. Ask volunteers to comment on why they feel this.
   c. Raise your hand if you feel your children have *Faces of Dignity* that represent your family in the community. Ask volunteers to comment on why they feel this.

Activity 2: Role as a Role Model

What to do

1. Ask the group what a role model is. Who are your role models from your family and why?
2. Ask the group if they believe they are important role models for their children.
3. Show the group a series of cards depicting activities to do with face washing and wash station maintenance. The cards to be piloted are as follows:
   a. Role modelling face washing by washing own face with soap at same time as children.
   b. Making sure there is budget to have soap in the home at all times.
   c. Notice when soap / soapy water is running low and inform the person who will replace it so there is always soap at the wash station.
   d. Purchasing soap when the soap is running low (before it runs out).
   e. Making soapy water when the soap is running low (before it runs out).
   f. Making sure the wash station is kept in the location where it is used (e.g. outside) every day, not just when someone is coming to check it.
   g. Making sure the wash station stand is properly made and durable.
   h. Making sure the wash station is well maintained and used properly so it isn't damaged.
   i. Making sure there is water in the wash station and replacing it when it runs out.
   j. Supporting each other as a family to wash faces with soap together in the morning and evening (times when they are all together).
   k. Washing children's faces for them.
4. Ask them to group the cards into three groups to show whose role they are: the father's role; the mother's role; or both parents' role.
   a. Stick each card into the column they select on the flipchart using velcro.
   b. When they have finished sorting the cards into groups, discuss any cards that you feel are in the wrong group and try to persuade the group to regroup them.

Activity 3: Problem-Solving – Wash Station Maintenance

What to do

1. Wash station stands
   a. Show the group an A3 or larger laminated image of a good, durable wash station stand that has been made with locally available materials (no cost).
   b. Using the laminated image discuss how to make a sustainable stand like this. Discuss drainage. Discuss where the stand will be located.
2. Maintenance of taps (leaking / breaking).
   a. Who has problems with their wash station, is the tap working OK still?
   b. Remind the group how to avoid breaking the taps. This should have just been discussed in Activity 2.
   c. Go through the following maintenance points using a wash station container with a drain/leak:
      i. Stand in front of the participants and show the participants how to repair/maintain the wash station container. After the maintenance/repair, he/she will show the participants that the container does not leak. If their wash station is leaking water, he/she will remind participants that they should repair/maintain it accordingly

ii. Repeat the how to repair/maintain the wash station container if the group requests it.
iii. Discuss the how to repair/maintain and answer any questions about replacing broken or leaky taps.

Activity 4: Problem-Solving – Lack of Soap

What to do

1. Ask volunteers to share why it is important to use soap or soapy water. Be sure to correct them if they say anything wrong.
2. Remind the group that in our previous discussion about the preparation of soapy water we confirmed that a bar of soap can be used for more than two months.
3. Ask for a show of hands: Who provides the budget for purchasing soap and is ultimately responsible for soap being available in the household?
4. Look at the tasks related to soap on the flipchart. Go back through the cards that the group agreed were men's responsibilities related to soap.
    a. Remind your wife, elder son, or daughter to regularly prepare soapy water and ensure that it is always available next to the wash station
    b. Remind everyone in the family to wash their faces with soap using a wash station
    c. Give money to the wife if it runs out.
    d. Supporting children to wash faces with soap, and role modelling this for them.

Women's Event (30 mins)

Activity 1: Introduction & Campaign Reminder

What to do

1. Greet the group and welcome them to the event.
2. Ask participants: what do you remember from our previous sessions? *Community Event, Family Forums, House Calls and two Reinforcement Events.*
3. Ask for a show of hands:
    a. Who remembers the *Faces of Dignity* campaign?
4. Put a large A3/A2 image of the *Faces of Dignity* family in the middle of the group.
5. Ask volunteers to share what they remember about the *Faces of Dignity* campaign. Be sure to correct them if they say anything wrong.
6. Recap the key messages through Q & A with the group:
    a. **Whose face should be washed?** Faces of all family members should be washed.
    b. **Whose faces wash needs to be assisted and prioritized?** Young children should be assisted to wash so that eyes and noses can be thoroughly cleaned of any dirt and trachoma-carrying discharge. Soap is essential to remove discharge. Face washing should be a familial activity, where pre-school children are prioritised but everybody is washing to set a good example for the younger ones.
    c. **What times of day should faces be washed?** Faces should be washed with soap x3 a day: after waking in the morning, before lunch and before the evening meal.
    d. **When should soap be used?** Always! But if you don't have any still wash faces!
    e. **Why do we need to use soap when we wash our faces?** To remove the discharge that can contain trachoma and stop it spreading to the eyes of other members of our family.
7. Talk about dignity, ask the group:
    a. What types of activities help you maintain dignity
        i. Show the group the DIGNIFIED DAY POSTER or Flipchart page (large images) to help the discussion.

   b. Raise your hand if you feel you have a *Face of Dignity*. Ask volunteers to comment on why they feel this.
   c. Raise your hand if you feel your children have *Faces of Dignity* that represent your family in the community. Ask volunteers to comment on why they feel this.

Activity 2: Role as a Role Model

What to do

1. Ask the group what a role model is. Who are your role models from your family and why?
2. Ask the group if they believe they are important role models for their children.
3. Show the group a series of cards depicting activities to do with face washing and wash station maintenance. The cards to be piloted are as follows:
   a. Role modelling face washing by washing own face with soap at same time as children.
   b. Making sure there is budget to have soap in the home at all times.
   c. Notice when soap / soapy water is running low and inform the person who will replace it so there is always soap at the wash station.
   d. Purchasing soap when the soap is running low (before it runs out).
   e. Making soapy water when the soap is running low (before it runs out).
   f. Making sure the wash station is kept in the location where it is used (e.g. outside) every day, not just when someone is coming to check it.
   g. Making sure the wash station stand is properly made and durable.
   h. Making sure the wash station is well maintained and used properly so it isn't damaged.
   i. Making sure there is water in the wash station and replacing it when it runs out.
   j. Supporting each other as a family to wash faces with soap together in the morning and evening (times when they are all together).
   k. Washing children's faces for them.
4. Ask them to group the cards into three groups to show whose role they are: the father's role; the mother's role; or both parents' role.
   a. Stick each card into the column they select on the flipchart using velcro.
   b. When they have finished sorting the cards into groups, discuss any cards that you feel are in the wrong group and try to persuade the group to regroup them.

Activity 3: Soap!

What to do

1. Ask volunteers to share why it is important to use soap or soapy water. Be sure to correct them if they say anything wrong.
2. Remind the group that in our previous discussion about the preparation of soapy water we confirmed that a bar of soap can be used for more than two months
3. Ask for a show of hands: Who takes on the responsibility of regularly buying soap for their family when it runs out?
4. Look at the tasks related to soap on the flipchart. Go back through the cards that the group agreed were men's responsibilities related to soap.
5. Go back through the cards that the group agreed were women's responsibilities. Show the women how many tasks relate to soap.
   a. Regularly prepare soapy water and ensure that it is always available next to the wash station
   b. Remind everyone in the family to wash their faces with soap using a wash station
   c. Prioritise washing faces of young children with soap
   d. Ask husband for money for soap if it runs out.

WHOLE FAMILY EVENT

NB. Ensure that children (especially preschool children) are present (hopefully they will have already been with the women in the previous session so it is quick to merge the two groups into family units.

ACTIVITY 1: ROLES & RESPONSIBILITIES

What to do

1. Tell the group that the sessions they just participated in had very similar content, looking at roles and being a role model.
2. Bring out the flipcharts from the men's and women's groups and show the group what was discussed. The purpose of this is to get everyone to take accountability for their responsibilities in front of their husband/wife/community.
3. Discuss each card.
    a. Ask those people for suggestions of how each task can be achieved.
4. Conclude the activity :
    a. Ask the group if they notice how many of the tasks are their role / the role of both parents. Tell them they need to support their wives so the whole family has *Faces of Dignity*.
    b. Tell the group that even if they are succeeding, no one is perfect and they can always improve.
5. Get the group to look at the cards and decide which 1 thing they will absolutely take away from this session and implement at home. This should be done individually, they may not all focus on the same thing. Go around the group and get each person to say out loud what they will do and how they will make sure they achieve it *[this is like a pledge to each other – saying it out loud gives them some accountability].*
6. Show the *Faces of Dignity* family image, printed large and laminated. Ask the group whose face should be washed if there is only enough water and soap to wash one face. If they don't say the youngest child, remind them that young children are the most likely to get dirty faces and to have trachoma and their faces should be washed to stop trachoma spreading to other people.

Public pledge

1. Say that the family must work together to ensure everyone washes their faces with soap, adults should wash their own faces to model the behaviour for children.
2. Ask each family to hold hands to form a circle.
3. Ask them to repeat the pledge out loud: ***"A clean face is attractive, it is also dignifying. I promise to do my part to make sure there is always soap and water available for face washing in our household. I will wash my face with soap as an example for our children. We will support each other to wash our children's faces."***
4. Ask each family to discuss how they will support each other.

Conclusion

1. Conclude the activity by congratulating the families for working together to maintain their family's dignity.

Activity 2: Success Testimonials

What to do

1. Acknowledge that there are barriers that they all experience related to lack of soap and water and remembering to wash faces. Ask for volunteers to share how they are overcoming these barriers and succeeding to wash their children's faces with soap.
2. Ask a household (HH) currently utilizing the wash station to provide a testimonial about their successful experience.
   NB. If none of the HHs present are using the wash station, showcase photos on the flipchart depicting other HHs from the same or different clusters using the wash station.
3. Remind the gathered HHs to utilize the wash station in a similar manner as other community members.

Activity 3: Get These Children's Faces Washed (with soap)!

What to do

Drama

1. Do the **short drama**. *Activators are wearing funny attire and introduce themselves, e.g. one is the father, one the mother.* Select two children aged 4 and 8 from the gathered children. They are a *"family"*. Ask the family to wash their faces in front of the group:
   a. Give *"the family"*a bowl, a jug with water and small bar of soap. Show the group how much water there is.
   b. Instruct the family to wash their own faces, and not to help their children.
   c. Use the following script for this family, but make it fun and engaging:

Aba Caltu:
Oh, what a bright morning! A new day's here, everyone!
Hadha Caltu:
Yee! (meaning "yes").
Aba Caltu:
Hadha Caltu, can you bring me some water so I can wash my face?
Hadha Caltu:
Ishii, (meaning "okay").
Aba Caltu:
I'm in a rush to get to meditation, Hadha Caltu. Make sure the whole family follows suit and washes their faces with soap.
Hadha Caltu:
Caltu, my daughter, have you seen the jug? Where's the water? Where's the soap? I put the bowl right here yesterday – why did someone move it? Every day it's the same search! Who used the soap last?

Hadha Caltu searches around the house for each of the items.

Aba Caltu:
Hadha Chaltu, why are you always so busy? Please, I'm telling you, I'm in a hurry!
Hadha Caltu:
Apologies, Aba Caltu. Here you go. I couldn't find the things where I left them – someone must have moved it all.
Aba Caltu:
What can we do? We always waste time looking for everything.

Aba Caltu quickly washes his face with soap and heads out for meditation, giving a quick, casual goodbye to the family without much affection.

Hadha Caltu washes her face, then places the soap on top of the wash station and tells the 4- and 8-year-old children to wash their faces.
The children struggle a bit. The 4-year-old tries to reach the soap, but after a few attempts, gives up and washes only with water, spilling some from the jug. The 8-year-old manages to grab the soap, applies it to her face, then reaches for the jug. Seeing it nearly empty, she shouts at the 4-year-old for spilling the water. The two exchange some cross words.

2. Now ask the volunteer family to wash their faces again, with a few changes:
    a. Give the family a wash station and soap in a soap dish placed on a wash station stand so it is easily accessible.
    b. Instruct the family to wash their own faces and then the faces of any preschool age children.
    c. Use the following script for this family.

Aba Caltu: *Muttering to himself.*
Right, up bright and early to get the wash station outside. Soapy water's ready, hanging from the wash station, and the soap's in the dish.
Aba Caltu washes his face with soap at the wash station, then calls out.

Aba Caltu:
Hadha Caltu, come on out and wash your face with soap!
Hadha Caltu: *Hadha Caltu steps outside, washes her face with soap, then calls to her children.*
Come on, children, time to wash faces!

She carefully helps her 4-year-old, washing their face with soap, making sure they're clean. She then invites her 8-year-old to wash on their own, which they manage with no trouble. The family all chat happily together, sharing a warm hug before Aba Chaltu heads off for the day.

Voting exercise

1. Give each person in the group two laminated voting cards. One blue (on both sides) with a smiley face (= easy) and one red (on both sides) with a sad face (= difficult).
2. Ask them to answer the following questions about the face washing they have just observed by voting with their cards by holding up the side of the card. Discuss the reasons why people voted as they did after each question:
    a. Was it easy (blue) or hard (red) for the family to wash their faces together?
    b. Did young children manage to wash their faces thoroughly (blue) or could they have washed them better (red)?
    c. Was it quick (blue) or did it take a long time (red) for the family to wash?
    d. Was it easy (blue) or hard (red) for everyone to find and use the soap?
    e. Did it use a little water (blue) or a lot of water (red)?
3. Ask the group to comment on what they have observed. Prompt them if needed to discuss the following points:
    a. When young children wash on their own they use a lot of water
    b. When young children wash on their own they find it hard to use soap properly
    c. When soap and water are not kept together it takes longer to wash and is more awkward
    d. When the wash station is used it makes it easier to wash young children's faces properly

Conclusion

1. Conclude that helping young kids to wash uses less water, keeps the soap clean, and gets faces cleaner so they are dignified and make the family dignified for each other and the community

Activity 4: Recap

What to do

1. Ask the group to shout out the one change each individual is going to make to support their family to wash their faces with soap x3 a day.

Activity 5: Soap Giveaway & Soapy water bottles

What to do

1. Instruct all families to prepare soapy water and take it with them. If it runs out instruct them to replenish it accordingly.
2. The children can colour in Caltu instead.

Activity 6: Conclusion

What to do

1. Tell participants that we are now at the end of this Reinforcement Event.
2. Thank them for their time and participation.
3. Ask whether they have any remaining questions or concerns.
4. Thank them for taking part of the *Faces of Dignity* Campaign
5. Play the Dignity Song while participants are leaving the event.
6. Wash your hands with water and soap or alcohol-based sanitizer.

End of Final Reinforcement.
